# Neurotherapeutic implications of sense and respond strategies generated by astrocytes and astrocytic tumours to combat pH mechanical stress

**DOI:** 10.1111/nan.12774

**Published:** 2021-12-09

**Authors:** Sebastian John, Gayathri K. G., Aswani P. Krishna, Rashmi Mishra

**Affiliations:** ^1^ Brain and Cerebrovascular Mechanobiology Research, Laboratory of Translational Mechanobiology, Department of Neurobiology Rajiv Gandhi Centre for Biotechnology Thiruvananthapuram Kerala India; ^2^ Manipal Academy of Higher Education (MAHE) Manipal Karnataka India

**Keywords:** astrocytes, cholesterol, glioblastoma therapy, GM3, lateral compressive mechanical forces, pH stress, tumour microenvironment, tumour sense–respond stress signalling

## Abstract

**Aims:**

Astrocytes adapt to acute acid stress. Intriguingly, cancer cells with astrocytic differentiation thrive even better in an acidic microenvironment. How changes in extracellular pH (pHe) are sensed and measured by the cell surface assemblies that first intercept the acid stress, and how this information is relayed downstream for an appropriate survival response remains largely uncharacterized.

**Methods:**

*In vitro* cell‐based studies were combined with an *in vivo* animal model to delineate the machinery involved in pH microenvironment sensing and generation of mechanoadaptive responses in normal and neoplastic astrocytes. The data was further validated on patient samples from acidosis driven ischaemia and astrocytic tumour tissues.

**Results:**

We demonstrate that low pHe is perceived and interpreted by cells as mechanical stress. GM3 acts as a lipid‐based pH sensor, and in low pHe, its highly protonated state generates plasma membrane deformation stress which activates the IRE1‐sXBP1‐SREBP2‐ACSS2 response axis for cholesterol biosynthesis and surface trafficking. Enhanced surface cholesterol provides mechanical tenacity and prevents acid‐mediated membrane hydrolysis, which would otherwise result in cell leakage and death.

**Conclusions:**

In summary, activating these lipids or the associated downstream machinery in acidosis‐related neurodegeneration may prevent disease progression, while specifically suppressing this key mechanical ‘sense‐respond’ axis should effectively target astrocytic tumour growth.

## Graphical abstract

In this manuscript, we have delineated the molecular steps by which normal and neoplastic astrocytes adapt to microenvironmental acidosis. The major highlights of the study are as follows: (1) High extracellular proton concentrations (pHe) generate mechanical stress on the astrocytes. (2) GM3 glycosphingolipid acts as a plasma membrane resident low pHe mechanical stress sensor and transducer. (3) GM3 activates the master controller protein IRE1, which further recruits the sXBP1‐SREBP2‐ACSS2‐cholesterol machinery to enable effective anti‐acidosis survival responses in brain astrocytes and astrocytic tumours. (4) IRE1 activation is neuroprotective in brain acidosis associated pathology. (5) IRE1 inactivation along with the additional depletion of surface cholesterol via STF‐083010 + Amphotericin B combination drug is promising in acidosis driven astrocytic tumour therapeutics.

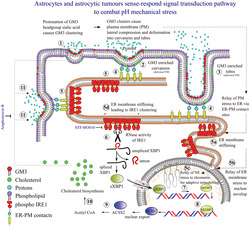

**Abbreviations**
ACSS2: Acyl‐coenzyme A synthetase short‐chain family member 2
ATCC: American Type Culture Collection
DNAJB9: (DnaJ Heat Shock Protein Family [Hsp40] Member B9)
DNAJC3: DnaJ Heat Shock Protein Family (Hsp40) Member C3
EGFP: Enhanced Green Fluorescent Protein
ER: Endoplasmic reticulum
ER‐PM: Endoplasmic reticulum and Plasma Membrane contact sites
FRET: Förster resonance energy transfer
GBM: Glioblastoma multiforme
GM1: monosialotetrahexosyl ganglioside
GM3: monosialodihexosyl ganglioside
HMGCR: 3‐Hydroxy‐3‐Methylglutaryl‐CoA Reductase
IRE1: Inositol‐requiring enzyme 1
LactCer: Lactosylceramide
LAMP2: Lysosome‐associated membrane protein 2
LDHA: Lactate dehydrogenase A
LDL: low density lipoprotein
LUT: Look Up Table
MTT: 3‐(4,5‐dimethylthiazol‐2‐yl)‐2,5‐diphenyl tetrazolium bromide
PBS: phosphate buffer saline
PFA: paraformaldehyde
pHe: extracellular pH
PI: Propidium Iodide
PMT: photomultiplier tubes
RT: Room Temperature
SNA: *Sambucus Nigra* Lectin
SREBP2: Sterol regulatory element‐binding protein 2
STED: Stimulated emission depletion
sXBP1: spliced form of X‐Box Binding Protein 1
WGA: wheat germ agglutinin

**Key points**

- High extracellular proton concentrations (pHe) generate mechanical stress on the astrocytes.
- GM3 glycosphingolipid acts as a plasma membrane resident low pHe mechanical stress sensor and transducer.
- GM3 activates the master controller protein IRE1, which further recruits the sXBP1‐SREBP2‐ACSS2‐cholesterol machinery to enable effective anti‐acidosis survival responses in brain astrocytes and astrocytic tumours.
- IRE1 activation is neuroprotective in brain acidosis associated neurodegenerative pathologies.
- IRE1 inactivation along with the additional depletion of surface cholesterol via STF‐083010 + Amphotericin B combination drug is promising in acidosis driven astrocytic tumour therapeutics.

## INTRODUCTION

Regulation of brain tissue microenvironmental pH (pHe) is critical for neurophysiological processes [1], failing which, the organism can succumb to various acidosis‐associated disorders from cancers to degenerative diseases such as ischaemia and Alzheimer's disease. Involvement of low pH is also proposed in several psychiatric disorders, for example, panic attack, anxiety and depression [2, 3, 4, 5, 6, 7, 8]. Hence, there is a strong need for brain cells, especially the astrocytes, as nurturing cells to maintain a dense network of pH sensing machinery for homeostasis. However, until now, astrocytes' pH sensing and downstream response machinery have not been sufficiently well understood to enable its therapeutic exploitation.

Putative pH sensing proteins consist of protonation amenable amino acids such as histidine that can change the overall charge of proteins, leading to conformational changes [9]. These structural changes can induce H^+^/HCO_3_^−^ ion transport through adaptive signal cascades that have not yet been defined.

pH sensing mechanisms are not limited to proteins as better structural flexibility of certain surface lipids (negative charge, tilt and torsion) can enable them to act as robust pH metres. Protonation susceptible surface lipids such as phosphatidic acid (PA) and phosphoinositides (PIPs) can ‘switch conformation’ upon changes in pH. These structural changes allow different proteins to interact with the phospholipids, which can dramatically impact cellular metabolism and signalling [10]. However, the evidence for these phenomena is obtained from yeast and drosophila and is not understood in detail in higher mammalian systems.

The pH‐sensing role of lipids and pH‐dependent lipid‐protein interactions are not limited to PA and PIPs. Gangliosides are negatively charged glycosphingolipids that bear protonable sialic acid moieties in the sugar headgroup. Gangliosides are enriched in specialised membrane domains called ‘lipid rafts’. Ganglioside topography can play crucial roles in raft size, stability, composition, membrane tension, in‐plane lateral compression, membrane deformation and cellular signal transduction [11, 12]. Indeed, proton concentrations directly impact the lateral organisation and dynamics of ganglioside containing microdomains in model membrane systems [13].

Since the plasma membrane acts as the prime interface in eukaryotes at which the extracellular milieu is sensed and from which signals are relayed for an effective adaptive response, the lipid bilayer and its resident lipids should play a crucial role in the pHe sense‐response axis. In this pHe adaptation machinery, a major responder is expected to arrive on the surface where pHe fluctuations were initially sensed and expected to persist until the microenvironment returns to a normal physiological state.

Hence, to establish the molecular steps by which normal and neoplastic astrocytes adapt to microenvironmental acidosis, we designed experiments to delineate sensors and responders involved in acid stress combating machinery [14, 15, 16, 17, 18, 19]. A snapshot of the experimental design of the work is presented in Figure S1 for quick reference of the various experimental phases.

## MATERIALS AND METHODS

Additional details on reagents and standard protocols are available in the supporting information file accompanying this article.

### Cell culture with pH treatments

To assess the effects of low pHe, the pH of the media was altered with 2 N HCl (hydrochloric acid) in accordance with previously published protocols [15, 16, 17].

SVG human astrocytes (NHA1) [20] were seeded for 16 h in MEM medium with 10% FBS and 1X antibiotic‐antimycotic solution. NHA2 astrocytes, purchased from Lonza, were cultured in the medium and growth factors provided with the cells. Mouse primary astrocytes were isolated from P5 pups according to the protocol described by Schildge et al [21]. GBM tumour cell lines (U87MG, LN229 and U373) were cultured in DMEM high glucose‐containing medium, supplemented with 10% FBS and 1X antibiotic/antimycotic solution. The cells were treated with MEM medium adjusted to pH 7.4, 6.8, 6.2 and 5.8 for 4 h unless otherwise indicated. Fresh pH‐adjusted media was added to cells every 2 h of treatment. Tumour cell lines were treated likewise unless otherwise indicated in specific assay protocols.

In experiments involving the reversal of pH to physiological values, after 4 h of initial treatment, the low pH media was replaced with buffer solution [Normal Medium Supplement (pH 7.4)] and kept as such for the next 4 h, and then cells were fixed with 1.5% PFA (pH 7.4).

### FRET‐based force sensor studies

The FRET probe, Actinin‐sstFRET‐GR ([22, 23, 24] Addgene#83416, EGFP‐mCherry FRET pairs were tagged at the N‐C termini of spectrin linker, and this DNA fragment inserted between the actinin‐head domain and actinin‐tail domain), was transfected into the cells using the jet PRIME transfection method. Post transfection, cells were incubated for 36 h and then pH treatments (pH 7.4, 6.8, 6.2, 5.8) were given for the next 4 h, followed by cell fixing and mounting. For a detailed experimental set‐up, please refer to the Supplementary file. To obtain FRET efficiency values, the initial intensity of each cell was normalised. FRET efficiency was calculated using the formula: ([I_(FR AB)_] − I_FR_) /I_(FR AB)_, where I_FR_ (FRET Ratio) = Intensity of donor/Intensity of acceptor (donor excited acceptor emission), I_(FR AB)_ = Intensity of donor after photobleaching/Intensity of acceptor after photobleaching. This value was then plotted for each condition. This value can also be expressed in percentage by multiplying the ratio by 100.

### GM3 lipid homo‐FRET

For GM3‐GM3‐homo FRET: GM3‐biotin and TopFluor‐GM3 were fed to cells in equimolar concentrations (3.75 μM) for 1 h at RT before pH treatments. Cells were then given pH treatment (pH 7.4, 6.8, 6.2, 5.8) and fixed with PFA. Rhodamine Red conjugated streptavidin was incubated on the surface to bind to biotin‐GM3 to enable its visualisation. The FRET assay was performed with the same microscopy set‐up as described in the previous section. TopFluor GM3 is labelled as TF‐GM3, Rhodamine red GM3 is labelled as RR‐GM3 in the figures.

In GM1‐GM3 and LactosylCer‐GM3 FRET, 3.75 μM biotin‐GM3 was fed to cells in equimolar concentration to BodipyFL GM1 or LactCer‐BodipyFL for 1 h at 37°C. pH treatments were subsequently followed for 4 h, and the cells were fixed. Rhodamine Red conjugated streptavidin was incubated on the surface to bind to biotin‐GM3 to enable its visualisation. Note that BodipyFL and Rhodamine Red are efficient FRET pairs. TopFluor and Rhodamine Red are also efficient donor‐acceptor FRET pairs. BodipyFL GM1 is labelled as BD‐GM1, and BodipyFL Lactosylceramide is labelled as BD‐LactCer in figures. Peak excitation and emission wavelengths of the FRET fluorophores are as follows: TopFluor Ex:495 nm, Em:503 nm; Bodipy FL Ex:503 nm, Em:511 nm; Rhodamine Red X Ex:572 nm, Em:591 nm.

### sXBP1 generation inhibition

60,000 cells were seeded in 8 well chamber slides and were incubated for 36 h. Cells were then treated with the IRE1 RNAse activity inhibitor (STF‐083010) at a concentration of 60 μM for 6 h [25]. Cells were given pH treatments (pH 7.4, 6.8, 6.2, 5.8) for the next 4 h along with the inhibitor. Post‐treatment, cells were either processed for respective assays or were fixed with 1.5% PFA for 20 min at RT and washed with 1XPBS‐ 4 times.

### Mouse brain acidification studies

Brain acidification was performed with an excess CO_2_ inhalation method. The protocol followed has been as described in Magnotta et al [14] but with some modification. Six mice pups (C57BL/6) at day 5 after birth were first injected subcutaneously with STF‐083010 (10 mg per kg body weight as described in [25]) in 50 μL saline over the dorsal cranium. Another set of 6 pups were injected with saline alone. 15 mins after injection, 3 saline‐injected and 3 STF‐083010 injected pups were transferred to a 7% CO_2_ incubator for CO_2_ inhalation for 2.5 h. Another six pups (three saline‐injected and three STF‐083010 injected) were kept in a normal atmospheric environment. After 2.5 h, pups were sacrificed by hypothermia, and the brains were immediately dissected and fixed overnight in 4% PFA at 4°C. The following day, excess fixative was removed by washing the brains with 1X PBS and processed for paraffin embedding. The brains were sectioned in the sagittal plane at a thickness of 10 μm. Near mid‐sagittal sections were chosen for further analysis.

### Statistical analysis

Statistical analyses were performed using one‐tailed unpaired Bonferroni's *t* test. Significance was represented by the following *p* values: *p ≤ 0.05, **p ≤ 0.01 and ***p ≤ 0.001. All comparisons were made with pH 7.4 conditions unless otherwise indicated. Data is presented as means ± S.D. and averaged from at least three independent experiments.

## RESULTS

### Low extracellular pH (pHe) acts as a biomechanical force‐generating agent on astrocytes' surfaces causing the plasma membrane to compress laterally

**FIGURE 1:**
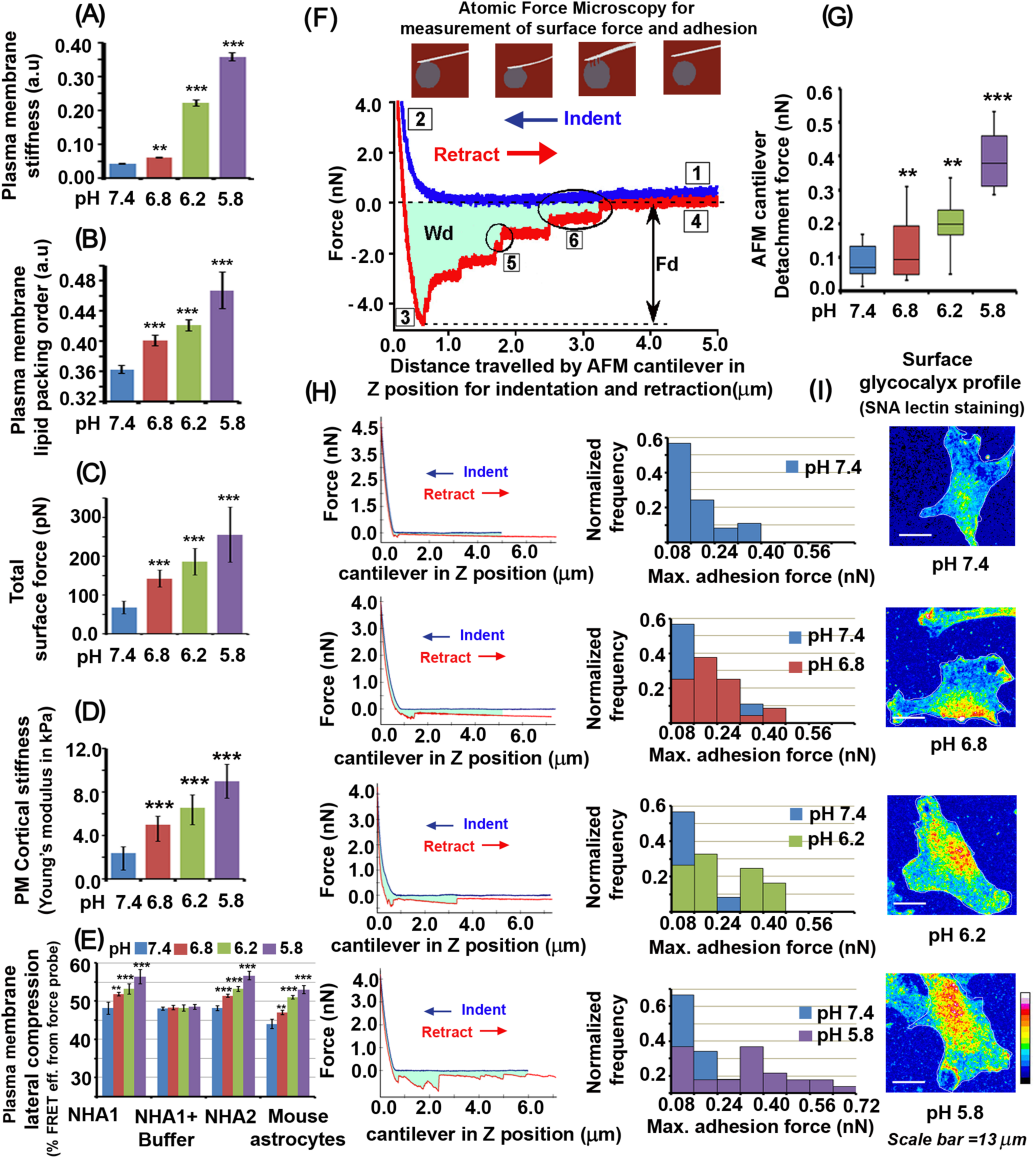
Low extracellular pH (pHe) triggers stiffness and associated biomechanical remodelling in the astrocyte plasma membrane. All studies were performed on passagable astrocyte cells (SVG/NHA1) to demonstrate that low extracellular pH (pHe) acts as a biomechanical force‐generating agent on astrocytes unless otherwise indicated. (A) An increase in DPH dye fluorescence anisotropy, indicating plasma membrane reduced fluidity, was observed in astrocytes treated with low pH media. DPH is a lipophilic molecule that is preferentially incorporated and retained in high lipid packed membrane regions because such membrane organisation causes a restriction in its movement, orienting it in a plane parallel to the bilayer; hence it acts as a reliable membrane stiffness sensing fluorescent probe. (B) An increase in Laurdan dye fluorescence, indicating plasma membrane hydrophobicity due to higher lipid packing order, was observed in astrocytes treated with low pH media. (C,D) Force Microscopy (AFM) measurements show a significant rise in total surface force and surface/cortical stiffness (Young's Modulus) in astrocytes treated with low pH media. (E) FRET probe‐based force measurement shows an increase in FRET efficiency in astrocytes treated with low pH media, indicating a rise in plasma membrane lateral compression at lower pH units. NHA1 (SVG) is a passagable human astrocyte cell line, NHA2 (Lonza) is human primary astrocyte cells with limited passagibility, and mouse brain astrocytes are freshly isolated from the mouse cortex. All astrocyte sources showed similar trendlines in the assay at respective pH units. Also, see Figure S2 for representative FRET images associated with this panel. (F) Diagrammatic representation of the AFM measurements in the ‘upper panel’ shows the movement of the cantilever in attaching (indentation) position to the cell surface for force measurements. The force applied to the surface upon indentation and subsequent force applied by the cantilever to detach from the cell surface is designated as the ‘Total Detachment Force’. Diagrammatic representation of the AFM measurements in the ‘lower panel’ shows the parameters in detachment force measurements. The ‘force curve’ of AFM cantilever indentation to the cell surface is shown in blue and the ‘force curve’ of cantilever retraction/detachment is shown in red. Numerical labelling in the diagram describes the following: (1) The cantilever touches the cell and no force is detected, (2) the cantilever then presses the cell surface and an increase in force is registered until the cantilever reaches a pre‐set level, marked as 2, (3) After a set contact time, the cantilever is withdrawn from the cell surface until (4) the cantilever has completely separated from the cell surface. Fd is the maximal force needed to separate the cantilever from the cell surface, which includes the magnitude (marked as 6) and the number of times (marked as 5) of detachment events along the curve. Wd is the work done for detachment. (G) The analysed data shows a significant increase in total detachment force at lower pH units, demonstrating a rise in surface adhesiveness. (H) The panel shows a more resolved total detachment force dynamics: the left panel shows representative detachment force curves at physiological and low pH units; the right panel shows the distribution of detachment force values (normalised frequency) over 60 cells at each pH unit compared to pH 7.4. Overall, the data indicated that more astrocytes record enhanced detachment force at lower pH units versus pH 7.4, demonstrating an overall rise in astrocytes' surface adhesiveness. (I) SNA lectin staining of astrocytes' surface showed enhanced clustered organisation and spread density of surface glycans (sialic acid at α2,6 and α2,3 positions) at lower pH units. Figure S3 shows that the same cells also bear thick and long F‐actin stress emanating from cell surface at lower pH units. All datasets are reported as mean± SD. Significance is shown as *p < 0.05, **p < 0.01, ***p < 0.001; Mean is derived from three independent experiments. Image acquisition parameters for each channel were kept the same across each condition and over independent replicates. Recordings from at least 30–60 cells were taken for single‐cell measurements from each independent experiment

As the major cellular sensorium of the extracellular milieu, the plasma membrane should act as the first interface at which acid stress is sensed. Astrocytes in low pH microenvironments showed a significant increase in plasma membrane microviscosity, as demonstrated by DPH dye anisotropy (Figure 1A) [26]. In low pH microenvironments, reduced membrane fluidity was also substantiated by a corroborative increase in plasma membrane lipid packing order and hydrophobicity, as measured by Laurdan dye fluorescence (Figure 1B) [27, 28].

We further measured astrocyte total surface force and surface stiffness at low pHe versus physiological pHe, via Atomic Force Microscopy. Both total force and surface stiffness measurements confirmed a significant positive correlation between extracellular proton concentrations and rise in plasma membrane mechanical stress (Figure 1C,D).

We next wanted to determine whether differential mechanical stress generated due to different levels of pHe can be transmitted into the cell.

For this, we employed an F‐actin crosslinking alpha‐actinin FRET‐based mechanical stress sensor [22, 23, 24]. In this FRET‐based force‐sensing probe, one end of spectrin is fused with EGFP and another end with m‐Cherry. If the plasma membrane is laterally compressed via extensive protonation of its surface, there should be a gain in FRET efficiency as the distance between EGFP‐mCherry FRET pairs will decrease. This probe was transfected into astrocytes, and then an acidification protocol was followed. Astrocytes grown in high proton concentration microenvironments showed a higher gain in FRET probe efficiency (Figure 1E, also see Figures S2 and S3 for representative images related to this panel). This suggests that high extracellular proton levels caused in‐plane surface compression.

Importantly, in recovery experiments, when low pH conditions were replaced with medium at physiological pH, the FRET readings reversed to that observed at pH 7.4 (Figure 1E). This indicates that the FRET probe could rapidly and faithfully read the changes in extracellular pH levels due to its protonation‐dependent membrane compression and relaxation sensitivity.

The FRET‐based force measurements were made in two human astrocyte cells from different sources. NHA1/SVG is an SV40 transformed normal human astrocyte cell line engineered for better passaging [20], ease of handling and has been instrumental in many previous studies [29, 30, 31]. NHA2 represents untransformed human normal primary astrocytes (from Lonza). Besides these, we also compared freshly isolated astrocytes from postnatal mouse brains (Figure 1E). For subsequent *in vitro* studies, we used NHA1/SVG human astrocyte cell line. However, the major conclusions were confirmed in mouse primary astrocytes and in the mouse brain acidification model. Astrocytes from all sources showed similar trends in FRET efficiency at comparable pH levels. The next step was to deduce how low pHe translates into membrane lateral compressive stress, enabling astrocytes to adapt to acidosis.

### Low pHe is sensed by sialic acid containing glycan headgroups on the astrocyte surface enabling membrane compression

In the Atomic Force Microscopy (AFM) recordings, the cantilever detachment force required to detach the ‘cantilever’ from the cell's surface showed a positive correlation with the rise in extracellular proton concentrations (Figure 1F,G). The detachment force frequency distribution also indicated that more cells exerted a higher back pulling force on the AFM cantilever at low pHe values (Figure 1H, please see the associated diagram in panel ‘F’ for more details). This suggests that the surface might have developed a ‘dense glycocalyx’ due to protonation mediated glycan‐glycan ligations of membrane proteins and lipids. This phenomenon might have been responsible for exerting a back pulling force on the retracting cantilever due to glycocalyces ‘viscous adhesive nature’. We confirmed this speculation via SNA lectin staining (Figure 1I), which showed aggregation of predominant glycan moieties on the surface. These observations, therefore, indicate that protonation can physically drag the surface glycans into localised domains, ensuing membrane lateral compressive stress.

**FIGURE 2:**
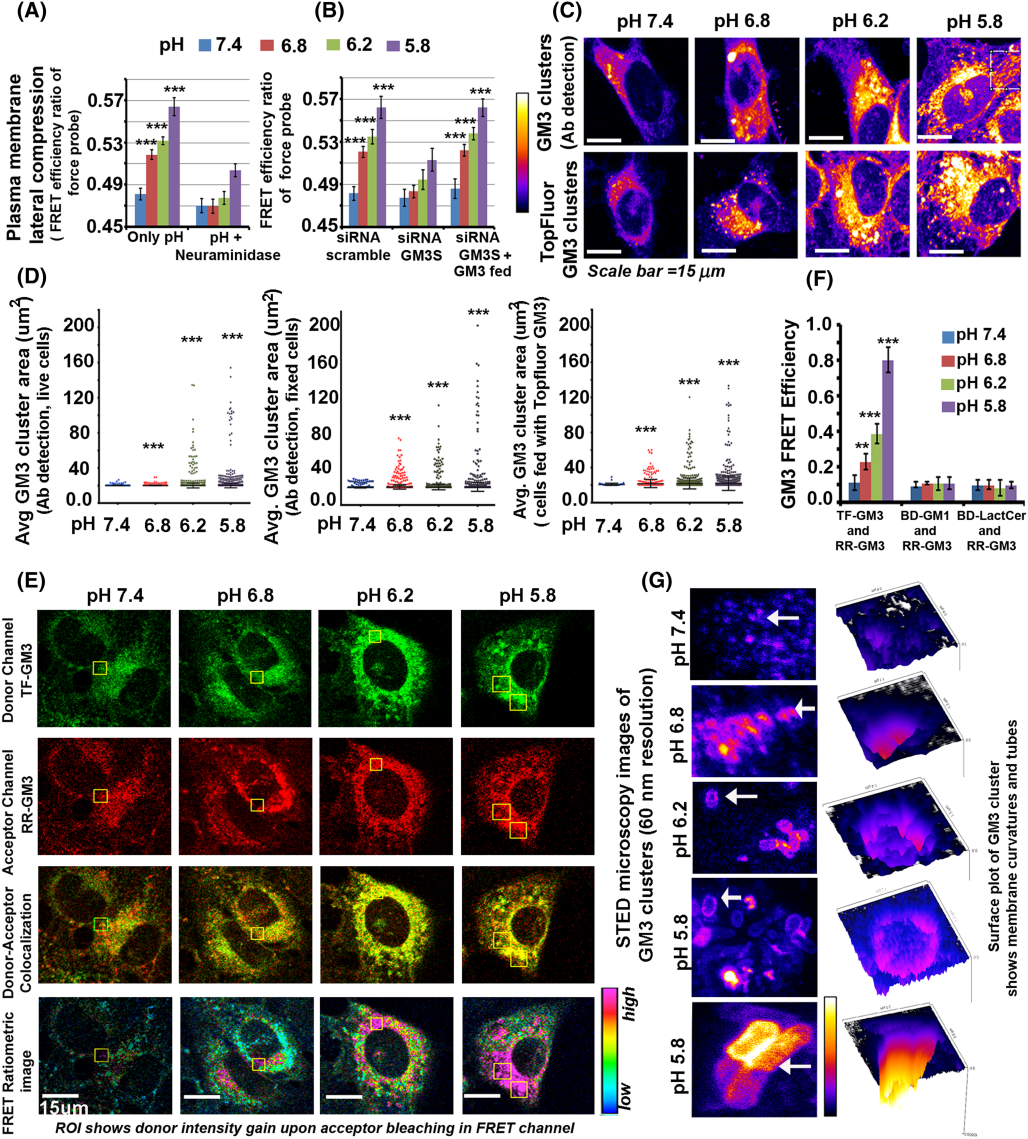
Sialic acid glycan headgroup containing GM3 glycosphingolipid is a crucial plasma membrane mechanical sensor of low pHe. (A) FRET‐based force probe could not efficiently sense low extracellular pH microenvironment upon treating astrocytes with neuraminidase versus the untreated cells. See Figure S4 for representative FRET images associated with this figure panel. (B) In low pH conditions, GM3 depleted astrocytes produced similar results as those seen in neuraminidase treatment. However, upon exogenous GM3 lipid feeding, the force sensing FRET probe efficiency to sense pHe was recovered. See Figure S5 for representative FRET images associated with this figure panel. (C) The top panel shows GM3 clusters on the astrocyte surface at different pHe, detected through anti‐GM3 antibody. The bottom panel shows surface GM3 clusters formed by fluorescently labelled GM3 lipid, which was fed to astrocytes and subsequently, pHe treatments were given. (D) Astrocytes' surface GM3 cluster area analysis at different pHe was performed, wherein GM3 clusters were detected with anti‐GM3 antibody and with plasma membrane fed fluorescent GM3 lipid. (E,F) An equimolar mixture of TopFluor tagged GM3 (TF‐GM3) and Rhodamine Red‐GM3 (RR‐GM3) was fed to astrocytes. Representative images of TopFluor GM3‐Rhodamine red GM3 FRET experiment in astrocytes at physiological and lower pH units is shown graphically in (F). Notice enhanced FRET ratio (in ROI) and colocalization of TopFluor GM3 (green) and Rhodamine Red GM3 (red) at low pH values, indicating co‐clustering/homo‐ligations. The graphical plot shows the high FRET efficiency of the force probe at low pH values, demonstrating GM3 homo‐clustering. No significant FRET was observed between similar FRET pairs when GM3‐Lactosylceramide (Bodipy‐LactCer and Rhodamine Red‐GM3) or GM3‐GM1 (Bodipy‐GM1 and Rhodamine Red‐GM3) equimolar lipid mixtures were fed to astrocytes (see Figure S6 for representative FRET images associated with this figure panel). (G) ROI of astrocyte surface, imaged through STED super‐resolution microscopy (at 60‐ to 50‐nm resolution), revealed GM3 clustered areas as membrane invaginations/curvatures and tubes at low pH values. White arrows indicate topologies in each image for which surface plot is generated in Fiji software. This indicates the transformation of flat to conical and more curved and tube‐like GM3 enriched structures, on astrocyte surface, with a drop in pHe values. All datasets are reported as mean± SD. Significance is shown as *p < 0.05, **p < 0.01, ***p < 0.001. Mean is derived from three independent experiments. Recordings from at least 30 cells are taken for single‐cell measurements from each independent experiment. Image acquisition parameters for each channel were kept the same across each condition and over independent replicates

Notably, within glycan headgroups of both lipids and proteins, the negatively charged sialic acid moiety is highly susceptible to protonation. So, the next question that arose from SNA lectin based observation was whether sialic acid is involved in glycan clustering and lateral compressive stress? To address this, astrocytes transfected with the FRET‐based force‐sensing plasmid were treated with neuraminidase/sialidase to shave the sialic acid moieties, followed by pH treatment. A significant loss in FRET efficiency of the force‐sensing probe in astrocytes exposed to low pHe values was observed. This indicates that cells had lost their ability to sense and transmit the information on high microenvironmental proton levels (Figure 2A, see Figure S4 for representative FRET efficiency images). We thus found that the sialic acid moieties play a significant role in generating pH‐mediated surface mechanical stress. It may be noted here that neuraminidase activity is reduced at a pH below 6.0 [32]. Therefore, the membrane compression at pH 5.8 was slightly higher than at other pH ranges (Figure 2A).

Overall, these observations indicate that sialic acid‐containing conformationally flexible surface molecules can play an important role in extracellular pH sensing.

### GM3 glycosphingolipid is crucial in sensing low pHe via protonation dependent differential clustering of its sialic acid glycan moiety

Kidney and gastric luminal epithelia, constantly exposed to low pH, are enriched in GM3 glycosphingolipid at their apical membranes [33, 34, 35]. Therefore, we wondered whether astrocytes in an acidified microenvironment (such as that encountered during ischaemia or cancer) could utilise GM3 as a major surface pH sensor due to its conformational receptivity and susceptibility to protonation via its sialic acid containing headgroup.

To explore this, we depleted GM3 below homeostatic levels via siRNA mediated downregulation of the enzyme ST3Gal5 (GM3S). An siRNA concentration that downregulated GM3S to 60%–70% was selected, as complete depletion of this gene leads to aberrant cell de‐adhesion [36]. We expected that if GM3 per se is required for pHe sensing, reducing its levels (through GM3S siRNA) would be sufficient to disturb the protonation‐driven clustered organisation involved in the pH sensing process.

GM3 depleted astrocytes were subjected to various pH microenvironments, and FRET‐based mechanical stress probe readings were inferred (Figure 2B). Reduced surface GM3 caused significant loss in the compression of the FRET probe. Hence, it failed to correctly sense the low extracellular pH levels (Figures 2B and S5). This suggests that GM3 is a crucial pHe sensor on the surface. Complimentary studies were performed by exogenously feeding GM3 to GM3 depleted astrocytes before pH treatment, and this preserved FRET probe sensitivity to pHe levels (Figure 2B).

### Differential GM3 clustering triggers ER associated IRE1‐sXBP1 survival genes at low pHe

After obtaining evidence of the involvement of GM3 in surface pH sensing and in the translation of this information into membrane stress, we looked for direct evidence of differential GM3 clusters on astrocytes in response to different concentrations of extracellular protons.

For this, we detected the GM3 clusters by two imaging‐based protocols. In the first method, the anti‐GM3 antibody was incubated on physiological and low pHe treated live cells (Figure 2C,D, cells were kept on ice during surface incubation of anti‐GM3 antibody to prevent endocytosis). Another set of cells were given pHe treatments and were first fixed at RT followed by surface incubation of antibody (without permeabilization) (Figure 2D). The signal was developed by incubating the fluorophore‐conjugated secondary antibody on the cell surface without cell permeabilization to reveal surface levels and organisation of GM3 (Figure 2C,D). In the second method, the astrocyte plasma membrane was first fed with fluorescently conjugated GM3 lipid (TopFluor GM3), followed by pH treatments (Figure 2C,D). Both methods of GM3 cluster detection showed similar results at comparable pHs. Indeed, at lower pH values, we noticed a steep rise in the GM3 cluster area.

To further confirm the GM3‐GM3 homo‐clustered organisation, equimolar concentrations of FRET pair labelled GM3 lipids, that is, TopFlour GM3 (TF‐GM3) and Rhodamine Red GM3 (RR‐GM3), were fed to astrocytes surface, followed by pH treatments (Figure 2E,F). In this assay, we noticed a significant gain in FRET efficiency between FRET donor‐acceptor pair labelled GM3 molecules at low pH values, showing homo‐ligation of GM3 molecules and formation of higher‐order oligomeric‐multimeric states (Figure 2E).

It is of utmost importance to note here that we did not register any significant FRET between lactosylceramide and GM3 or between GM1 and GM3, when labelled likewise with FRET donor‐acceptor pairs (Figures 2E and S6). Lactosylceramide has a similar structure to GM3 except that it lacks sialic acid moiety. This again suggests that GM3 interacts with neighbouring GM3 molecules through the sialic acid protonation at low pH. GM1 is structurally similar to GM3 and also contains a sialic acid moiety, but the sialic acid is not situated at the terminal moiety of the sugar headgroup on this lipid, unlike that in GM3. This suggests that the position of sialic acid is also a strong determinant of GM3 homo‐ligation in response to protonation. These observations that the presence and the position of sialic acid imparts unique properties to GM3 to respond to protonation and act as a pH sensor is supported by the observation that both lactosylceramide and GM1 failed to produce micron‐sized clusters akin to GM3 in low pH conditions (see respective panels in Figure S6).

We wanted to explore whether GM3 clustering induced membrane compression can directly transmit information on pHe levels to the astrocyte interior to mount an adaptive response. We tried to resolve the GM3 clustered topology at the nanoscale via STED super‐resolution microscopy and found that GM3 clustered areas, at low pHe, form inwardly directed membrane curvatures and tubes (Figure 2G).

**FIGURE 3:**
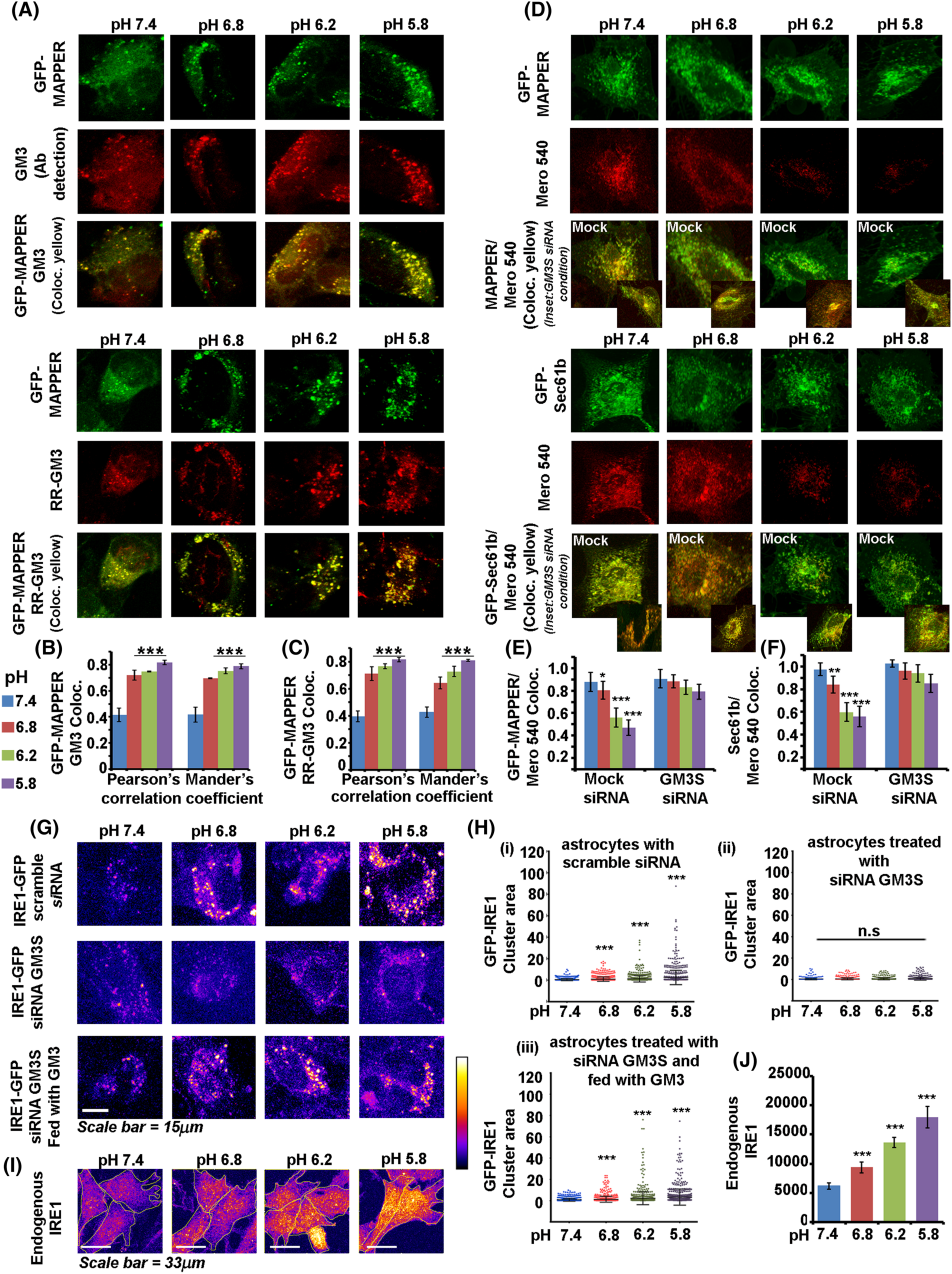
GM3 glycosphingolipid mechanotransduce microenvironmental acidosis information to the endoplasmic reticulum triggering IRE1 mediated cytoprotection. (A–C) Confocal microscopy images show GM3 clusters colocalized with peripheral ER‐PM contact site model protein GFP‐MAPPER at low pH values. Rhodamine Red labelled GM3 (RR‐GM3) lipid fed to astrocytes also showed similar colocalization with GFP‐MAPPER. Please also see Figure S7. (D–F) Colocalization of merocyanine 540 with peripheral ER marker (GFP‐MAPPER) and total ER marker (Sec61b, also found in nuclear envelope membrane) were significantly reduced in astrocytes exposed to low pH, indicating an increase in stiffness of ER membranes and nuclear envelope. Insets show merocyanine 540 colocalizations with ER markers in GM3S siRNA condition in different pH treatments. Enhancement of colocalization in GM3S siRNA transfected cells vs scramble siRNA transfected cells in respective pH conditions indicated more fluidic ER membranes. Mander's colocalization coefficient is shown in the graphs. (G,H) Doxycycline inducible (10 nM), EGFP tagged IRE1 transfected astrocytes showed significant IRE1 clustering (oligomerization) at low pHe values, whereas this potency was lost in astrocytes depleted of GM3 (GM3S siRNA). However, the clustering was preserved in GM3 depleted astrocytes that were exogenously fed with GM3 lipid. (I,J) Detection of an endogenous pool of activated IRE1 via phospho‐IRE1 antibody also confirmed IRE1 clustering at low pHe values. All datasets are reported as mean ± SD. Significance is shown as *p < 0.05, **p < 0.01, ***p < 0.001; NS means non‐ significant. Mean is derived from three independent experiments. Recordings from at least 30–60 cells are taken for single‐cell measurements from each independent experiment. Image acquisition parameters for each channel were kept the same across each condition and over independent replicates

Such GM3 curvatures can contact the plasma membrane juxtaposed peripheral ER and influence ER stiffness as well as ER‐associated survival dynamics. Therefore, we wanted to decipher whether GM3 curvatures‐ER contact sites enabled pHe mechanotransduction. To trace this, we transfected the astrocytes with a plasmid construct encoding the peripheral ER membrane fluorescent probe (named GFP‐MAPPER), which enables direct detection of ER‐PM contact‐sites [37]. We found that the GM3 clustered organisation at low pH showed significant colocalization with GFP‐MAPPER (Figure 3A–C). In fact, not just with STED, even in maximum intensity projections of confocal image stacks of 0.1 μm slice thickness, we could detect prominent GM3 curved and tube‐like assemblies that significantly colocalized with GFP‐MAPPER at low pH (Figure S7A). We obtained similar results with fluorescently labelled GM3 lipid fed to plasma membranes (Figure S7B), which remarkably colocalized with another peripheral ER marker E‐Syt2 tagged with mCherry [38]. This indicates that clustered GM3 focal curvatures might have fused with peripheral ER. If GM3 enriched inward curvatures and tubes crosstalk with peripheral ER membranes, these stiff membrane areas may directly transmit the low pHe mediated compressive force to the ER membranes and even to the nucleus and chromatin (as the nuclear envelope is continuous with the ER membrane) [39]. To test this, we performed live‐cell spectral measurements of merocyanine 540 fluorescence emission to map the fluidity of (i) ER‐PM contact points (using GFP‐MAPPER to identify peripheral ER) (ii) and of total ER (using GFP‐Sec61beta).

Merocyanine 540 dye is partitioned out from high lipid packed areas of the bilayer, and reduction in its fluorescent emission represents more lipid packed/or stiff membranes [40, 41]. Colocalization of merocyanine 540 with peripheral and total ER markers was significantly reduced in astrocytes exposed to low pHe (Figure 3D–F). Hence, in astrocytes interfacing low pH, ER membranes were more densely lipid packed and stiffer. Conversely, in GM3 depleted astrocytes exposed to low pH, merocyanine 540 was well colocalized with ER membranes, clearly indicating that GM3 was responsible for ER membrane stiffening (please see insets in Figure 3D–F).

In keeping with the fact that the ER is continuous with the nuclear envelope, we also found an increase in nuclear envelope stiffness indicator Lamin A [42] but lack of DNA damage indicator phospho‐γH2AX. This protective stiffening effect on chromatin was significantly lost in GM3 depleted astrocytes exposed to low pHe's (Figures S8–S11). This suggests that GM3 clustering passes on the lipid bilayer stress to both ER and nuclear envelope (NE) membranes leading to their rigidification.

For the nucleus, stiffening of the envelope would mean robustly protecting the chromatin from DNA damage [43]. Further, since chromatin has direct contact sites with the nuclear envelope and that euchromatin is more proximal to the nuclear envelope, NE stiffening should aid in making the chromatin conformationally poised for binding of appropriate mechanotransduced transcription factors for induction of pHe responsive genes. Indeed, we found an increase in nuclear H3K9ac and H3K27ac [44, 45], which are crucially involved in positively regulating gene transcription (Figure S12). In GM3 depleted astrocytes, H3K9ac and H3K27ac were not found to increase upon low pH treatments. However, GM3 depleted astrocytes fed with GM3 lipid prior to treatment showed a significant rise in H3K9ac and H3K27ac levels, suggesting that GM3 was involved in enabling sensing and transduction of extracellular low pH information to the nucleus for activation of transcription.

However, in the context of the ER, could GM3 mediated mechanical remodelling/stiffening mean appropriately switching on the crucial cytoprotective lipid biosynthesis and UPR machinery via the ER stress sensor IRE1 [46, 47]? ER‐resident IRE1 has been shown to sense membrane lipid stress via its self‐oligomerization, phosphorylation and activation to effect generation of cytoprotective pathways [48, 49]. Activation‐induced enhancement in IRE1 RNAase activity generates splicing of the IRE1 associated factor XBP1. Spliced XBP1 (sXBP1) transcribes SREBP2, which upregulates ACSS2, a crucial enzyme in cholesterol synthesis [50]. So, the next step was to determine whether IRE1 and its downstream effectors act as essential mediators in pHe mechano‐adaptations via GM3.To test this, we transfected astrocytes with a doxycycline‐inducible conditional plasmid of IRE1 fused with EGFP reporter gene [48]. We induced the plasmid with a very low dose of doxycycline and then incubated the cells with media adjusted to various pH values. We found that IRE1 was significantly clustered in low pH incubations, and this clustering was substantially less in GM3 depleted cells in the respective pH conditions. In contrast, in GM3 depleted cells exogenously fed with GM3 lipid prior to low pH treatments, IRE1 clustering was found to be preserved (Figure 3G,H).

**FIGURE 4:**
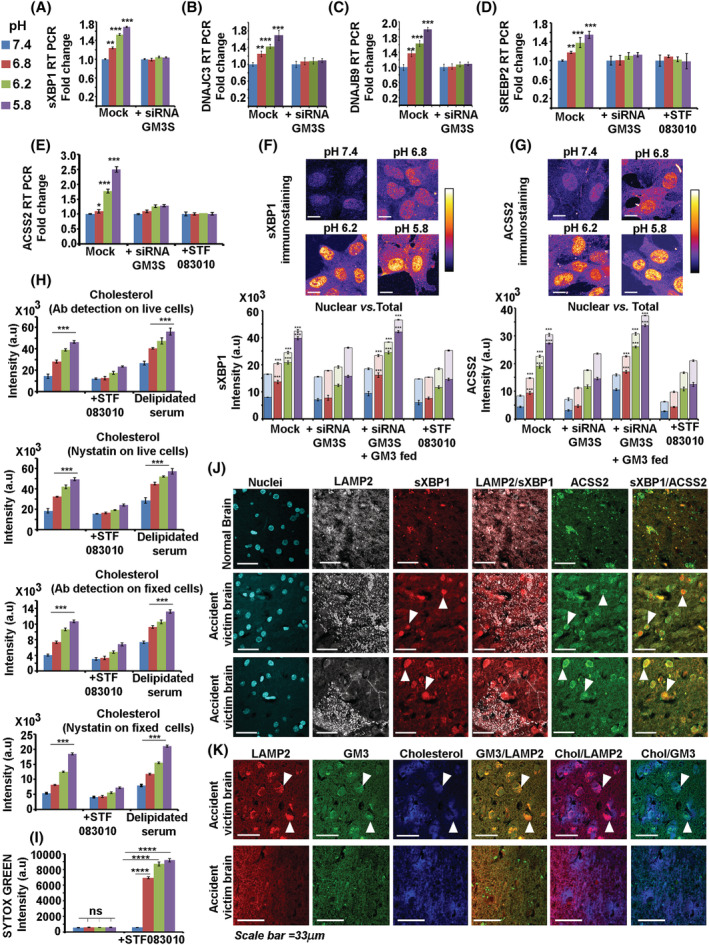
Activated IRE1 triggers the intracellular sXBP1‐SREBP2‐ACSS2‐cholesterol survival circuit in response to microenvironmental acidosis. (A) A significant increase in sXBP1 transcript formed through the RNAase splicing activity of IRE1 was observed in astrocytes incubated with low pH media. This effect was abolished in GM3S siRNA transfected astrocytes. Mock refers to scrambled siRNA transfections. (B–D) A significant increase in sXBP1 cytoprotective targets DNAJC3, DNAJB9 and SREBP2 was observed in astrocytes incubated with low pH media. However, this was not observed in astrocytes that were depleted of GM3. SREBP2 transcription was further confirmed to be affected in astrocytes treated with STF‐083010 (60 μM) that inhibits sXBP1 generation. (E) SREBP2 downstream cytoprotective target, ACSS2, transcription enhancement was observed in astrocytes incubated with low pH media. However, this was not observed in astrocytes that were depleted of GM3 or of sXBP1 (via STF‐083010). (F,G) A significant increase in nuclear translocation of sXBP1 and ACSS2 is observed at low pHe values. This was not observed in astrocytes depleted of GM3 or sXBP1 (via STF‐083010). However, the effect was preserved in astrocytes depleted of GM3 synthase but was externally fed with GM3 lipid. (H) A significant rise in surface cholesterol was observed in low pH conditions, while this was not so in sXBP1 inhibited conditions. Astrocytes grown in delipidated medium, deprived of an external source of cholesterol, also showed a rise in surface cholesterol in low pH conditions, indicating an endogenous source of cholesterol biosynthesis. See Figure S16 for representative images. (I) Sytox green dye enhanced cell permeability was observed in the presence of sXBP1 inhibitor (STF‐083010) at low pH values, indicating a crucial role of sXBP1 in maintaining plasma membrane integrity. (J,K) Ischaemic brains of road accident victims showed higher nuclear sXBP1‐ACSS2 in acidified regions, wherein acidified regions are identified by high LAMP2 expression versus normal regions with low LAMP2 expression. LAMP2 enriched regions also showed enrichment and enhanced colocalization of GM3 with cholesterol. Ischaemic brain sections were from lane H with positions H6, H7, H8, H9 of US Biomax tissue array, cat no.GL806e (B031). All datasets are reported as mean ± SD. Significance is shown as *p < 0.05, **p < 0.01, ***p < 0.001; Mean is derived from three independent experiments. Recordings from at least 60 cells were taken for single‐cell measurements from each independent experiment. Image acquisition parameters were kept the same in each condition and over independent replicates

IRE1 homo‐clustering causes its phosphorylation‐dependent activation; hence the observed fluorescent IRE1 clusters essentially represent activated IRE1. Still, we confirmed endogenous IRE1 phosphorylation at low pH values in GM3 undepleted, GM3 depleted, and GM3 repleted cells via the use of a specific antibody (Figure 3I,J). IRE1 oligomeric activated state was positively correlated with the generation of spliced XBP1 in low pHe values (Figure 4A). sXBP1 levels were found to be significantly lower in GM3 depleted cells exposed to low pH. This supports the observations that the GM3 clustered organisation passed the plasma membrane stress to ER membranes via contact and fusion, causing ER lipid stress, which is reported to activate IRE1 activation, leading to the formation of sXBP1 (Figure 4A).

### Cholesterol is a major biosynthetic product of GM3 activated IRE1‐sXBP1 mechanotransduction enabling survival response machinery at low pHe

We observed that in low pH, sXBP1 cytoprotective targets such as DNAJC3 (p85IPK), DNAJB9 and SREBP2, were upregulated in astrocytes (Figure 4A–D). SREBP2 generation was significantly less in sXBP1 inhibited (via STF‐083010 treatment), and GM3 depleted astrocytes when exposed to low pHe values (Figure 4D).

Since SREBP2 downstream target ACSS2 generates excess Acetyl CoA, which serves as a critical substrate for cholesterol synthesis, we further explored the link between ACSS2 and the GM3‐IRE1‐sXBP1 axis. We observed a significant correlative rise in sXBP1 and ACSS2 expression and their nuclear translocation at low pHe values, which was found to be inhibited in STF‐083010 treated and GM3 depleted cells, while this was not so when GM3 depleted cells were fed with exogenous GM3 before pH treatments (Figure 4E–G). This suggests that GM3 clustered organisation was crucially involved in ACSS2 mediated cholesterol biosynthesis via the IRE1‐sXBP1‐SREBP2 arm. The role of GM3 was confirmed through immunocytochemistry in low pHe incubated astrocytes (Figure S13). We also established the indispensability of sXBP1 in the transcription of SREBP2‐ACSS2 in freshly isolated mouse primary astrocytes (Figure S14), which was comparable to human astrocytes (Figure S15). The sXBP1‐SREBP2‐ACSS2 lipogenesis axis correlated with the rise in surface cholesterol in low pH conditions, indicating excess cholesterol synthesis and surface transport. At the same time, this was not so in STF‐083010 treated condition, wherein sXBP1 is inhibited (Figures 4H and S16). We also tested whether the rise in surface cholesterol at low pH values was due to cholesterol extraction from the media in the form of lipoproteins or endogenous biosynthesis. For this, we repeated the surface cholesterol measurements in the delipidated media at different pHs. We found that the raised surface cholesterol was predominantly due to endogenous synthesis and not from the media (Figures 4H and S16). Intriguingly, in a de‐lipidated medium, the surface cholesterol was far more enhanced, again confirming the existence of endogenous cholesterol biosynthesis feedback mechanisms to regulate the levels of crucial lipids on the surface [51].

To confirm the cytoprotective role of sXBP1‐cholesterol in the prevention of low pHe mediated membrane acid hydrolysis and leakage, we undertook cell membrane permeability assays. sXBP1 generation inhibitor (STF‐083010) was used to suppress the excess cholesterol synthesis pathway and thus its surface levels. The enhanced permeability of membranes at low pH, upon reduction in surface cholesterol, confirmed the necessity to maintain high levels of surface cholesterol (Figure 4I).

However, the crucial question was: Is this phenomenon observed in acidified brain regions? To ascertain this, we first needed a reliable marker of acidification. Surface expression of LAMP2 protein enables the identification of acidified tissue regions [52]. *In vitro*, astrocytes showed high LAMP2 surface expression when exposed to low pHe (Figure S17). As identified by surface LAMP2 expression, ischaemic regions of the otherwise normal human brain showed a corroborative increase in nuclear sXBP1‐ACSS2 and GM3‐cholesterol colocalization. This observation supports GM3‐sXBP1‐ACSS2‐cholesterol axis cytoprotective function in low pH conditions (Figure 4J,K).

**FIGURE 5:**
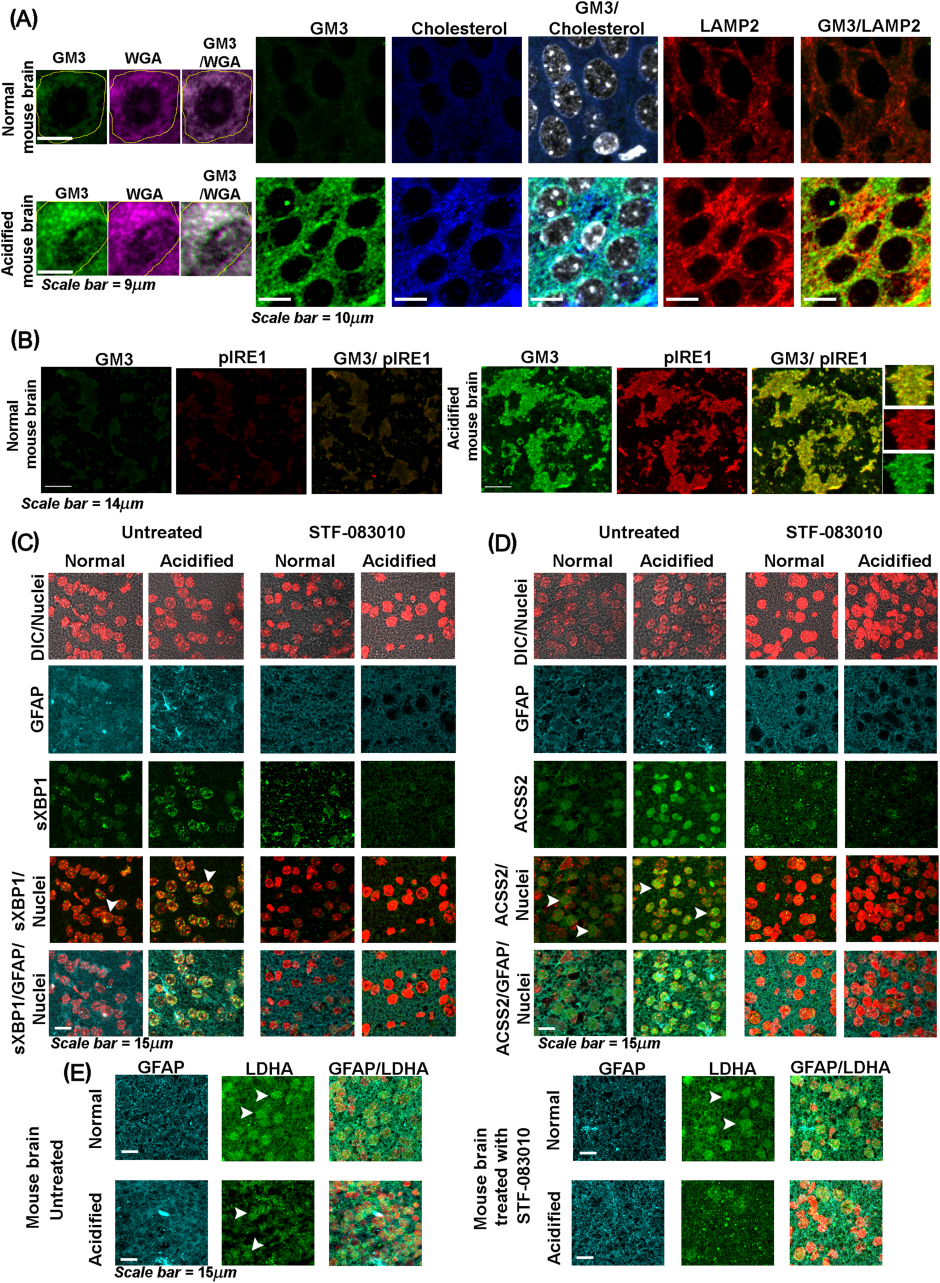
*In vivo* mouse model of brain acidification studies faithfully recapitulates the *in vitro* data on crucial involvement of GM3‐IRE1‐sXBP1‐ACSS2‐cholesterol survival mechano‐machinery in microenvironmental acidosis. Mouse brain acidification was generated by 7% CO_2_ inhalation for 2.5 h. See the methods section for more details. (A) Inset shows strong colocalization of clustered GM3 with WGA, a plasma membrane marker, indicating highly clustered GM3 on the surface in acidified vs non‐acidified brain astrocytes. Clustered organisation of surface GM3 showed significant colocalization with high cholesterol levels and LAMP2 (an acidosis marker). (B) Activated IRE‐1 (phospho‐IRE1) showed significant colocalization with GM3 clusters in the astrocytes from acidified brain vs normal brain. Also, see inset for clustered colocalization. (C) sXBP1 showed significant nuclear translocation in the astrocytes from acidified brain versus normal brain, which was inhibited in STF‐083010 injected acidified brains. GFAP is an astrocyte marker. (D) ACSS2 showed significant nuclear translocation in the astrocytes from acidified brain versus normal brain, which was inhibited in STF‐083010 injected acidified brains. GFAP is an astrocyte marker. (E) STF‐083010 injected acidified brains showed higher LDHA release in the extracellular spaces, identified by punctate dots versus normal smooth cytoplasmic staining, indicating enhanced cell membrane permeability and necrosis

To further substantiate that this axis works faithfully *in vivo*, we induced brain acidification in postnatal mice by established protocols and compared it with un‐acidified brains. The brain acidification was confirmed by CAIX and LAMP2 immunostaining (Figures S18 and 5A). Surface LAMP2 positive regions showed corroborative GM3 clustering and high cholesterol levels (Figure 5A). Regions in astrocytes with clustered surface GM3 correlated well with activation of IRE1 (Figure 5B) and showed enhanced nuclear translocation of sXBP1 (Figure 5C, white arrows points to nuclear localisation). The sXBP1 nuclear translocation in the acidified brain was substantially reduced when treated with the inhibitor of sXBP1 generation, STF‐083010. The astrocytes in the acidified brain also showed significantly higher levels of SREBP2 (Figure S19) and ACSS2 (Figure 5D). However, this was not observed in brains treated with STF‐083010. sXBP1 inhibition in mouse primary astrocytes and in the acidified mouse brain via STF‐083010 treatment showed downregulation of sXBP1 pro‐survival targets such as DNAJC3, GRP78 and instead enhanced ER stress anti‐survival proteins such as ATF4 and NF‐kB (Figure S20–S23) [53, 54, 55]. STF‐083010 treated acidified mouse brain showed high LDHA levels in the extracellular spaces (see extracellular punctate dots vs smooth cytoplasmic staining), indicating enhanced cell membrane damage and necrosis (Figure 5E).

Together, these observations support the consistency of *in vitro* and *in vivo* pHe sensing and adaptation machinery.

### Inhibition of splicing activity of IRE1 in combination with depletion of excess surface cholesterol negatively impacts the growth of astrocytic tumours

**FIGURE 6:**
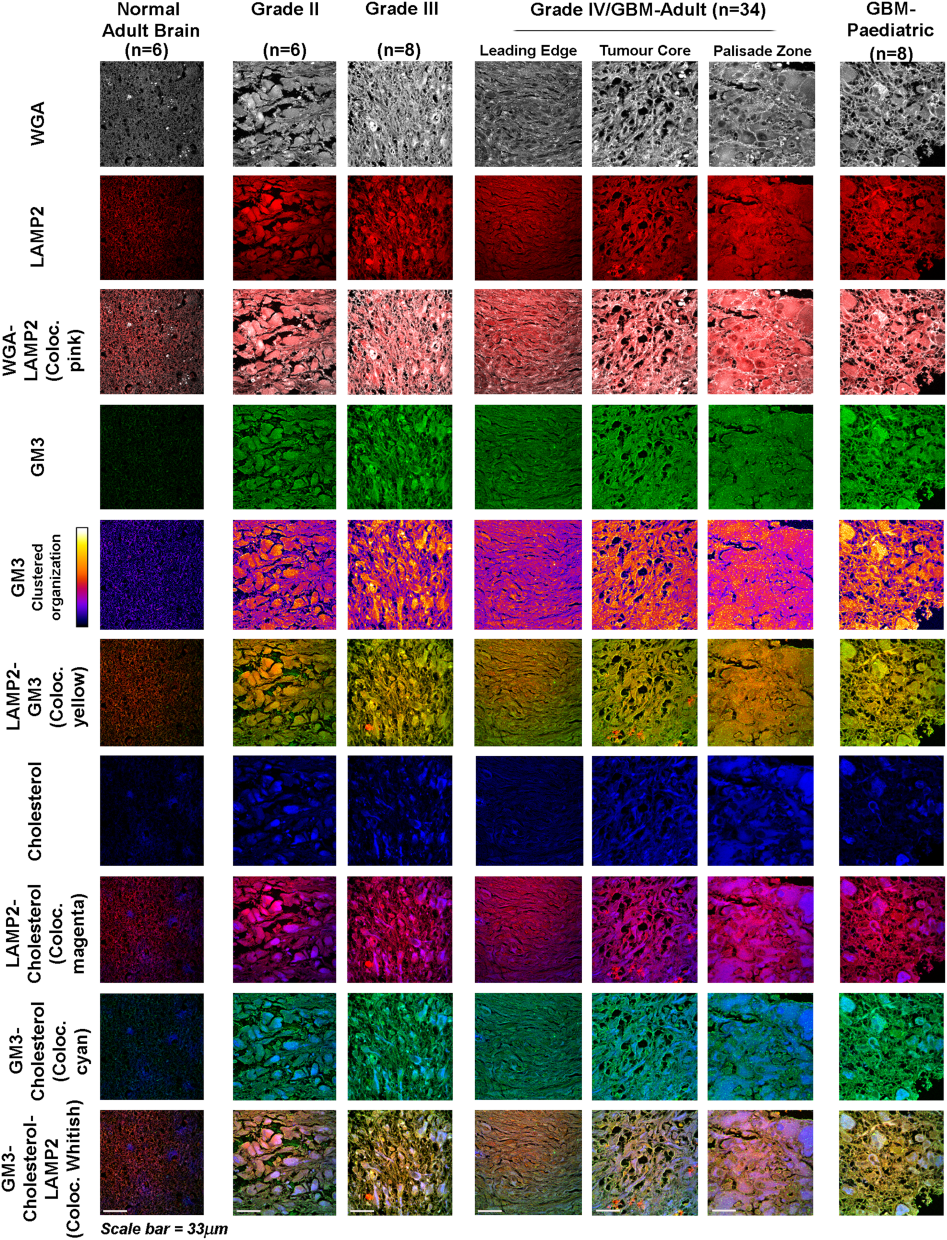
Acidic regions in astrocytic tumours of different grades show a correlated increase in GM3‐sXBP1‐ACSS2‐cholesterol survival machinery. Human astrocyte associated brain tumours of different grades showed corresponding increases in surface GM3, GM3 clustered organisation (shown as fire LUT pseudo‐colour intensity images) and surface cholesterol in acidified regions, identified through LAMP2 expression colocalisation. WGA lectin was used to label the cell surface. Interestingly, the GM3‐cholesterol expression axis shows therapeutic relevance in all grades and also in GBM/Grade IV of paediatric origin. The numbers of patient tissue samples examined are indicated in the figure panels

**FIGURE 7:**
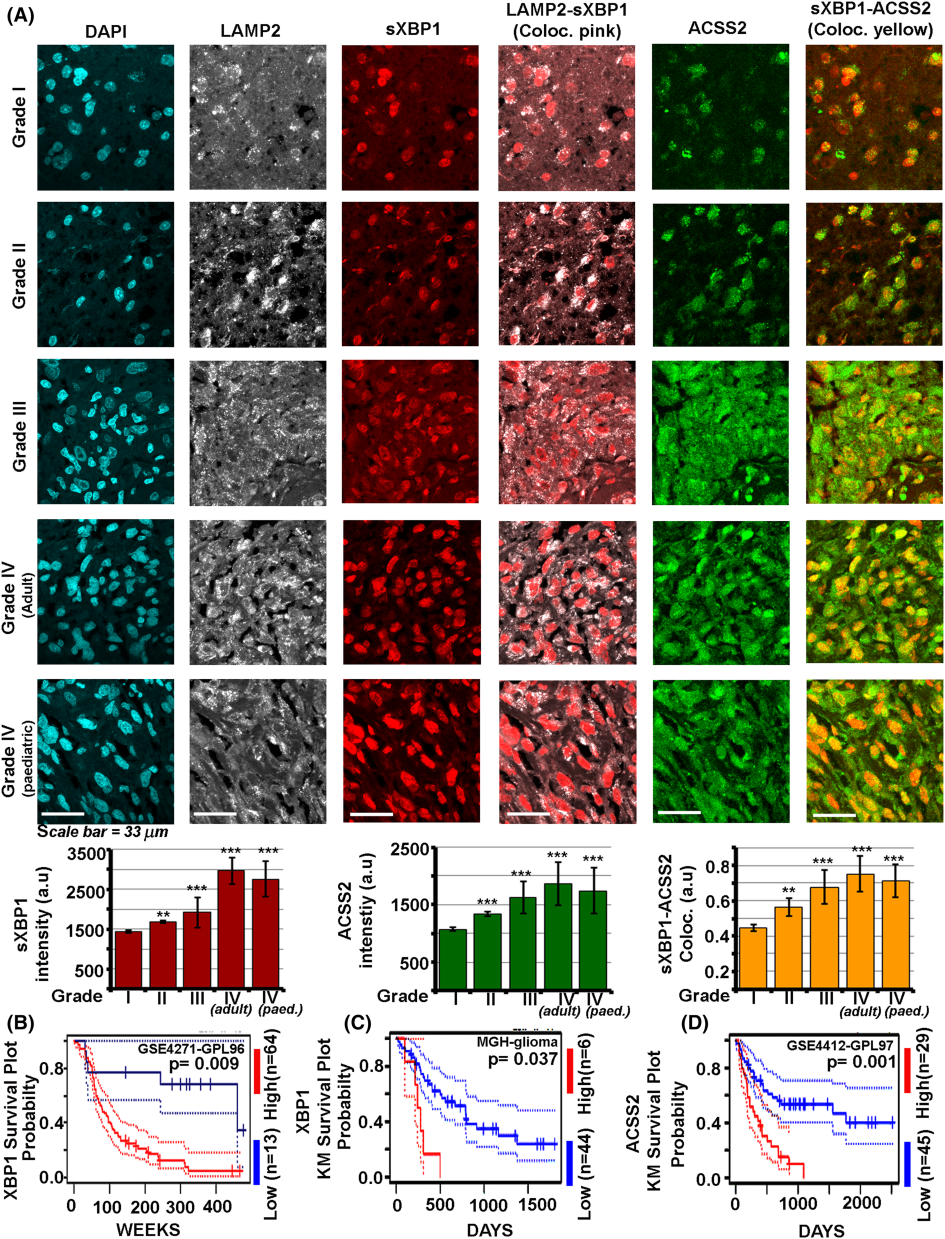
Acidic regions in astrocytic tumours of different grades show a correlated increase in the nuclear sXBP1‐ACSS2 survival axis. (A) Human astrocyte associated brain tumours of different grades (I‐IV) showed a corresponding increase in expression and colocalization of nuclear sXBP1 with its target ACSS2 in acidified regions (identified through LAMP2 expression in the same regions). Interestingly, the sXBP1‐ACSS2 expression axis indicates therapeutic relevance in all grades and also in GBM/Grade IV of paediatric origin. (B,C) The Kaplan Meier survival and prognostic value analysis from 2 different dataset sources indicate overall reduced survival of astrocytoma/GBM patients in which XBP1 expression was significantly high. (D) Likewise, high expression of ACSS2 in astrocytoma/GBM tissues was associated with reduced patient survival. Data is extracted from the PrognoScan database. High and Low refers to expression levels in brain tumour patients. No. of patients in each category and p values are indicated in the figure. Please see Supplementary Methods for more details

There is strong evidence that acid stress promotes tumourigenesis. Hence, we wondered whether brain tumours of astrocytic origin recapitulate the same GM3‐IRE1‐sXBP1‐SREBP2‐ACSS2‐cholesterol survival axis as deduced in astrocytes under acid stress. We first examined astrocytoma/glioblastoma patient tissues immuno‐histochemically for presence of anti‐acid molecular players, which were identified earlier in our study. Since LAMP2 protein is observed to be trafficked to the surface of GBM cells in low pHe (Figure S24), LAMP2 antibody was used to detect the acidic regions in GBM tissue. There was significant co‐occurrence and enrichment of clustered GM3 and high cholesterol in acidified regions of astrocytic tumours vs the normal human brain (Figure 6). We further documented that astrocytic tumours of different grades showed a corresponding increase in nuclear sXBP1 and ACSS2 localisation in acidified regions, indicating the preservation of GM3‐sXBP1‐ACSS2‐Cholesterol survival machinery in acidified tumour zones (Figure 7A). Also, the survival and the prognostic value of XBP1 and ACSS2 in patients with astrocytic tumours were confirmed to be highly significant, wherein higher expression was associated with reduced survival (Figure 7A–D).

**FIGURE 8:**
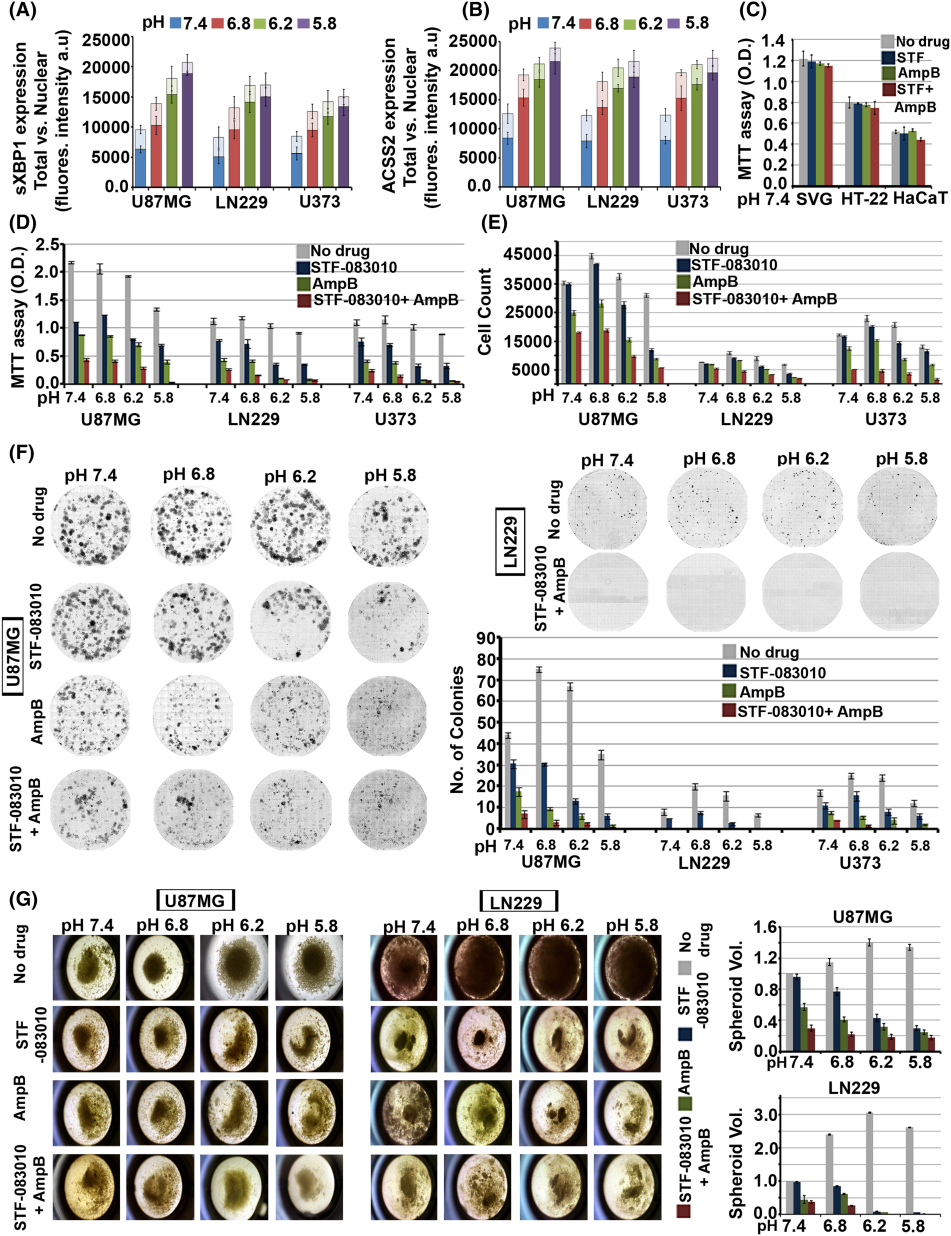
Suppression of GM3‐IRE1‐sXBP1‐ACSS2‐cholesterol mechano‐machinery is efficient in anti‐astrocytoma/GBM therapy. (A,B) GBM patient‐derived cell lines (U87MG, LN229 and U373) showed a significant increase in nuclear translocation of sXBP1 and ACSS2 in low pH conditions. Also, see Figure S25 for representative images. (C,D) Lower optical density (OD) values in MTT assay indicate cell toxicity. Low ODs were registered in GBM cells treated with STF‐083010 (60 μM), Amphotericin B (10 μM) and STF‐083010 + Amphotericin B drug combination at different pHe. Panel C shows that these drug treatments did not affect normal cells (SVG‐astrocytes; HT‐22‐ neurons, HaCaT‐keratinocytes) at physiological pH. (E) GBM cells treated with STF‐083010 (60 μM), Amphotericin B (10 μM) and STF‐083010 + Amphotericin B showed significantly lower cell counts, indicating loss of cell viability. (F) GBM tumour cell colonies treated with STF‐083010 (60 μM), Amphotericin B (10 μM) and STF‐083010 + Amphotericin B showed a significant reduction in tumour cell colonies, generated through clonal expansion, indicating loss of self‐renewability. (G) GBM cells treated with STF‐083010 (60 μM), Amphotericin B (10 μM) and STF‐083010 + Amphotericin B showed significant disruption in spheroid maintenance and growth after 5 days of continuous treatments. Notice significant disintegration and rupture of spheroids in drug treatments at different pH units. Mean is derived from three independent experiments. In single‐cell measurements, recordings are made from at least 60 cells from each independent experiment of total *N* = 3

If excess cholesterol generated from the GM3‐IRE1‐sXBP1‐ACSS2 axis is essential for the survival response in the acidified tumour environment, then would it be beneficial to deplete the excessively raised levels of cholesterol in tumours [56]? Therefore, we wanted to test whether simultaneous inhibition of sXBP1 mediated excess cholesterol synthesis pathway along with its surface depletion may prove to be effective in attenuating tumour growth at all pHs. The GBM patient‐derived cell lines indeed showed increased levels and nuclear translocation of sXBP1 and ACSS2 in low pH conditions, making it amenable to test our hypothesis (Figure 8A,B, for images, see Figure S25). For this, we used STF‐083010 [25] to inhibit the IRE1‐sXBPI‐SREBP2‐ACSS2‐cholesterol synthesis axis and combined it with Amphotericin B (cholesterol sequestering FDA approved antibiotic, used at non‐toxic concentrations as referenced in citation no [57]) to simultaneously deplete the excess surface cholesterol levels via cholesterol sequestration from the plasma membrane.

The effects of combination drugs on tumour cell survival were assayed in several GBM tumour cells through MTT, cell count, colony and sphere growth maintenance assays (Figure 8D–G). We observed that the cholesterol targeting drug combination was effective in attenuating tumour growth at various pHs. The formulation even worked well on tumour cells grown at physiological pH because tumour cells generally require higher cholesterol even at physiological pH than normal brain cells (Figure 8C).

**FIGURE 9:**
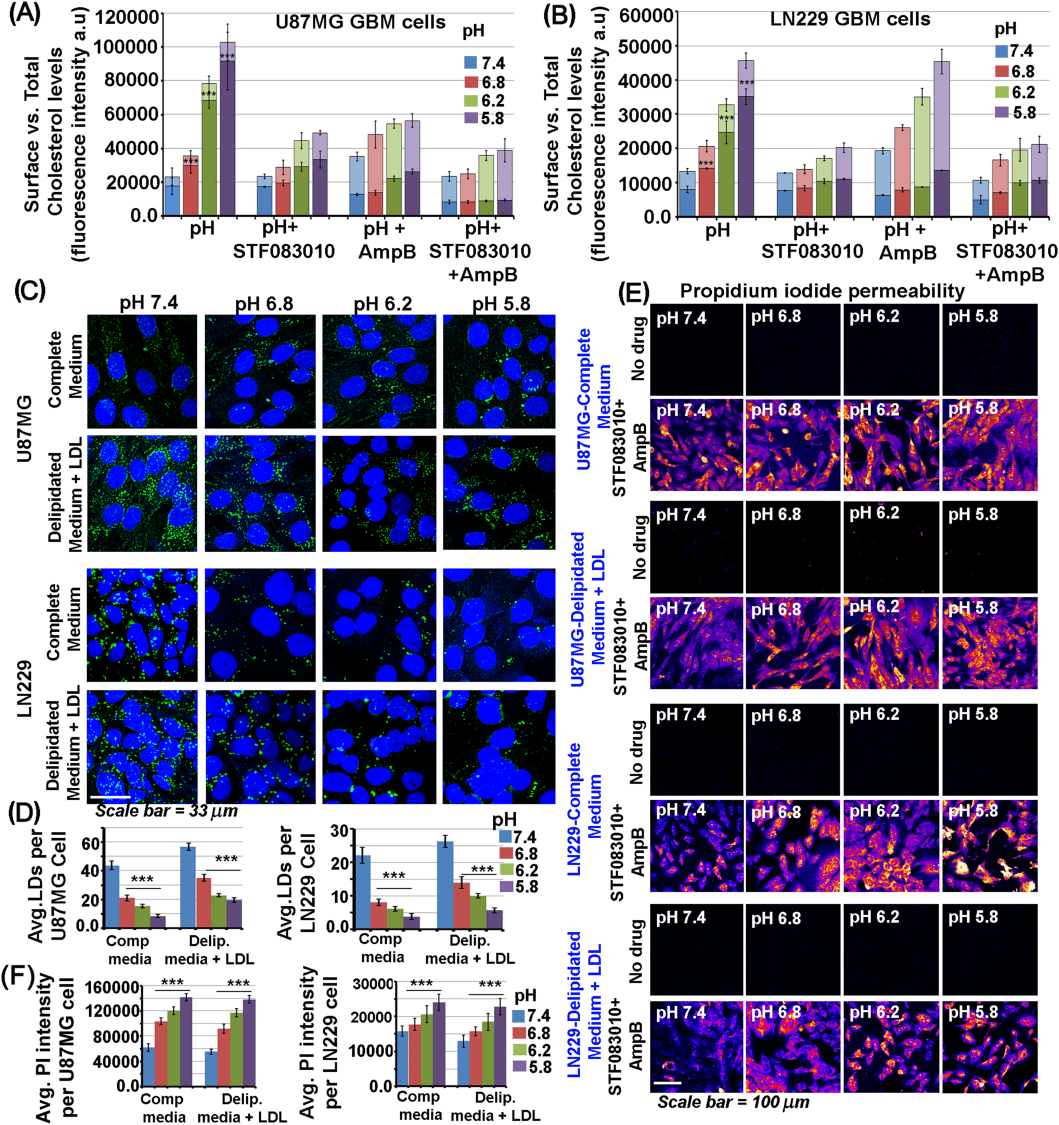
Attenuation of GM3‐IRE1‐sXBP1‐ACSS2‐cholesterol mechano‐machinery in astrocytic brain tumours by STF‐083010 and amphotericin B drug combination causes a simultaneous reduction in synthesis and plasma membrane levels of ‘excess’ cholesterol. (A,B) STF‐083010 (60 μM), Amphotericin B (10 μM) and STF‐083010 + Amphotericin B treated GBM tumour cells showed a dramatic decrease in total and cell surface cholesterol at various pH levels. For representative images, see Figures S26 and S27. (C,D) Supplementation of GBM cells with an exogenous source of cholesterol via low‐density lipoproteins (LDL) feeding in the delipidated medium did not show an increase in LDL cholesterol (detected via Bodipy 493/503, a marker of lipid droplets in live cells) at low pH values. The same was also observed when a complete medium (with 10% FBS) was used in pH treatments. This indicates that LDLs are not the primary source of surface cholesterol in low pH microenvironments. (E,F) GBM cells grown in complete media or in delipidated media with LDLs were incubated with different pH media for 4 h and were then treated with STF‐083010 (60 μM) + Amphotericin B (10 μM) in respective pH media for the next 12 h. Drug treated GBM tumour cells showed significant enhancement of cell permeability at all pH values, as measured by cellular intensities of cell impermeable dye propidium iodide (PI). PI uptake was performed for 3 min before fixing the cells. All datasets are reported as mean ± SD. Significance is shown as *p < 0.05, **p < 0.01, ***p < 0.001. Mean is derived from three independent experiments. In single‐cell measurements, recordings are made from at least 50 cells from each independent experiment of total *N* = 3

In summary, if we follow the strategy of inhibiting excess cholesterol biosynthesis with simultaneous sequestration of excess surface cholesterol, massive depletion of excess cholesterol from GBM tumour cells can be achieved (Figure 9A,B; for images, see Figures S26 and S27). On the one hand, patient data on GBMs document the use of acetate as a significant source of energy, wherein ACSS2 enables its conversion to Acetyl CoA for endogenous cholesterol and fatty acid synthesis [58]. On the other, *in vitro* and mouse GBM studies also show the dependence of GBM on low‐density lipoproteins (LDLs) as the source of cholesterol [59]. We find that LDL cholesterol was reduced in astrocytes growing in low pH microenvironments (Figure 9C,D). In contrast, HMGCR levels (a rate‐limiting enzyme in cholesterol synthesis) was increased (Figure S28), supporting the reports that endogenous cholesterol synthesis is required for GBM survival in acidic conditions.

To pursue this, we provided the exogenous source of cholesterol in the form of LDL particles to glioblastoma cells in the delipidated medium in various pH conditions. In this experimental set‐up, the sXBP1‐SREBP2‐ACSS2 mediated ‘excess’ cholesterol synthesis pathway was kept inhibited via STF‐083010 or with a more robust STF‐083010 and Amphotericin B combination treatment (Figure 9C,D). The results point to significant membrane leakage, suggesting that tumour cells heavily depend on the de novo synthesis of high cholesterol levels and exogenous uptake is not sufficient for an anti‐acid defence (Figure 9E,F).

### GM3‐IRE1‐sXBP1‐SREBP2‐ACSS2‐cholesterol axis is required for acid adaptation in astrocytes and astrocytic tumours

To further substantiate that the anti‐acid survival mechanism acts through the GM3‐sXBP1‐SREBP2‐ACSS2‐cholesterol axis, we performed several supporting experiments. In order to rule out that the possibility that GM3 can exercise its anti‐acidosis pro‐survival effects independent of sXBP1 cascade, we ran membrane permeability assays in sXBP1 depleted, but GM3 enriched astrocytes and astrocytic tumour cells. We found that membrane integrity was severely compromised in the absence of sXBP1 expression (Figure S29). However, transfection of sXBP1 plasmid in GM3 depleted cells could rescue membrane leakage (Figure S29). Hence, sXBP1 is an essential pro‐survival target of GM3 in combating acid stress.

sXBP1 has been previously shown to enable survival through c‐myc and HIF1α [60, 61]. We documented that there was an appreciable expression of c‐myc in sXBP1 depleted acidified mouse primary astrocytes. This confirms that sXBP1 is not the sole regulator of c‐myc expression and that sXBP1 does not mediate anti‐acid survival through c‐myc (Figure S30). We next performed in situ proximity ligation assay between HIF1α and sXBP1 in human/mouse astrocytes and GBM tumour cells depleted of sXBP1 targets SREBP2/ACSS2. We observed that although a significant HIF1α and sXBP1 interaction was present (Figure S31), the plasma membrane integrity was compromised if SREBP2/ACSS2 was downregulated in these cells exposed to acid stress (Figure S32). The same study further established that in both sXBP1 inhibited or GM3 depleted cells, plasma membrane leakage could be prevented by SREBP2 or ACSS2 expression via plasmid transfections (Figure S32). Hence, SREBP2/ACSS2 were the primary essential targets of sXBP1 in the anti‐acid survival response. Indeed, only in the presence of GM3 and sXBP1, the endogenous transcript levels of SREBP2 and ACSS2 were found to rise in astrocytes/astrocytic tumours under acid stress (Figure S33).

Further, the loss of either sXBP1, SREBP2 or ACSS2 significantly reduced the surface cholesterol, and the plasmid expression dependent rescue experiments showed that repletion of SREBP2 or ACSS2 in sXBP1 inhibited cells could restore the need for high surface cholesterol in acidic stress (Figure S34). Therefore, SREBP2 and ACSS2 were found to be crucial regulators of excess cholesterol synthesis.

We furthermore established that cholesterol is the essential final responder in the prevention of plasma membrane leakage, by supplementation of exogenous cholesterol in (i) GM3 depleted, (ii) sXBP1 depleted (iii) SREBP2 depleted and (iv) ACSS2 depleted astrocytes and astrocytic tumour cells under acidic stress (Figure S35). No leakage was observed upon cholesterol supplementation in any of the mentioned conditions.

In tumour dendritic cells, reactive oxygen species (ROS) intermediates have been reported to generate sXBP1, which promotes excessive lipid droplet accumulation impairing dendritic cells (DC's) anti‐tumour functions [62]. We document that in both complete and delipidated acidified media (devoid of LDL source of lipids), fewer lipid droplets are formed in astrocytes/astrocytic tumours (Figure S36), even though ROS levels were found to be higher in low pH conditions (Figure S36). So, the capacity of astrocytes and astrocytic tumours to handle ROS or lipid droplets accumulation differ from DC's. Again, when we inhibited ROS with Vitamin E treatment, we did not find any difference in either activated IRE1 clusters (Figure S37) or in the expression of sXBP1 in astrocytes and astrocytic tumours (Figure S38). Both IRE1 and sXBP1 were crucial targets downstream of GM3 (Figures S37 and S33). Hence, in our system, ROS was not associated with the activation of sXBP1. This is probably due to appreciable levels of ROS intermediates degrading proteins HO‐1 and NQO1 in astrocytes and astrocytic tumours exposed to low pH (Figures S39 and S40) [63, 64, 65].

**FIGURE 10:**
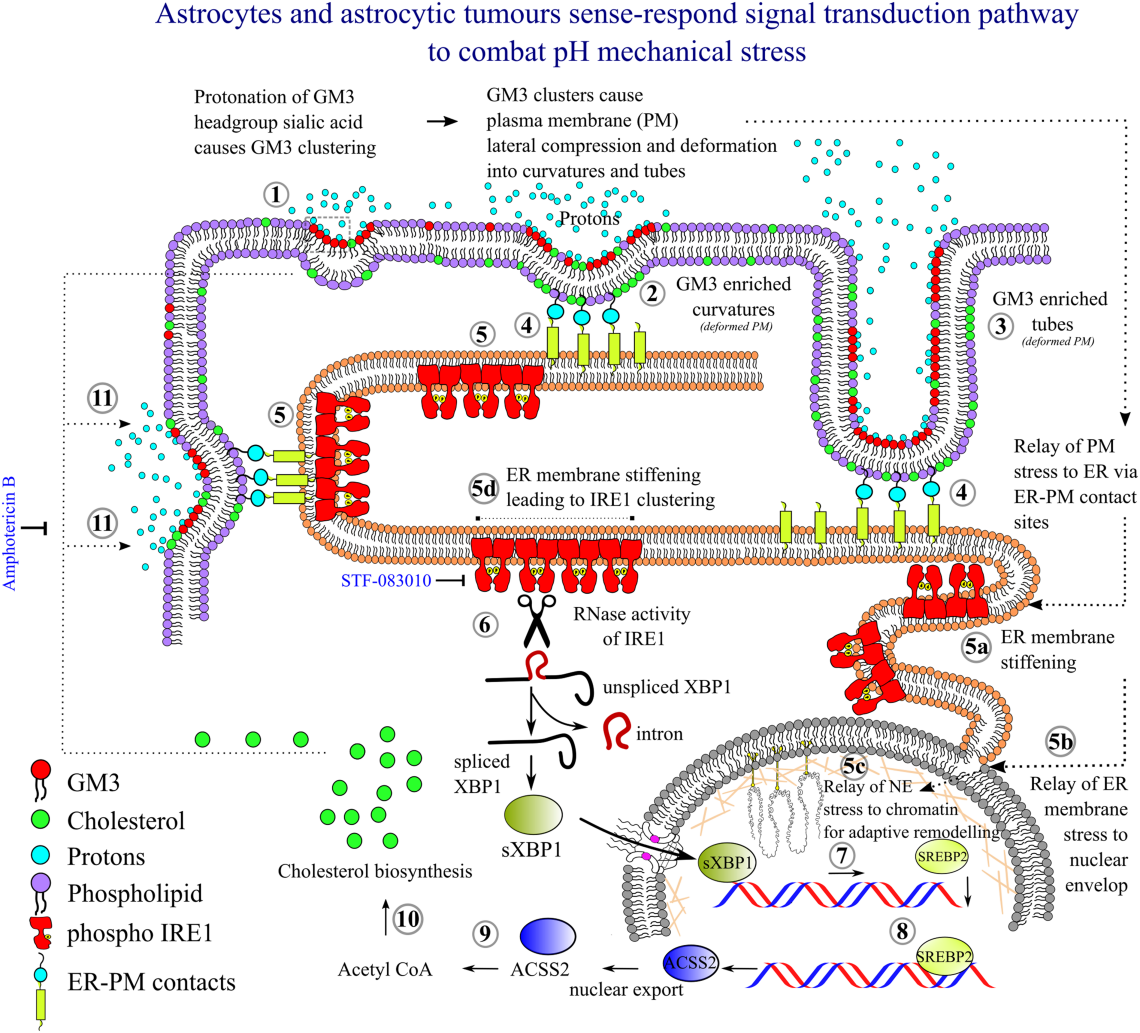
A working model of the sense and respond strategies generated by astrocytes and astrocytic tumours to combat extracellular pH stress. In both brain astrocytes and astrocytic tumour cells, (1) high microenvironmental proton concentrations induces protonation of sialic acid headgroup of GM3 glycosphingolipid leading to ligation with neighbouring GM3 molecules. (2) GM3 homo‐ligations leads to extensive GM3 clustering on the plasma membrane, inducing lateral membrane compression and mechanical imbalances, thereby deforming the membrane into curvatures. (3) Membrane curvatures transforms into tubes in intense GM3 clustered areas. (4) Both GM3 enriched curvatures and tubes show interaction with the peripheral ER via ER‐PM membrane contact sites, through which the mechanical stress in GM3 compressed plasma membrane regions is relayed to the ER network. (5a‐b) ER lipid bilayer stress relays membrane mechanical stress to the nuclear envelope (NE), which is continuous with the ER, and (5c) resultant nuclear stiffening allows appropriate chromatin remodelling, as chromatin is in direct contact with NE, to permit transcription of anti‐pH stress adaptation machinery. (5d) On the parallel platform, ER lipid bilayer stress causes IRE1 transmembrane protein oligomerization and activation of RNAase activity (6) which triggered splicing of the intron in XBP1 mRNA, generating sXBP1 mature RNA, which is translated and translocated to the nucleus. (7–11) In the nucleus, sXBP1 promotes the synthesis of SREBP2, which in turn synthesises ACSS2. ACSS2 enables cholesterol synthesis in the cytoplasm, which is trafficked to the surface to provide mechanical tenacity. GM3‐IRE1‐sXBP1‐ACSS2‐cholesterol axis is deduced as a unique anti‐acid stress survival mechano‐machinery in astrocytes which can be clinically translated by potentiating IRE1 activation, through known small molecules, in brain injuries and neurodegeneration involving acidosis. On the other side, suppressing IRE1 and depletion of surface cholesterol via STF‐083010 + Amphotericin B drug combination will be helpful as an anti‐astrocytoma/GBM therapeutics

Furthermore, we find that inhibition of ROS from astrocytes and astrocytic tumours exposed to low pH does not impact trafficking of higher cholesterol to the surface (Figures S41 and S42), but mild leakage was ensued even in GM3 enriched cells (Figure S43). This could be because ROS plays a crucial role in fluidizing the membrane and does not allow the membrane to become alarmingly rigid in pathological conditions such as acidosis. Hence, loss of ROS may make the membrane mildly leaky, although ROS by itself could not inhibit the loss of membrane integrity under acidosis, which is a unique function of the anti‐acidosis GM3‐IRE1‐sXBPI‐SREBP2‐ACSS2‐cholesterol synthesis machinery. Hence, a working model of the sense and respond strategies generated by astrocytes and astrocytic tumours to combat extracellular pH stress is presented in Figure 10 (please also see Figure S44).

## DISCUSSION

Our study presents new insights into the brain's acid stress adaptation and puts forward a mechanistic biophysical pathway on how acid stress is combated by the astrocytes (see Figures 10 and S44). The exact mechanism is further utilised in astrocytic tumours for survival under acid stress. Intriguingly, rising levels of extracellular proton concentrations are interpreted as gradients of mechanical force via protonation mediated homo‐clustering of surface GM3 glycosphingolipid. The clustered organisation, which occurs due to ligation of neighbouring sialic acid glycan moieties of GM3 in response to protonation, proportionately compresses the plasma membrane and raises the surface mechanical stress. Micron‐sized GM3‐clustered membrane invaginates as curvatures and tubes and tether peripheral ER, thereby ‘relaying’ the mechanical stress to ER membranes, causing multimerization and activation of ER membrane resident IRE1, which is known to be activated by ER lipid bilayer stress. Activated IRE1 multimeric clusters then induce splicing of XBP1, which upregulates survival pathways crucially involving SREBP2‐ACSS2 mediated cholesterol synthesis and trafficking to the surface. An increase in surface cholesterol thus provides mechanical stiffness and prevents acid‐mediated membrane leakage. Hence, low pH is combated by astrocytes through a unique survival mechano‐machinery that involves GM3 as a mechanosensor, activated IRE1 as a mechanotransducer, sXBP1‐SREBP2‐ACSS2 as mechano‐effectors and cholesterol as a mechanoresponder of acid‐stress. Therefore, activating this unique mechanical sense‐response axis in acid stress associated neurodegeneration may prevent disease progression, while modulating it will effectively target the astrocytic tumour growth.

Cholesterol synthesis and surface trafficking may be a sound survival strategy to acidosis, mainly because cholesterol provides structural support to the cell membranes. The increase in cholesterol levels in a lipid bilayer causes tighter packing of fatty acid tails for better membrane tenacity. It prevents the separation of the lipid bilayer into absolute liquid and absolute gel/solid phases that would otherwise lead to the formation of gaps between the two phases allowing cytoplasmic contents to leak out of the cell until these gaps are sealed via lateral diffusion of neighbouring lipids. Hence, an increase in surface cholesterol stiffens the plasma membrane, causing non‐permeability to undesirable solutes and prevents the membrane from acid‐induced leakage.

Conversely, since GBM tumour cells have significantly higher cholesterol synthesis than normal cells, especially in acidic niches, inhibition of ‘excess’ cholesterol synthesis pathway via IRE1‐sXBP1‐SREBP2‐ACSS2 axis attenuation seems to be a more rational strategy than profound use of statins. Targeting HMGCR, a rate‐limiting enzyme in cholesterol synthesis, via statin is highly counterproductive. Studies have shown that statin resistance develops in tumours due to multidrug efflux receptors on the surface. The statins are taken up by the normal cells, creating cholesterol synthesis abnormalities and negatively impacting other branches of the mevalonate pathway involved in cell protection [59]. Also, statins are required in very high doses to inhibit excess cholesterol synthesis in tumours, which impacts the normal functioning of other organs.

It is well established that excessive cholesterol is the Achilles' heel in GBM treatment [56]. So, in contrast to statins, targeting ‘excess’ cholesterol pathways via ACSS2 inhibition can effectively compromise the survival of GBM cells without adversely impacting the normal cells. ACSS2 can be inhibited by specific inhibition of the RNAse activity of IRE1 that generates sXBP1.

However, we found that inhibition of excess sXBP1 via STF‐083010 in combination with Amphotericin B, an FDA approved drug that sequesters cholesterol from the surface, was more effective in inhibiting GBM growth. The dosage of Amphotericin B used in our study has been determined to be non‐toxic in an animal model [57]. Amphotericin B has low CSF penetrance but has shown promising results in managing fungal brain infections and glioma stem cells elimination by immune activation [66, 67, 68].

GM3‐IRE1‐sXBP1‐SREBP2‐ACSS2‐Cholesterol acid ‘sense and respond’ machinery players have a far‐reaching involvement in neuroprotection. For example, the normally acidic nature of the hippocampus shows a shift to an alkaline environment in Alzheimer's Disease (AD), indicating a putative role of the acidic microenvironment in learning and memory [69]; correlatively, acidosis adaptation effectors deduced in our study, namely sXBP1 [70] and ACSS2 [71], are also involved in memory formation and cognition [70, 71]. Further, pharmacological activation of the IRE1‐sXBP1 pathway has been identified to reduce mutant amyloid precursor protein (APP) associated mitochondrial toxicity in cellular models of AD [72].

Activation of sXBP1 in astrocytes is documented to have a positive pro‐survival role during ischaemia, and hypoxic cerebral injury, as sXBP1 can activate ERAD and reduce the load of unfolded proteins [47, 53, 73, 74, 75, 76]. The major problem arises during the reperfusion stage, which triggers molecular players to which sXBP1 can bind and then cooperate to trigger apoptosis. Therefore, sXBP1, activated by the cell for protection, becomes a villain during the reperfusion phase. Considerations are that for providing the overall neuroprotection during the reperfusion phase, sXBP1 should be potentiated, while ER stress apoptotic players such as CHOP (with which sXBP1 interacts and produces adverse effects) should be simultaneously inhibited [77, 78, 79, 80, 81].

Not only during injuries, but routine brain activity levels also generate large amounts of CO_2_, leading to brain acidification, which our astrocytes can clear [14, 82]. Our study presents the underlying machinery that can do so. SREBP2 and ACSS2 are already documented as pro‐survival factors in the acidic microenvironment of pancreatic cancers [83]. confirming SREBP2 as a crucial component of anti‐acid stress adaptation machinery. Biosynthesis of SREBP2 in astrocytes is also shown to prevents neuronal cell death [84]. Hence, the clinical literature on the individual players in our acid stress adaptation machinery strongly supports their neurotherapeutic roles in various acidosis associated disorders. What is novel in this study is that amongst the multiple pathways that astrocytes and astrocytic tumours may upregulate to counter acid stress, GM3 induced IRE1‐sXBP1‐SREBP2‐ACSS2‐Cholesterol is an essential mechano‐machinery. This novel machinery provides us with a controllable handle to switch survival in astrocytes if they cannot do so independently, such as brain injuries or in biologically aged individuals. The same machinery can be switched off to target astrocytic cancers.

## FUNDING INFORMATION

This work was supported by the Department of Biotechnology, Ministry of Science and Technology, Govt. of India through the following grants: BT/RLF/Re‐entry/16/2011; BT/PR4959/MED/30/770/2012; BT/PR6331/GBD/27/403/2012; BT/PR26735/MED/31/378/2017 to RM. SJ is supported by Senior Research Fellowship from Indian Council of Medical Research (Fellowship Award ID:2014‐26530; File no. BMS/FW/SCR/201426530/MAR2015/04/KL/GOVT).

## CONFLICT OF INTEREST

The authors declare that they have no conflict of interest.

## ETHICS STATEMENT

Please note that the human tumour tissue arrays (cat nos. GL208 and GL806e) are directly procured from US Biomax Inc, USA. The link to obtain specification sheets of the mentioned tissue arrays are as follows: https://www.biomax.us/tissue-arrays/Brain/GL208, and https://www.biomax.us/c2/index.php?route=product/product&product_id=58843

This is a commercially established repository for procuring human tumour tissue samples and samples related to other pathologies. The tumour tissue arrays with Cat. No. GL208 and GL806e are classified according to WHO 2007, 3rd edition and WHO 2016, 4th edition respectively. Again, according to US Biomax Inc., post‐mortem normal brain sections in lane H with positions H6, H7, H8, H9 of tissue array, cat no.GL806e (B031) are from road accident victims. The company has disclosed its consent form format to the user. No further institutional ethical approval was required by the authors for the use of human tissues from the certified commercial source. The tissue arrays are procured according to the purchase and import ethics of Rajiv Gandhi Centre for Biotechnology (RGCB) under the purchase order nos. RGCB/PUR/16/372/PO/9965 and RGCB/PUR/21/343/PO/1854/FE.

The mouse astrocyte‐associated data and mouse CO_2_ inhalation experiments were performed on C57/Bl6J mice strictly according to the protocols approved by the Institutional Animal Ethics Committee (certificate number IAEC/164/RM/2012 and IAEC/707/RM/2018 respectively) of Rajiv Gandhi Centre for Biotechnology (RGCB).

## CONSENT FOR PUBLICATION

All authors agree to publication of the article.

## AUTHOR CONTRIBUTIONS

**Rashmi Mishra:** Conceptualization; Data curation; Formal analysis; Funding acquisition; Investigation; Methodology; Project administration; Resources; Supervision; Validation; Visualisation; Writing‐original and revised draft. **Sebastian John:** Data curation; Formal analysis; Investigation; Methodology; Validation; Visualisation; Writing‐original draft. **Gayathri K.G:** Investigation. **Aswani Krishna P:** Investigation.

### PEER REVIEW

The peer review history for this article is available at https://publons.com/publon/10.1111/nan.12774.

## Supporting information

Figure S1: Schematic representation of experimental procedures followed in the study for quick reference of the various experimental phases
Figure S2: Representative FRET‐based force probe ratiometric images of astrocytes incubated at different pH units. Related to Figure 1e
Figure S3: F‐actin stress fibre formation at low pH
Figure S4: Representative force probe FRET ratiometric images of astrocytes treated with neuraminidase and then exposed to different pH units, related to Figure 2a
Figure S5: Representative force probe FRET ratiometric images of astrocytes treated with siRNA of GM3 synthase and when GM3 synthase depleted cells were fed with GM3 lipid and then exposed to different pH units, related to Figure 2b
Figure S6: Representative force probe FRET ratiometric images of astrocytes fed with Bodipy FL GM1 and Rhodamine GM3 or Bodipy FL Lactosylceramide and Rhodamine GM3 and then exposed to different pH units, related to Figure 2f
Figure S7: Representative images of the colocalization of ER‐PM markers, GFP‐MAPPER and E‐Syt2 with GM3 lipid, related to Figure 3a
Figure S8: Nuclear Lamin A expression in scramble siRNA transfected or GM3S siRNA transfected astrocytes upon exposure to different pH units.
Figure S9: Lamin A and Sec61b colocalization upon exposure to different pH units.
Figure S10: Nuclear γH2AX levels in mock siRNA transfected or siRNA GM3S transfected astrocytes exposed to different pH units
Figure S11: Quantitation of nuclear γH2AX levels in mock siRNA transfected or siRNA GM3S transfected astrocytes exposed to different pH units
Figure S12: Astrocytes exposed to low pH show enhanced nuclear localization of H3K9Ac and H3K27Ac, epigenetic activators of transcription, in the presence of GM3
Figure S13: GM3 enhances co‐expression of sXBP1‐SREBP2‐ACSS2 lipogenesis axis in low pH microenvironment of astrocytes
Figure S14: sXBP1 is required for significant co‐expression of SREBP2‐ACSS2 lipogenesis axis in low pH microenvironment of mouse primary astrocytes
Figure S15: sXBP1 is required for significant co‐expression of SREBP2‐ACSS2 lipogenesis axis in low pH microenvironment of human astrocytes
Figure S16: Representative images of astrocytes' surface cholesterol levels in low pH units with or without STF‐083010 treatment and when pH treatments were given in delipidated media, related to Figure 4 h.
Figure S17: Surface expression and colocalization of LAMP2 with plasma membrane marker WGA in astrocytes exposed to different pH units.
Figure S18: *In vivo* mouse model of brain acidification faithfully upregulates CAIX/CA9 protein, a marker of the acidic microenvironment.
Figure S19: *In vivo* mouse model of brain acidification faithfully upregulates SREBP2 in the acidified brain only in the presence of sXBP1.
Figure S20: GRP78 and DNAJC3, the downstream cytoprotective targets of sXBP1 are inhibited by STF‐083010 treatment in low pH treated mouse primary astrocytes.
Figure S21: GRP78 and DNAJC3, the downstream cytoprotective targets of sXBP1 are inhibited by STF‐083010 treatment in acidified brain.
Figure S22: ATF4 and NF‐kB, the downstream cell death associated targets of ER stress are upregulated by STF‐083010 treatment in low pH treated mouse primary astrocytes.
Figure S23: ATF4 and NF‐kB, the downstream cell death associated targets of ER stress are upregulated by STF‐083010 treatment in acidified brain.
Figure S24: Surface expression and colocalization of LAMP2 with plasma membrane marker WGA in glioblastoma tumour cell lines exposed to different pH units.
Figure S25: Representative images of the expression of sXBP1 and ACSS2 in GBM tumour cell lines exposed to different pH units, related to Figure 8a,b.
Figure S26: Representative images of the surface and total cholesterol levels in U87MG glioblastoma tumour cell line exposed to different pH units, related to Figure 9a.
Figure S27: Representative images of the surface and total cholesterol levels in LN229 glioblastoma tumour cell line exposed to different pH units with or without drug treatments, related to Figure 9b.
Figure S28: mRNA expression of HMGCR in GBM cells exposed to different pH units, related to Figure 9c.
Figure S29: Propidium Iodide (PI) based membrane leakage assay shows that sXBP1 is a crucial downstream effector of GM3 in the generation of low pH adaptation in astrocytes and astrocytic tumours
Figure S30: c‐Myc, a crucial oncogene, is not a non‐redundant downstream target of sXBP1 and cannot by itself rescue astrocyte membrane leakage in a low pH microenvironment.
Figure S31: In situ proximity ligation assay (PLA) shows that HIF1α interaction with sXBP1is not reduced in astrocytes and astrocytic tumours cells that were depleted of SREBP2 and ACSS2.
Figure S32: Propidium Iodide (PI) based membrane leakage assay shows that SREBP2 and ACSS2 are the essential downstream prosurvival targets of GM3‐sXBP1 low pH adaptation machinery in astrocytes and astrocytic tumours
Figure S33: SREBP2 and ACSS2 transcript levels confirm that these are the essential downstream pro‐survival targets of GM3‐sXBP1 low pH adaptation machinery in astrocytes and astrocytic tumours
Figure S34: SREBP2 and ACSS2 are crucial downstream targets of sXBP1 in the enhancement of excess surface cholesterol in low pH treated astrocytes and astrocytic tumour cells
Figure S35: Propidium Iodide (PI) based membrane leakage assay shows that cholesterol is an essential pro‐survival responder in low pH adaptation machinery in astrocytes and astrocytic tumours
Figure S36: Lesser lipid droplets (LD) accumulation was found in astrocytes/astrocytic tumours exposed to low pH conditions vs the physiological pH, although ROS levels were found to be higher in low pH conditions
Figure S37: GM3 and not ROS enhances IRE‐1 clustering/activation in low pH treated astrocytes and astrocytic tumours.
Figure S38: ROS does not impact sXBP1 transcript levels in low pH treated astrocytes (human and mouse primary) and astrocytic tumours (LN229 and U87MG).
Figure S39: HO‐1 and NQO1, the downstream anti‐oxidant targets of ROS are activated in acidified astrocytes and astrocytic tumour cells.
Figure S40: HO‐1 and NQO1, the downstream anti‐oxidant targets of ROS, are inhibited by STF‐083010 treatment in acidified brains.
Figure S41: GM3 and not ROS enhances surface cholesterol in low pH treated astrocytes and astrocytic tumours.
Figure S42: GM3 and not ROS enhances surface cholesterol trafficking in low pH treated astrocytes and astrocytic tumours.
Figure S43: Depleting GM3 and not ROS causes membrane leakage in pH treated astrocytes and astrocytic tumours.
Figure S44: Self‐explanatory flowchart representation of the sense and respond strategies generated by astrocytes and brain tumours of astrocytic origins to combat extracellular pH stress.

## Data Availability

The datasets supporting the conclusions of this article are included within the article and its supporting information file.
