# Supplementary material for: Neurotherapeutic implications of sense and respond strategies generated by astrocytes and astrocytic tumours to combat pH mechanical stress
Source: Neuropathol Appl Neurobiol. 2021 Dec 9;48(2):e12774. doi: 10.1111/nan.12774 (PMC9300154; doi:10.1111/nan.12774)
Supplement: Supplementary file 1 — Figure S1: Schematic representation of experimental procedures followed in the study for quick reference of the various experimental phases Figure S2: Representative FRET‐based force probe ratiometric images of astrocytes incubated at different pH units. Related to Figure 1e Figure S3: F‐actin stress fibre formation at low pH Figure S4: Representative force probe FRET ratiometric images of astrocytes treated with neuraminidase and then exposed to different pH units, related to Figure 2a Figure S5: Representative force probe FRET ratiometric images of astrocytes treated with siRNA of GM3 synthase and when GM3 synthase depleted cells were fed with GM3 lipid and then exposed to different pH units, related to Figure 2b Figure S6: Representative force probe FRET ratiometric images of astrocytes fed with Bodipy FL GM1 and Rhodamine GM3 or Bodipy FL Lactosylceramide and Rhodamine GM3 and then exposed to different pH units, related to Figure 2f Figure S7: Representative images of the colocalization of ER‐PM markers, GFP‐MAPPER and E‐Syt2 with GM3 lipid, related to Figure 3a Figure S8: Nuclear Lamin A expression in scramble siRNA transfected or GM3S siRNA transfected astrocytes upon exposure to different pH units. Figure S9: Lamin A and Sec61b colocalization upon exposure to different pH units. Figure S10: Nuclear γH2AX levels in mock siRNA transfected or siRNA GM3S transfected astrocytes exposed to different pH units Figure S11: Quantitation of nuclear γH2AX levels in mock siRNA transfected or siRNA GM3S transfected astrocytes exposed to different pH units Figure S12: Astrocytes exposed to low pH show enhanced nuclear localization of H3K9Ac and H3K27Ac, epigenetic activators of transcription, in the presence of GM3 Figure S13: GM3 enhances co‐expression of sXBP1‐SREBP2‐ACSS2 lipogenesis axis in low pH microenvironment of astrocytes Figure S14: sXBP1 is required for significant co‐expression of SREBP2‐ACSS2 lipogenesis axis in low pH microenvironment of mouse primary astrocy [file NAN-48-0-s001.pdf]

Supplementary Materials For

Neurotherapeutic implications of sense and respond strategies generated by astrocytes and astrocytic tumours to combat pH mechanical stress

Sebastian John[1,2], Gayathri K.G[1,2], Aswani P. Krishna[1] and Rashmi Mishra[1,2*]

*Correspondence to: rashmimishra@rgcb.res.in; rashmi.mpi.cbg@gmail.com

Affiliations:

[1]*Brain and Cerebrovascular Mechanobiology Research, Laboratory of Translational Mechanobiology, Dept. of Neurobiology, Rajiv Gandhi Centre for Biotechnology, Thiruvananthapuram, Kerala, India-695014*

[2]*Manipal Academy of Higher Education, Manipal, Karnataka, India*

This file includes:

I) Supplementary Section 1: Supplementary Methods

II) Supplementary Section 2: Supplementary Figures with Legends

Supplementary Section 1

SUPPLEMENTARY METHODS

Additional reagents :

Cell lines were purchased from ATCC/Sigma/Lonza unless otherwise mentioned. The majority of the chemicals were obtained from Merck/Sigma-Aldrich. GM3-Na from bovine milk and GM3 TopFluor were from Avanti Polar Lipids, GM3 biotin was from Matreya LLC; Bodipy FL C5 Ganglioside GM1, Bodipy FL C5 Lactosylceramide and Bodipy 493/503 were from ThermoFischer Scientific; SNA lectin was from Vector Laboratories and siRNAs were from Santa Cruz Biotechnology Inc; Primary antibodies details with dilutions used are as follows: pIRE1(S724) (ab48187, 1:400); Cholesterol (abx100311, 1:100); LAMP2 (AF6228, 1:100), antibodies:SREBP2 (1:300) sc-13552; GRP78 (1:200) ab32618; DNAJC3 (1:100) ab70840; ATF4 (1:200) ab1371; NF-kB (1:30) sc-372; c-Myc (1:50) sc-40 ; HO-1 (1:100) ab52947;NQO1 (1:100) ab34173; H3K9Ac (1:400) ab12179;H3K27Ac-(1:400) ab4729;sXBP1 (1:400), MABC521;ACSS2 (1:400) 3658S; LDH (1:100); HIF1α (1:100) sc10790; anti-biotin (Jackson Immunoresearch laboratories, 200-002-211), pH2AX(S139) (ab11174, 1:300), GM3 (NBT-M101, 1:150); ACSS2 (cell signaling, cat no. 3658S, 1:200), sXBP1(MABC521, 1:100), H3K9ac (ab12179, 1:400), H3K27ac (ab4729, 1:400). In *in vitro* experiments, STF-083010 was used at a concentration of 60μM and Amphotericin B at 10μM.

Plasmids were either received as gifts or were purchased from Addgene as mentioned below: E-Syt2-mCherry and EGFP-E-Syt2 (Addgene #66831) was a gift from Prof. Pietro De Camilli, Yale University School of Medicine; Doxycycline inducible GFP-IRE1 construct was a gift from Prof. Peter Walter, University of California, San Francisco; GFP- SEC61B plasmid (Addgene #121159) was a gift from Prof. Christine Mayr, Memorial Sloan Kettering Cancer Center; pBABE-puro-GFP-wt-lamin A plasmid (Addgene #17662) was a gift from Prof. Tom Misteli, National Cancer Institute, NIH, USA; GFP-MAPPER plasmid was a gift from Dr. Jan Liou, University of Texas Southwestern Medical Center ; FRET Probe Actinin-sstFRET-GR plasmid was a gift from Dr. Tetsuya Kitaguchi for (Addgene#83416); pcDNA3.1-2xFLAG-SREBP-2 was a gift from Timothy Osborne (Addgene plasmid # 26807 http://n2t.net/addgene:26807;RRID:Addgene_26807;DOI10.1128/MCB.24.18.8288-300.2004); Ad-Flag-XBP1s was a gift from QingboXu&LingfangZeng (Addgene plasmid # 63678; http://n2t.net/addgene:63678; RRID:Addgene_63678; DOI: 10.1073/pnas.

090319710); ACSS2 was a gift from John Denu (Addgene plasmid # 13746 ; http://n2t.net/addgene:13746 ; RRID:Addgene_13746; DOI:) 10.1073/pnas.0604392103.

Methodologies:

1) Cell culture and pH treatments:

To assess the effects of low pHe, the pH of the media was altered with 2N HCl (hydrochloric acid) in accordance with previously published protocols[1–3]. It has been shown that pH adjustments with NaHCO3, lactate or HCl yield comparative results[4]. However, pH adjustment with HCl is the most frequently preferred method[2].

Since activity evoked pH in the brain is found to be as low pH 6.0 (5) , and in brain tumours, in vivo pH has been observed to be as low as pH 5.9 in localized regions of acidity[1–3,6–7], we used the following pHs-7.4, 6.8, 6.2 and 5.8 to explore the adaptive responses of astrocytes to acidosis.

Although hypoxia and nutrient starvation are known to generate acidosis, acid effects are independent of hypoxia and starvation[4]. So dissecting the acid effects *per se* is crucial.

SVG human astrocytes (NHA1) cells[8] were seeded for 16 hrs in MEM medium with 10% FBS and 1X antibiotic-antimycotic solution. NHA2 astrocytes, purchased from Lonza, were cultured in the medium and growth factors provided with the cells. Mouse primary astrocytes were isolated from P5 pups according to the protocol described by Schildge *et al.*[9].GBM tumour cell lines (U87MG, LN229 and U373) were cultured in DMEM containing high glucose, 10% FBS and 1X antibiotic/antimycotic solution. Cell seeding density for an 8 well chamber slide was 60,000 cells per well, 2,50,000 for a 6 well plate, 6000 cells for a 96 well plate, 26000 cells per well for a 48 well plate. The cells were then treated with MEM medium adjusted to pH 7.4, 6.8, 6.2 and 5.8. The cells were incubated at the specified pH for 4 hrs unless otherwise indicated. Fresh pH-adjusted media was added to cells every 2 hrs of treatment. Tumour cell lines were treated likewise unless otherwise indicated in specific assay protocols.

In experiments involving the reversal of pH to physiological values after four hrs treatment, the low pH media was replaced with buffer solution [Normal Medium Supplement (pH 7.4)].

The buffer treatment was given for the next 4 hrs, and then cells were fixed with 1.5% PFA (pH 7.4)

2) DPH dye-based membrane fluidity/ anisotropy analysis:

50000 cells were seeded in a 24 well format cell culture plate for 14 hrs. pH treatments were given for the next 4 hrs. Post-treatment, cells were washed with 1X HBSS and were incubated with 2µM DPH (1,6 Diphenyl-1,3,5-Hexatriene) in 1X HBSS for 1 hr at 37°C, in the dark. Post-treatment, cells were washed with 1X HBSS. Anisotropy measurements were acquired on TECAN Spectra Max multimode plate reader with a polarizer. The dye was excited at 365 nm, and emissions were collected at 430 nm in parallel and perpendicular planes. Em.and Ex. slits were kept at 5 nm. The fluorescence anisotropy (r) value was calculated using the equation $r = [(I_{\|} - I_{\perp}) / (I_{\|} + 2I_{\perp})]$, where $I_{\|}$ and $I_{\perp}$ are the fluorescence intensities oriented in parallel and perpendicular plane respectively, to the direction of the polarization of the emitted light.

3) Laurdan dye-based analysis of plasma membrane lipid packing order:

6000 cells were seeded and incubated for 24 hrs in a 96 well plate. Specific pH treatments were given for a total of 4 hrs with fresh pH medium change after 2 hrs. Post-treatment, the cells were washed with 1X HBSS. Cells were then treated with 5µM Laurdan dye along with 0.25% Pluronic F127. Upon treatment, the cells were left incubated for 20 min at 37°C, in the dark. After that, the cells were washed with 1X HBSS. The plates were read in the TECAN Spectramax machine, where the dye was excited at 375 nm, while dye emissions were collected at 440 nm and 490 nm. GP (General polarization) was then calculated by the following formula, GP= (Intensity at 440nm - Intensity at 490nm)/ (Intensity at 440nm + Intensity at 490nm).

4) Atomic force microscopy-based total force and cortical/surface stiffness analysis:

Astrocytes were cultured for 24 hrs in a 60mm dish, and respective pH treatments (pH 7.4, 6.8, 6.2, 5.8) were given. Post pH treatments, cells were fixed with 1.5% PFA, washed with

1X PBS and stored in 3 ml of 1X PBS. For force curves and cortical stiffness (Young's modulus) determination, data was acquired on Asylum MFP3D AFM (Asylum Research, CA) mounted on a Zeiss epifluorescence microscope, and cells were indented using a pyramid-tipped probe (TR400PB(L) 10KHz) with nominal spring constant of 28.45 pN/nm. Actual spring constants were determined using the thermal calibration method. At least 60 cells, in three independent experiments, were indented at first to a depth of 400 nm, slightly off the centre for better determination of cortical stiffness/surface elasticity. Cortical stiffness was determined by fitting each force curve using Gwyddion Software 2.3, and force curves were fitted till 1000 nm using the Hertz model for a pyramidal probe.

5) WGA (Wheat Germ Agglutinin) and SNA (Sambucus Nigra) lectins based surface glycocalyx detection:

WGA staining: Where-ever applicable, fixed cells were surface incubated with WGA (5µg/ml) for 10 min. Cells were briefly washed and counter stained with Hoechst 33342 (5µg/ml for 5 min) and mounted in the mounting media.

SNA staining: Biotin-labelled SNA lectin (1:100) was applied on the cell surface for 2 hrs, cells were washed, and the signal was developed with anti-biotin primary antibody followed by anti-mouse AlexaFluor 594 secondary antibody.

6) Rhodamine phalloidin based F-actin stress fibres probe:

Fixed cells were incubated in Rhodamine phalloidin at a concentration of 2µg/ml for 45 min. Cells were briefly washed and counterstained with Hoechst 33342 (5µg/ml for 5 min) and mounted in the mounting media.

7) Plasmids transfections:

For transfection of different plasmids such as FRET force probe[10], mCherry-ESyt2 [a gift from Prof. Pietro De Camilli][11], GFP-MAPPER [a gift from Dr Jan Liou][12], mCherry-LaminA (Addgene #17662) and Sec61b-GFP (Addgene #121159), 60000 cells were seeded in an 8 well-chambered slide (or as otherwise mentioned in the experiment) and then cells

were incubated for 24 hrs (in MEM for astrocytes; DMEM high glucose + 10% FBS + 1X antibiotic for tumour cells). Cells were transfected with (0.5µg) of plasmid probes using a jetPRIME transfection reagent. After 36 hrs of transfection, specific pH treatments were given to cells for the next 4 hrs. After treatment, cells were either further processed for respective live-cell assays or were fixed with 1.5% PFA (pH 7.4).

For transfection of the EGFP-1RE1 doxycycline-inducible construct (gift from Prof. Peter Walter)[13], 60000 cells were seeded in an 8 well chamber slide, and the slide was incubated for 24 hrs. EGFP-IRE1 plasmid (doxycycline-inducible) was transfected using jetPRIME transfection reagent. Cells were incubated for 36 hrs post-transfection. All the wells were replaced with a fresh medium containing a low dose of doxycycline (10nM concentration), and cells were left for 24 hrs for EGFP-IRE1 induction. Further, pH treatments were given for the next 4 hrs, and cells were fixed using 1.5% PFA**.**

8) FRET-based force sensor studies:

The FRET probe, Actinin-sstFRET-GR (Addgene#83416, EGFP-mCherry FRET pairs were tagged at the N-C termini of the spectrin and this linker DNA fragment was inserted between the actinin-head domain and actinin-tail domain[10,14,15]) was transfected into the cells using the jetPRIME transfection method. Post transfection, cells were incubated for 36 hrs and then pH treatments (pH 7.4, 6.8, 6.2, 5.8) were given for the next 4 hrs, followed by cell fixing and mounting. For pre-setting of FRET probe EGFP-mCherry image capture parameters, the donor channel was excited with 488nm laser, and the image was captured at the best focus, with efficient PMT, offset and laser power. The FRET measurements in at least 30 cells, in each pH condition per three independent experiments, were acquired with these image capture settings. The donor was excited, and the image in the donor channel and acceptor channel/ FRET channel (laser power of acceptor channel is kept null) was sequentially acquired in the best focal plane. ROI (region of interest) in the FRET channel was selected, and this area was bleached for 30 seconds. Post bleaching, the donor channel image was captured again to analyze the gain in fluorescence (due to inhibition of acceptor gain from donor emission). After this, the acceptor channel was excited by its own laser (561 nm) to capture the overall fluorescence of the acceptor channel in the same setting as that used for capturing the FRET signal. Overall, the images that were captured were (i) donor emission (pre-bleaching), (ii) FRET channel emission image (pre-bleaching), (iii) FRET

channel emission image-bleached in a specified ROI, (iv) FRET emission image-post bleaching, (v) donor emission gain-post bleaching, (vi) acceptor channel emission when excited with its own channel laser-post donor gain image capture. FRET ratiometric image (representing pixel to pixel FRET efficiency) was obtained from the offline version of the software used to perform FRET, and this depicts the 'extent of gain' in donor emission upon ROI bleaching in the FRET channel.

The initial intensity of each cell was normalized to obtain the FRET efficiency values. First, the FRET ratio using 'donor excited acceptor emission values' for each cell (FRET ratio) I_{FR} = Intensity of donor/Intensity of acceptor (Donor excited acceptor emission) was calculated (I stands for Intensity, FR stands for FRET ratio). Followed by this, the FRET ratio using 'acceptor bleached donor gain values' was calculated as follows: $I_{(FR\ AB)}$= Intensity of donor /intensity of acceptor (AB stands for FRET Ratio After photobleaching on the same cells). Further, the difference in the FRET ratios, $I_{Diff}= I_{(FR\ AB)}-I_{FR}$ was calculated. Following this, FRET energy efficiency in each pH treatment was calculated, which relates to how much original energy was transferred due to FRET, i.e., FRET Efficiency= $I_{Diff} / I_{(FR\ AB)}$. This value was then plotted for each condition. This value can also be expressed in percentage by multiplying the ratio obtained by 100.

9) siRNA protocol:

40000 cells were seeded on 8 well chamber slides and incubated for 24 hrs. If siRNA or mock siRNA/scramble siRNA treatment was to be performed in cells that were also to be transfected with a plasmid construct, then the plasmid probe was transfected first as described in the transfection protocol. 100pM siRNA was transfected using the jetPRIME method, and cells were further incubated for 36 hrs. pH treatments (pH 7.4, 6.8, 6.2, 5.8) were given for the next 4 hrs, and cells were either further processed for respective assays or were fixed with 1.5% PFA/1XPBS (pH7.4).

10) Neuraminidase treatment for the role of sialic acid:

Similar to pH experiment protocols, 60000 cells were transfected with FRET-based force probe in 8 well chamber slides for 36 hrs and then were treated with 0.3U/ml of

neuraminidase at room temperature for 1 hr. Post this, the pH treatments (pH 7.4, 6.8, 6.2, 5.8) were given along with neuraminidase for the next 4 hrs, and cells were fixed with 1.5%PFA/1XPBS.

11) GM3 lipid feeding for rescue/complementation experiments:

In order to test whether the effects produced due to loss of GM3 (via siRNA GM3S) were specific, GM3 lipid (conc.50µM) was fed to GM3 synthase downregulated cells (GM3S or ST3Gal5). GM3 lipid was fed for 1 hr at RT before pH treatment. During lipid feeding and pH treatments, siRNA was kept in the medium. Post feeding, the cells were given respective pH treatments for various assays.

12) GM3 lipid Homo-FRET:

For lipid GM3-GM3-homo FRET: GM3-biotin and TopFluor-GM3 were fed to cells in equimolar concentrations (3.75µM) for 1hr at RT before pH treatments. Cells were then given pH treatment (pH 7.4, 6.8, 6.2, 5.8) and fixed with PFA. Rhodamine Red conjugated streptavidin was incubated on the surface to bind to biotin-GM3 to enable its visualization. The FRET assay was performed with the same microscopy set-up as described in the previous section. TopFluor GM3 is labelled as TF-GM3, and Rhodamine red GM3 is labelled as RR-GM3 in the figures.

In the case of GM1-GM3 and LactosylCer-GM3 FRET, 3.75µM biotin-GM3 was fed to cells in equimolar concentration to BodipyFL GM1 or LactCer-BodipyFL for 1 hr at 37°C. pH treatments were subsequently followed for 4 hrs, and the cells were fixed. Rhodamine Red conjugated streptavidin was incubated on the surface to bind to biotin-GM3 to enable its visualization. Note that BodipyFL and Rhodamine Red are efficient FRET pairs. TopFluor and Rhodamine are also efficient donor-acceptor FRET pairs. BodipyFL GM1 is labelled as BD-GM1, and BodipyFL Lactosylceramide is labelled as BD-LactCer in Figures. Peak excitation and emission wavelengths of the FRET fluorophores are as follows: TopFluor Ex:495nm, Em:503nm; Bodipy FL Ex: 503nm, Em:511nm; Rhodamine Red X, Ex:572nm, Em: 591nm.

13) STED microscopy:

Abberior STED system (Steadycon) was installed on Olympus IX83. Immunocytochemistry was performed according to the manufacturer's protocol using Abberior STAR 580 secondary antibody for anti-GM3 antibody signal detection. The cells were coverslipped with glass coverslips of thickness 170µm. 2D STED images were acquired at a resolution of 60-50nm. Surface plots of GM3 enriched topologies were generated in Fiji using the Surface plot plugin.

14) ER membrane fluidity analysis with merocyanine 540:

6500 cells were seeded on a 96 well glass bottom plate with 600µl of the medium. The plate was incubated for 24 hrs. The plasmids (EGFP-ESyt2 or EGFP-Sec61b) and siRNA GM3S were transfected into the cells together (Dual transfection using JetPRIME Method), wherever applicable.

Transfected plates were incubated for 36 hrs followed by two medium changes in 12 hours intervals. After transfection, pH treatments (pH 7.4, 6.8, 6.2, 5.8) were given for 4 hrs. At the end of the treatments, cells were washed twice with Krebs buffer, followed by 3µM merocyanine 540 incubation in the dark for 15 min. After incubation, the cells were rewashed with Krebs buffer and incubated at 37°C for 15 min.

For image capture, the first 488nm laser was used to obtain the EGFP plasmid signal. Then merocyanine 540 dye was excited with 561nm laser, and emission was collected between 562-580nm (spectral imaging), keeping the gain at 15 and laser power at 10.0, Pinhole-1.2 A.U, Zoom-1.0, scan size 512, scan speed-1, count 2 in all acquisitions. The EGFP intensity images were processed with respective merocyanine intensity images for determination of the extent of colocalization of ER membranes markers with merocyanine 540 as a membrane fluidity marker[16,17].

15) sXBP1 generation inhibition:

60000 cells were seeded in 8 well chamber slides and were incubated for 36 hrs. Cells were then treated with the IRE1 RNAse activity inhibitor (STF-083010) at a concentration of

60µM for 6 hrs[18]. Cells were given pH treatments (pH 7.4, 6.8, 6.2, 5.8) for the next 4 hrs along with the inhibitor. Post-treatment, cells were either processed for respective assays or were fixed with 1.5% PFA for 20 min at RT and washed with 1XPBS 4 times.

16) Mouse brain acidification studies:

Brain acidification was performed with an excess CO2 inhalation method. The protocol followed has been as described in Magnotta *et al.*[5] but with some modification. 6 mice pups (C57BL/6) at day 5 after birth were first injected subcutaneously with STF-083010 (10mg per kg body weight as described [19]) in 50µl saline over the dorsal cranium. Another set of 6 pups were injected with saline alone. 15mins after injection, 3 saline-injected and 3 STF-083010 injected pubs were transferred to a 7% CO2 incubator for CO2 inhalation for 2.5 hrs. Another 6 pups (3 saline-injected and 3 STF-083010 injected) were kept in a normal atmospheric environment. Post 2.5 hrs, pups were sacrificed by hypothermia, and the brains were immediately dissected out and fixed overnight in 4% PFA at 4°C. The following day, excess fixative was removed by washing the brains with 1X PBS and processed for paraffin embedding. The brains were sectioned in the sagittal plane at a thickness of 10µm. Near mid-sagittal sections were chosen for further analysis.

Validation of key results in mouse primary astrocytes: Mouse primary astrocytes isolated from P5 pups were subjected to the same experimental protocols as human astrocytes to confirm the conclusions further. The results are presented in the supplementary sections of the manuscript.

17) Real-time PCR:

Total RNA was extracted from cultured cells with the TriZol method, and reverse transcription into cDNA was performed with AB biosystems cDNA synthesis kit. RT PCR was performed using the SYBR green method as described in the user's protocol of AB Biosystems real-time PCR reagents and AB Biosystem Real-Time PCR user's manual. The comparative *C*T method was used for analyzing Real-Time PCR data and was expressed as a

fold change in gene expression upon pH treatments. Human gene Primer sequences used are as follows:

i) sXBP1:

Forward: CTGAGTCCGAATCAGGTGCAG,

Reverse: ATCCATGGGGAGATGTTCTGG

ii) Ubiquitin (UBA52):

Forward: GCCTGCGAGGTGGCATTATTGA,

Reverse: TTCTTGCGGCAGTTGACAGCAC

iii) SREBP2:

Forward: CTCCATTGACTCTGAGCCAGGA,

Reverse: GAATCCGTGAGCGGTCTACCAT

iv) ACSS2:

Forward: GGTGACCAAGTTCTACACAGCAC,

Reverse: GTTCACCCACTGTGCCTAACAC

v) DNAJC3:

Forward: GGAGAGGATTTGCCACTGCTTTT,

Reverse: CTCTGCTCGATCTTTCAGGGCA

vi) DNAJB9:

Forward: GCCATGAAGTACCACCCTGACA,

Reverse: TCGTCTATTAGCATCTGAGAGTGT

vii) HMGCR:

Forward: GACGTGAACCTATGCTGGTCAG

Reverse: GGTATCTGTTTCAGCCACTAAGG

viii) ST3GAL5/GM3S:

Forward: AGAGCCTCAGTCAAGGTTCTGG,

Reverse: GAGGTCATATCCAAAACCCGCC

18) Immunostaining:

Wherever applicable, cells were fixed for 20 min in 1.5% PFA, permeabilized with 0.25% saponin for 20 min, blocked for 1 hour with 5% BSA containing 2% normal serum in 1XPBS and primary antibody was then incubated overnight at 4°C. Following primary antibody incubation, cells were washed with 1X PBS and incubated with anti-isotype specific AlexaFluor conjugated secondary antibody (Jackson Immunoresearch) at a concentration of 1:200 in 1X PBS for 1 h at room temperature in the dark. Cells were briefly washed and counterstained with Hoechst 33342 (5µg/ml for 5 min) and coverslipped with mounting medium. Images were captured using a Nikon confocal microscope in 60X oil immersion objectives with NA 1.29. Please note that in a few experiments, double and triple immunostaining were performed per section, to detect different antigens and the images were acquired in the region of interest (ROI).

19) GM3 Immunostaining

60000 cells were seeded in a 8 well chamber slide and incubated for 24 hrs. After 12 hrs, half of the medium was replaced with a fresh medium. Specific pH treatments were given for 4 hrs. Fresh pH media was added every 2 hrs to maintain the pH conditions. After pH treatments, below described steps were followed:

(i) To detect GM3 on the surface of live cells,

The media in the chambered slide were changed with cold PBS++, and then the slides were shifted to 4°C cold room for further processing. After 10 min, the GM3 antibody (1:150) was incubated on the cells for 1 hour at 4°C. After 1 hour, the antibody was removed, and the cells were fixed with 1.5% cold PFA for 20 min at 4°C. A quick wash with cold PBS++ and then 5 washes with 1X PBS for 5 min each were given. The cells were then incubated with

secondary AlexaFluor 488 anti-IgM antibody (1:200) for 1 hour at RT. Post incubation, the cells were Hoechst stained and mounted in 70% glycerol/1X PBS for imaging.

(ii) To detect GM3 on the surface of fixed cells. After pH treatments, the cells were fixed in 1.5% PFA for 20 min at RT. Post fixation, the cells were washed with 1X PBS 4-5 times. Then the cells are incubated with 5% BSA+ 2% normal serum block for 30 min. No detergent permeabilization step was followed. Cells were subsequently incubated with primary anti-GM3 antibody (1:150) for 16 hrs at 4°C. Cells were again washed 4 times with PBS. Then secondary antibody was incubated on cells for a further 1 hr. Cells were washed, and Hoechst stained for 5 min and later mounted in 70% glycerol for imaging.

20) Immunohistochemistry on mouse brain sections and human tissue array:

Pre-processing: The tissue array slides purchased from US Biomax Inc. were as follows: Human Astrocytoma/Glioblastoma (GL208 with Grade I, II, III and IV; GL805a with Grade II, III and IV and GL806e with Grade III and Grade IV patients' tumour tissues). GBM tumour samples; Grade I and Grade II astrocytoma slides were also purchased from Abcam. The classification of tumour tissue into different grades was performed by the US Biomax Inc. pathologists via examination of the histological features, as per the WHO norms. Please see the Human ethics statement section in the main manuscript for details. The ischaemic brain sections were mounted in lane H of US Biomax Inc. tissue array GL806e. The donors were road accident victims. A declaration on the legitimacy of tissue resources that were used to make tissue-derived products supplied by US Biomax, Inc. was provided to the users.

The slides were pre-baked at 55°C on a hot plate for 15 minutes and incubated in fresh xylene for 10 min for de-paraffinization. The excess xylene was slowly drained, and the slide was re-incubated in fresh xylene for 10 min. Following xylene treatments, the slides were first immersed in chloroform for 5 min to remove any xylene and then immersed in 100%, 90%, 80% and 70% ethanol (5 min each) for rehydration. The tissues were washed (twice) after that in 1X PBS (pH 7.4) and then permeabilized using 0.025% saponin/0.01% digitonin for 30 min at room temperature. The slides were then washed 5-6 times with 1X PBS and were further processed for immunofluorescence as described below. The mouse brain sections were processed similarly.

Immunofluorescence: The tissues were incubated with blocking buffer (5% BSA with 2% Donkey Serum) for 1 hour and 30 min. After blocking, the tissue arrays were washed with 1X PBS and were incubated with primary antibody [anti-GM3 (1:50), anti-Cholesterol, (1:100), and anti-LAMP2, (1:100)] for 36 hrs at 4°C. Post incubation, the slides were properly washed with PBS (pH 7.4) and were further incubated with secondary antibodies mix for 90 min at RT. If applicable, slides were further stained with WGA AlexaFluor 594 (5µg/ml) for 45 min. The slides were appropriately washed with 1X PBS and were stained with Hoechst 33342 for 5 min before mounting them in 70% glycerol. For ACSS2 and sXBP1 immunostaining, antigen retrieval with 0.1M tri-sodium citrate (pH 6.0) step was included in the protocol.

21) MTT based survival assay:

GBM tumour cells: U87MG, LN229, U373 and normal cells: SVG-astrocytes, neurons-HT-22, HaCat-keratinocytes were cultured in 48 well corning costar plates at a density of 26,000 cells/well for 24 hrs. pH treatments with or without drugs (STF-083010 and Amp B) were followed for 48 hrs. Standard MTT assay procedure was followed. Absorbance was read at OD=490 nm, and reference was set at OD=655nm with a bandwidth of 9 nm and 25 flashes in each well using TECAN M200 multimode reader.

22) Surface Cholesterol detection:

For experiments in 10% delipidated serum-containing medium, 60000 cells were seeded on 8 well-chambered slides. Cells were kept in a medium containing de-lipidated serum for 2 hrs before pH treatments.

After this incubation, pH specific treatments in complete and delipidated serum-containing medium were performed. For inhibiting IRE RNAse activity, the cells were treated with STF-083010 (60µM) for 6 hrs before pH treatment. Cells were then incubated with pH media for 6 hrs. In wells where STF-083010 was pre-incubated, STF-083010 was maintained in the media during pH treatments. Post pH treatments, the surface of live and fixed cells were probed for cholesterol via nystatin and anti-cholesterol antibody separately, as described below:

(A) Surface Cholesterol detection with anti-cholesterol antibody:

(i) Live cells:

The pH treated cells were transferred at 4°C (cold room). Then primary antibody (anti-cholesterol, 1:100) was incubated on the cells' surfaces for 1 hrs at 4°C. Cells were fixed at 4°C with cold 1.5% PFA. Post fixation cells were washed properly for any residual PFA, and then cells were blocked with 5% BSA/1X PBS for 30 min at RT. Surface cholesterol signal was developed with AlexaFluor 488 conjugated secondary antibody, incubated at room temperature for 1 hour. Cells were rewashed with 1X PBS and then stained with 1X Hoechst for 5 min. Slides were mounted in 70% glycerol/1X PBS for imaging.

(ii) Fixed Cells: Post pH treatments, cells were fixed at RT with 1.5% PFA. Primary (anti-cholesterol) antibody and AlexaFluor 488 conjugated secondary antibodies were incubated on the surface for 40 min following standard immunostaining protocol but without permeabilization. Cells were washed 3 times and were mounted with 70% glycerol/1X PBS for imaging.

(B) Surface Cholesterol detection with Nystatin Staining

(i) Live cells: 50000 cells were seeded in 600µl of the medium in an 8 well chamber slide and incubated for 24 hrs. Post pH treatments, chamber slides were shifted to 4°C for 10 min. Cells were washed once with cold 1X PBS, and 50µg/ml of nystatin was incubated on the surface for 40 min. Nystatin was then removed from the wells, and the cells were fixed with 1.5% PFA (cold). The slides were fixed on ice for 30 min. Slides were consecutively washed thoroughly with 1X PBS and mounted with 70% glycerol/1X PBS for imaging.

Fixed Cells: Post pH treatments, cells were fixed at RT with PFA. Nystatin was incubated on the surface for 40 minutes; cells were then washed 3 times and mounted with 70% glycerol/PBS for imaging.

23) Total vs Surface cholesterol analysis:

Cells were given pH treatment for 4 hrs. Post this, the respective pH media were replaced with fresh pH media with or without drugs (STF-083010, Amphotericin B, STF-083010 + Amphotericin B) for the next 12 hrs. Cells were then fixed in 1.5% PFA.

After fixing the cells, the anti-cholesterol antibody was incubated on the cell surface for 24 hrs at 4°C. The signal was developed with anti-rabbit AlexaFluor 594. The cells were again fixed with 1.5% PFA, washed with 1X PBS and permeabilized with 0.01% digitonin (to reveal total cholesterol pool), followed by 3% BSA +10% normal serum block. The cells were then incubated with an anti-cholesterol antibody for 16 hrs, and the signal was developed using AlexaFluor 488. Both surface and total cholesterol signals were acquired via sequential imaging through confocal microscopy. Surface and total cholesterol signal intensities per cell (60 cells in each pH condition, in 3 independent experiments) were measured via Fiji software.

24) Spheroid/ pellet culture assays:

200000 cells were pipetted out in 200µl of media in each well of a round bottom 96 well plate, and the plate was centrifuged at 500g for 5 min to make the cell pellet/adhered sphere. Cell pellets/ adhered spheroids were incubated in DMEM high glucose with EGF (20ng/ml), FGF (20ng/ml) and B27 1X supplement. Post 48 hrs, spheres were either given only pH treatments or treated with drugs (STF-083010, Amphotericin B, STF-083010 + Amphotericin B) in respective pH media. Treatments were replaced with fresh treatments every 24 hrs. Sphere growth was monitored, and images were captured till 5 days of treatments. Volume analysis in each pH condition, with or without drugs, was performed using the ReViSP tool [Reconstruction and Visualization from a Single Projection (ReViSP) tool]. This tool enables the reconstruction of 3D volume by counting the voxels (3D pixels).

25) Colony Maintenance and Growth assay:

Cells were seeded in each well of a 6 well plate. Seeding densities were 100 cells per well for U373 and LN229 and 500 cells per well for U87MG. This was to allow single cells to form clonogenic colonies in 8 days. The cells were incubated in DMEM high glucose, 5% FBS with 1X antibiotic antimycotic solution and the medium was changed every 24 hrs. After the colonies were formed, these cells were given specific pH treatment for 4 hrs. After initial pH treatment, drugs (STF-083010, conc.: 60µM; AmpB, conc,: 10µM, STF-083010 + AmpB) were added to colonies in respective pHs in three independent experiments, and the experiment was continued for another 24 hrs. Post 24 hrs of drugs treatments, the cells were

fixed in 3:1 methanol: glacial acetic acid and stained with 0.5% crystal violet in methanol. Stained plates were air-dried and were stored in the dark. The whole well view was acquired by motorized stitching of the Olympus FV3000 confocal microscope images under 4X objective, bright field settings. Colonies with more than 50 cells were scored in Fiji software. The number of colonies per pH condition, with or without drugs treatments were graphically represented.

26) LDL cholesterol detection and Propidium Iodide (PI) based cell permeability assay:

Two hrs before pH treatments, cells were incubated with a medium containing 1% delipidated serum. 5µg/ml of LDL in pH media (pH 7.4, 6.8, 6.2, 5.8) containing delipidated serum was administered to tumour cell lines grown in 8 well chamber slides. After 4 hrs of pH treatment with LDL, the medium was replaced with or without drugs (STF-083010, conc.: 60µM; AmpB, conc.:10µM, STF-083010 + AmpB) in respective pH with LDL for another 12 hrs, to understand the effect of drugs at respective pHs in the presence of LDL. Post 12 hrs treatments, Bodipy 493/503 (conc: 10µM) was added to respective wells for 30 min to identify LDL cholesterol. Post this 5µl of propidium iodide (conc.: 1mg/ml) was added to each well for 3 min. The cells were immediately fixed and were imaged to identify the cell permeability in LDL treated cells, with or without drugs treatments, in different pH incubations. Similar experiments were also performed in a medium containing 10% foetal bovine serum, where the serum served as the source of LDL; hence no LDL was added.

27) Sytox green-based cell permeability analysis:

7000 cells were seeded in a 96 well plate (Corning costar assay plate) and incubated for 24 hrs at 37°C. STF-083010 treatment was initially given for 6 hrs or not. Post this, specific pH treatments were administered for the next 4 hrs, and then sytox green at a concentration of 167nM was added to each well and cells were incubated in the dark for 30 min. The cells were washed 2 times in 1X HBSS, and sytox green permeability in cells was recorded by reading the plate in a TECAN multimode reader with 488 nm excitation and 525 nm emission settings.

28) Brain Tumour Kaplan Meier Survival Analysis:

sXBP1 and ACSS2 survival association and prognostic survival value in glioma/astrocytoma patients were obtained from PrognoScan: http://dna00.bio.kyutech.ac.jp/PrognoScan/). XBP1 data were from the GSE4271-GPL96 astrocytoma dataset [contributed by Philip, probe name: 200670_at (HG-U133A) with 21 grade III and 51 grade IV patient samples] and from the MGH-glioma dataset [contributed by Nutt, probe name: 39755_at (HG-U95A) with 22 astrocytoma and 28 glioblastoma patient samples]. ACSS2 data was from the GSE4412-GPL97 glioma dataset [contributed by Freije, probe name: 234312_s_at (HG-U133B), with 8 astrocytoma and 50 glioblastoma patient samples].

29) Vitamin E/Tocopherol Treatment:

Requisite cell numbers were plated in 8 well chamber slides and incubated for 24 hours before treatment. Cells were treated with a pH adjusted medium along with 100µM Vitamin E and were then incubated for the next 8-12 hours followed by fixation with 1.5%PFA or were processed for other assays such RNA extraction/real-time PCR, DCF-DA (ROS assay), Nystatin etc.

30) Image Capture settings:

The followed protocols were similar to those described in Rharass, T. et al (Journal of Biological Chemistry, 2014) and John et al. [20]. Cell morphologies were observed in transmitted light to ensure no cell death occurred during the experimental time frame, before pH treatments. The image capture settings, namely, detector gain and offset, pinhole size, laser power, confocal section thickness, zoom factor, line and frame averaging, were kept constant for all comparative sets of experiments. Using Fiji image analysis software, brightness/contrast adjustments were applied equally to every pixel in the images (i.e. maximum projections) for each comparative set. No change to gamma settings was applied. Regions of interest (ROIs) were set individually, i.e. for each cell of the population in the images, based on cell boundaries to calculate the mean fluorescence intensities (ratio of the sum of fluorescence intensity emitted to the number of pixels in the ROIs). All data were

obtained from at least 3 independent experiments. For each time point or treatment, at least 5 images per independent experiment were recorded. Results are shown as means ± S.D.

31) Images analysis and statistics:

Protein integrated densities: Protein profile in cytoplasmic and nuclear fractions of the cells were derived either by using ROI freehand tool followed by 'Measure' application in Fiji software or by using the Cell Profiler software.

GM3 and IRE1 cluster analysis:

The cluster signals were identified against the background using the threshold and binary tools embedded in Fiji image analysis software. Subsequently, the 'Measure' tool was used to retrieve each cluster's area and intensity values. The area values were in micrometre square. The minimum size of the cluster was set automatically by the software and was described by a pixel unit of 0.17μm2.

***Colocalization*:**

The images colocalization statistics were produced using the Coloc2 plug-in in Fiji Software (freely downloadable from- http://pacific.mpi-cbg.de/). Briefly, we used the following measure: Rr= Pearson's colocalization coefficient for the image. R= Mander's correlation coefficient indicates the colocalized yellow pixels in a Red: Green channel setting of the two images probed for colocalization. Robs = Pearson's coefficient for observed sample sets. Rand = Pearson's coefficient for random or chance occurrences. Robs was higher for all colocalization analyses with P =1, showing that R and Rr values are reliable.

32) Image Presentation:

In specific data figures, the images were presented as pseudo-coloured index images, using fire LUT. At a single cell and tissue level, the lowest to highest intensity of expression can be easily visualized by this format using the calibration bar as a guidepost.

33) Statistical analysis:

Multivariant tests were performed using ANOVA in Microsoft Excel. Cluster area analysis was performed in FIJI software, and results were plotted using GraphPad Prism Software. Statistical analyses were performed using one-tailed unpaired Bonferroni's t-test. Significance was represented by the following p-values: $*p \leq 0.05$, $**p \leq 0.01$ and $***p \leq 0.001$. All comparisons were made with pH 7.4 conditions unless otherwise indicated. Data are presented as means ± S.D. and averaged from at least 3 independent experiments. For single-cell analysis, at least 50-200 cells were captured in 5 random fields per condition in 3 independent experiments were used. Image analysis was done in each condition using Fiji image processing software. The calibration bar for LUT converted images are shown in the respective Figures.

References:

1. Reichert M, Steinbach JP, Supra P, Weller M. Modulation of growth and radiochemosensitivity of human malignant glioma cells by acidosis. Cancer. 2002 Sep;95(5):1113–9.

2. Mashima T, Sato S, Sugimoto Y, Tsuruo T, Seimiya H. Promotion of glioma cell survival by acyl-CoA synthetase 5 under extracellular acidosis conditions. Oncogene. 2009 Jan;28(1):9–19.

3. Hjelmeland AB, Wu Q, Heddleston JM, Choudhary GS, MacSwords J, Lathia JD, et al. Acidic stress promotes a glioma stem cell phenotype. Cell Death Differ. 2011 May;18(5):829–40.

4. Kondo A, Yamamoto S, Nakaki R, Shimamura T, Hamakubo T, Sakai J, et al. Extracellular Acidic pH Activates the Sterol Regulatory Element-Binding Protein 2 to Promote Tumour Progression. Cell Rep. 2017 Feb;18(9):2228–42.

5. Magnotta VA, Heo H-Y, Dlouhy BJ, Dahdaleh NS, Follmer RL, Thedens DR, et al. Detecting activity-evoked pH changes in human brain. Proc Natl Acad Sci U S A. 2012 May;109(21):8270–3.

6. Vaupel P, Kallinowski F, Okunieff P. Blood flow, oxygen and nutrient supply, and

metabolic microenvironment of human tumours: a review. Cancer Res. 1989 Dec;49(23):6449–65.

7. Gerweck LE, Seetharaman K. Cellular pH gradient in tumour versus normal tissue: potential exploitation for the treatment of cancer. Cancer Res. 1996 Mar;56(6):1194–8.

8. Major EO, Miller AE, Mourrain P, Traub RG, de Widt E, Sever J. Establishment of a line of human fetal glial cells that supports JC virus multiplication. Proc Natl Acad Sci U S A. 1985 Feb;82(4):1257–61.

9. Schildge S, Bohrer C, Beck K, Schachtrup C. Isolation and culture of mouse cortical astrocytes. J Vis Exp. 2013 Jan;(71).

10. Wang J, Ito M, Zhong W, Sugita S, Michiue T, Tsuboi T, et al. Observations of intracellular tension dynamics of MC3T3-E1 cells during substrate adhesion using a FRET-based actinin tension sensor. J Biomech Sci Eng. 2016;11(4):16–504.

11. Giordano F, Saheki Y, Idevall-Hagren O, Colombo SF, Pirruccello M, Milosevic I, et al. PI(4,5)P(2)-dependent and Ca(2+)-regulated ER-PM interactions mediated by the extended synaptotagmins. Cell. 2013 Jun;153(7):1494–509.

12. Chang C-L, Hsieh T-S, Yang TT, Rothberg KG, Azizoglu DB, Volk E, et al. Feedback regulation of receptor-induced Ca2+ signaling mediated by E-Syt1 and Nir2 at endoplasmic reticulum-plasma membrane junctions. Cell Rep. 2013 Nov;5(3):813–25.

13. Li H, Korennykh A V, Behrman SL, Walter P. Mammalian endoplasmic reticulum stress sensor IRE1 signals by dynamic clustering. Proc Natl Acad Sci U S A. 2010 Sep;107(37):16113–8.

14. Meng F, Sachs F. Visualizing dynamic cytoplasmic forces with a compliance-matched FRET sensor. J Cell Sci. 2011 Jan;124(Pt 2):261–9.

15. Yang C, Zhang X, Guo Y, Meng F, Sachs F, Guo J. Mechanical dynamics in live cells and fluorescence-based force/tension sensors. Biochim Biophys Acta - Mol Cell Res [Internet]. 2015;1853(8):1889–904. Available from: http://www.sciencedirect.com/science/article/pii/S0167488915001469

16. Williamson P, Mattocks K, Schlegel RA. Merocyanine 540, a fluorescent probe

sensitive to lipid packing. Biochim Biophys Acta - Biomembr [Internet]. 1983;732(2):387–93. Available from: http://www.sciencedirect.com/science/article/pii/000527368390055X

17. Wilson-Ashworth HA, Bahm Q, Erickson J, Shinkle A, Vu MP, Woodbury D, et al. Differential Detection of Phospholipid Fluidity, Order, and Spacing by Fluorescence Spectroscopy of Bis-pyrene, Prodan, Nystatin, and Merocyanine 540. Biophys J [Internet]. 2006;91(11):4091–101. Available from: http://www.sciencedirect.com/science/article/pii/S0006349506721257

18. Stefan CJ, Manford AG, Emr SD. ER-PM connections: sites of information transfer and inter-organelle communication. Curr Opin Cell Biol. 2013 Aug;25(4):434–42.

19. Tufanli O, Telkoparan Akillilar P, Acosta-Alvear D, Kocaturk B, Onat UI, Hamid SM, et al. Targeting IRE1 with small molecules counteracts progression of atherosclerosis. Proc Natl Acad Sci U S A. 2017 Feb;114(8):E1395–404.

20. John S, Sivakumar KC, Mishra R. Extracellular Proton Concentrations Impacts LN229 Glioblastoma Tumour Cell Fate via Differential Modulation of Surface Lipids. Front Oncol [Internet]. 2017 Mar 1;7. Available from: http://journal.frontiersin.org/article/10.3389/fonc.2017.00020/full

Supplementary Section 2 – Figures with Legends

Supplementary figure– Fig. S1

FIG. S1: Schematic representation of experimental procedures followed in the study for quick reference of the various experimental phases

Result 1. **Low pHe acts as a biomechanical force-generating agent on the astrocyte's surface, causing the plasma membrane to compress laterally. The extent of lateral compression acts as a read-out of extracellular proton concentrations.**

Study model: Human astrocytes, validation of important conclusion on freshly isolated mouse cortical astrocytes.

Experiments:

(i) Membrane microviscosity analysis upon physiological and low pH treatments via DPH dye assay.

(ii) Lipid packing order analysis upon physiological and low pH treatments via Laurdan dye assay.

(iii) Plasma membrane lateral compression analysis upon physiological and low pH treatments via FRET-based force probe sensors

(iv) Plasma membrane stiffness direct analysis upon physiological and low pH treatments via Atomic Force Microscopy

Result 2. **Low pHe is sensed by sialic acid containing glycan headgroups on the astrocyte's surface, enabling plasma membrane lateral compression**

Study model: Human astrocytes, validation of important conclusion on freshly isolated mouse cortical astrocytes.

Experiments:

(i) Analysis of the role of surface glycans in pH-dependent plasma membrane biophysical remodelling via detachment force measurement through Atomic Force Microscopy.

(ii) Analysis of the density and organization of surface glycans upon physiological and low pH treatments via SNA lectin surface staining

(iii) Analysis of the specific role of sialic acid glycan moiety presented on the headgroups of lipids and proteins (as it is highly susceptible to protonation in high proton concentrations) in laterally compressing the plasma membrane. The read-out was made via FRET-based force probe sensors in sialic acid shaved and unshaved astrocytes, incubated at different pHs

<u>Result 3.</u> GM3 glycosphingolipid is crucial in sensing low pHe via protonation dependent differential clustering of its sialic acid glycan moiety. GM3, therefore, emerges as a key pH meter of astrocytes. The extent of plasma membrane lateral compression, caused by differential GM3 clustering in different pH microenvironments, acts as a read-out of extracellular proton concentrations.

Study model: Human astrocytes, validation of important conclusion on freshly isolated mouse cortical astrocytes

.

Experiments:

(i) Analysis of the specific role of sialic acid glycan moiety presenting glycosphingolipid, GM3, in generating lateral compression of the plasma membrane as a read-out of extracellular proton concentration. Confirmation of plasma membrane lateral compression due to GM3 clustering in GM3 undepleted and depleted astrocytes coupled with GM3 lipid feeding rescue experiments.

(ii) Analysis of GM3 surface clustered organization at different pHs via anti-GM3 antibody, fluorescent GM3 lipid fed plasma membrane imaging and GM3-GM3 homo-ligation experiments via GM3 lipid Homo-FRET.

(iii) Analysis of GM3 clustered organization in the generation of plasma membrane curvatures at low pH via STED microscopy.

<u>Result 4.</u> Differential GM3 clustering triggers ER-associated IRE1-sXBP1 survival genes at low pHe

Study model: Human astrocytes, validation of important conclusion on freshly isolated mouse cortical astrocytes.

Experiments:

(i) Analysis of GM3 clusters/curvatures interactions with ER-PM contact sites via ER-PM contact site markers and GM3 clusters colocalization.

(ii) Analysis of the role of GM3 clustered force foci in stiffening of ER-PM contact sites and ER membranes via exclusion of membrane fluidity sensing in GM3 undepleted astrocytes vs GM3 depleted astrocytes in low pH microenvironment.

(iii) Analysis of the impact of GM3 clusters and ER-PM contact site interaction as well as ER membrane stiffening on activation of ER stress master sensor IRE1.

(iv) Analysis of the activation of the pro-survival arm of ER stress downstream of IRE1, namely generation of sXBP1 and its lipid biogenesis associated downstream targets SREBP2/ACSS2.

Result 5. Cholesterol is a primary biosynthetic product of GM3 activated IRE1-sXBP1 mechanotransduction enabling survival response machinery at low pHe

Study model: Human astrocytes, validation of important conclusion on freshly isolated mouse cortical astrocytes, in vivo validation in a mouse model of brain acidification

Experiments:**(i)** Validations of the key role of cholesterol biogenesis and its trafficking to the surface via sXBP1-SREBP2-ACSS2 axis and GM3 as an upstream trigger of this process on pro-survivability under acid stress.

(ii) Validation of the indispensability of GM3-IRE1-sXBP1-SREBP2-ACSS2-Cholesterol machinery in the generation of pro-survival adaptation under acidic microenvironment through mouse model of brain acidification and in brain ischaemia patient tissues.

Result 6. Inhibition of splicing activity of IRE1 in combination with depletion of excess surface cholesterol negatively impacts the growth of astrocytes associated tumours. The results show that the same anti-acidosis prosurvival sense-respond machinery is generated by glioblastoma brain tumour cells, as in normal astrocytes, which allows them to thrive and grow in predominantly acidic areas microenvironment of GBM tumours.

Study model: Human glioblastoma patient cell lines, Human glioblastoma patient tissue arrays, Glioblastoma patient sample database curation.

Experiments: **(i)** Validation of the existence of GM3-IRE1-sXBP1-SREBP2-ACSS2-Cholesterol machinery glioblastoma tumour patient's tissue samples and GBM patient-derived cell line via immunohistochemistry/cytochemistry and colocalization studies.

(ii) Analysis of the impact of sXBP1 generation inhibition and simultaneous surface cholesterol quenching via Amphotericin B on the levels of surface cholesterol and in loss of cell impermeability as well as tumour cells growth in GBM tumour cells treated with different pHs. Leakage assays and tumour growth assays were performed.

(iii) Additional experiments performance to validate and concretise the observations that GM3-IRE1-sXBP1-SREBP2-ACSS2-Cholesterol is a key machinery in both astrocytes and astrocytic tumours which effectively enable survival adaptation in acidic microenvironment.

Inhibition of excess cholesterol levels from the GBM cell surface by blocking its biosynthesis via inhibition of sXBP1 and quenching of excess cholesterol already existing on the surface via Amphotericin B presents rationale therapeutics to inhibit tumour cell growth. Conversely, activation of sXBP1 for excess cholesterol biosynthesis and avoidance of surface cholesterol quenching drugs is rationale therapeutics for triggering pro-survival mechanisms in acidosis associated degenerative diseases such as ischaemia.

Supplementary Section 2 – Figures with Legends

Fig. S2 – Fig. S44

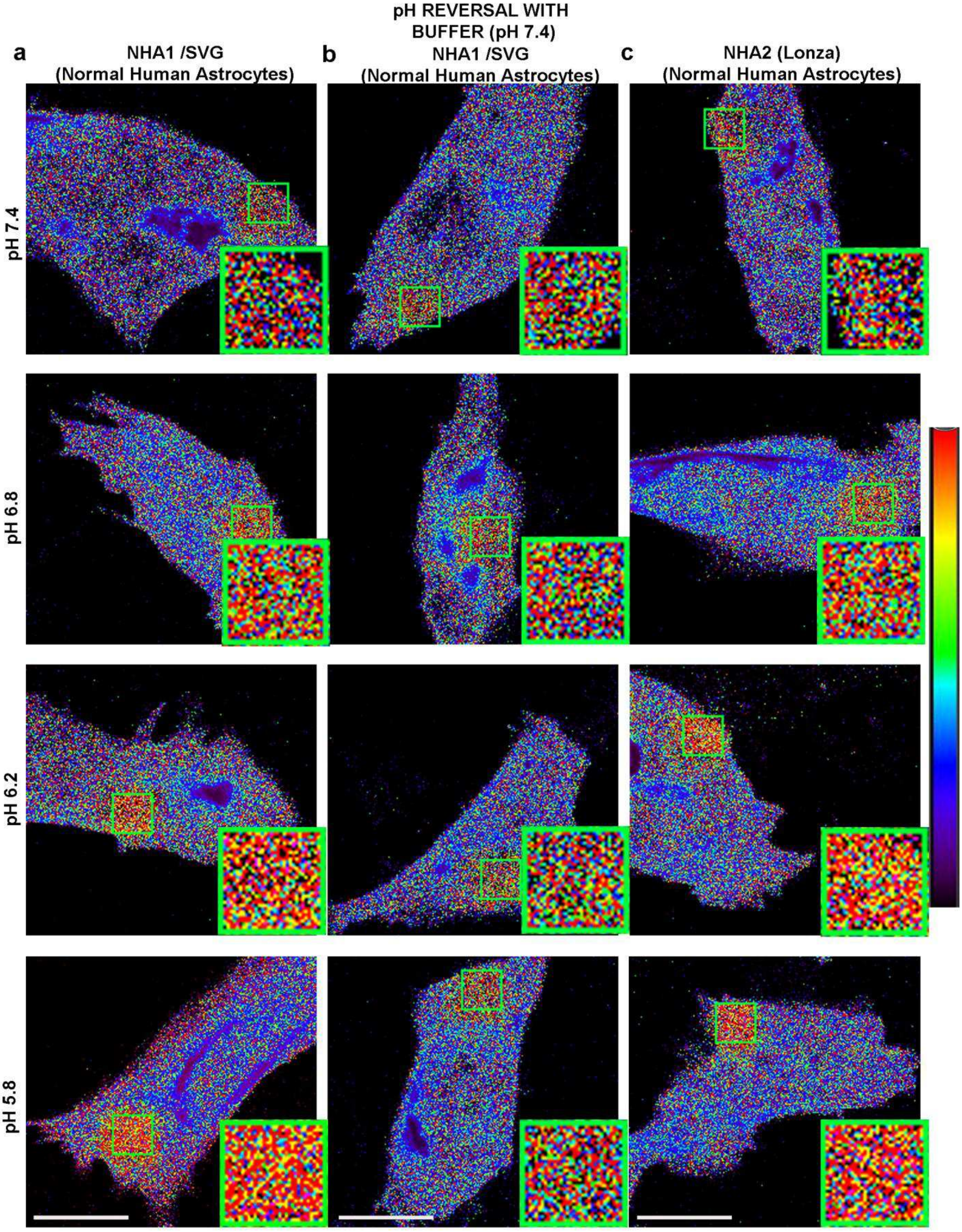
pH REVERSAL WITH BUFFER (pH 7.4)
a
NHA1 /SVG
(Normal Human Astrocytes)
b
NHA1 /SVG
(Normal Human Astrocytes)
c
NHA2 (Lonza)
(Normal Human Astrocytes)
pH 7.4
pH 6.8
pH 6.2
pH 5.8

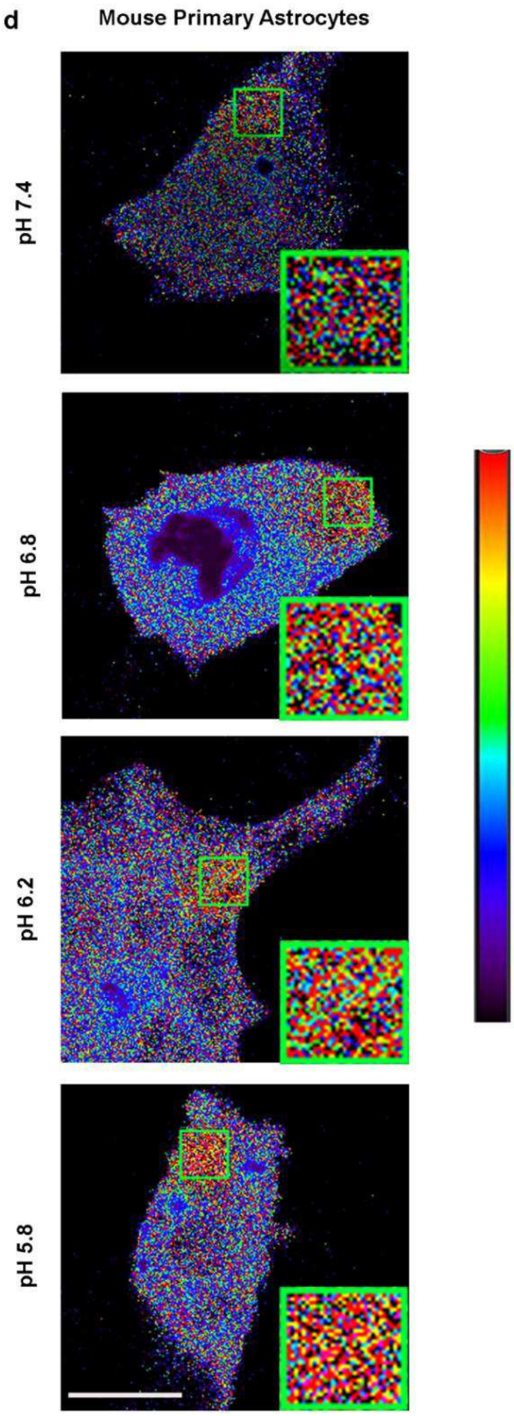

Fig. S2: Representative FRET-based force probe ratiometric images of astrocytes incubated at different pH units. Related to Fig. 1e

FRET-based force probe was transfected in astrocytes (from different sources) and was exposed to different pH units for 4 hrs. Cells were fixed in 1.5% PFA, and FRET images were acquired to calculate the FRET efficiency as well as to obtain the FRET ratiometric images:

(a) Shows representative force probe FRET ratiometric images of NHA1 (SVG) passagable human astrocyte cell line when incubated with different pH units. The ROI area, bleached in the FRET channel, represents the corresponding gain in donor fluorescence in pH treatments. See zoomed image of ROI in inset and notice the increase in FRET ratio in low pH treatments.

(b) Shows representative force probe FRET ratiometric images of NHA1 (SVG) passagable human astrocyte cell line when incubated with different pH units for 4 hrs, media was replaced with the medium at pH 7.4 in all pH treatments. Buffer treatment (medium at pH 7.4) was continued for another 4 hrs before cells were fixed for FRET imaging. The ROI area, bleached in the FRET channel, represents the corresponding gain in donor fluorescence in pH treatments. See zoomed image of ROI in the inset; no significant donor gain (increase in FRET ratio) was noticed in (b) vs (a) due to low pH reversal to physiological pH.

(c) Shows representative force probe FRET ratiometric images of NHA2 (Lonza), primary human astrocyte cells with limited passagibility, when incubated with different pH units. The ROI area, bleached in the FRET channel, represents the corresponding gain in donor fluorescence in pH treatments. See zoomed image of ROI in inset and notice the increase in FRET ratio in low pH treatments.

Rhodamine phalloidin staining for F-actin stress fibres

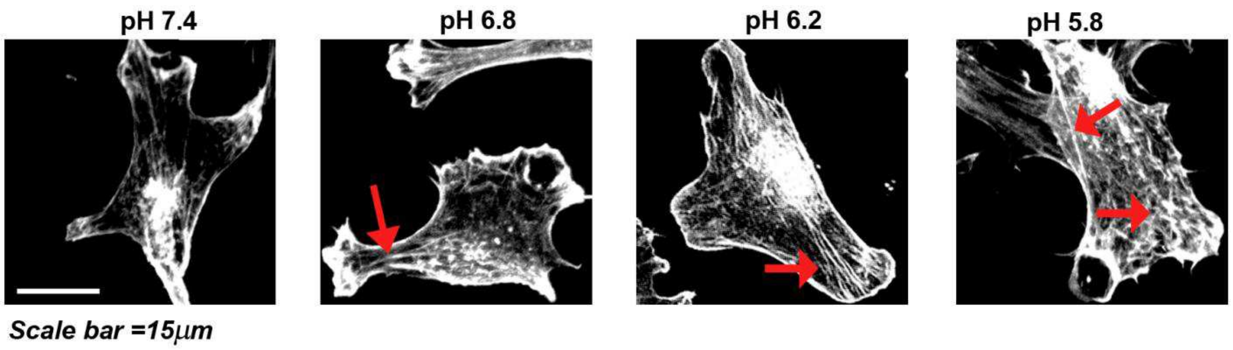

Fig. S3: F-actin stress fibre formation at low pH

Thick and long F-actin stress fibres emanate from the cell surface at lower pH units (see red arrows). Representative images are shown from recordings in at least 20 cells taken in each single cell measurement, in each pH condition, from 3 independent experiments.

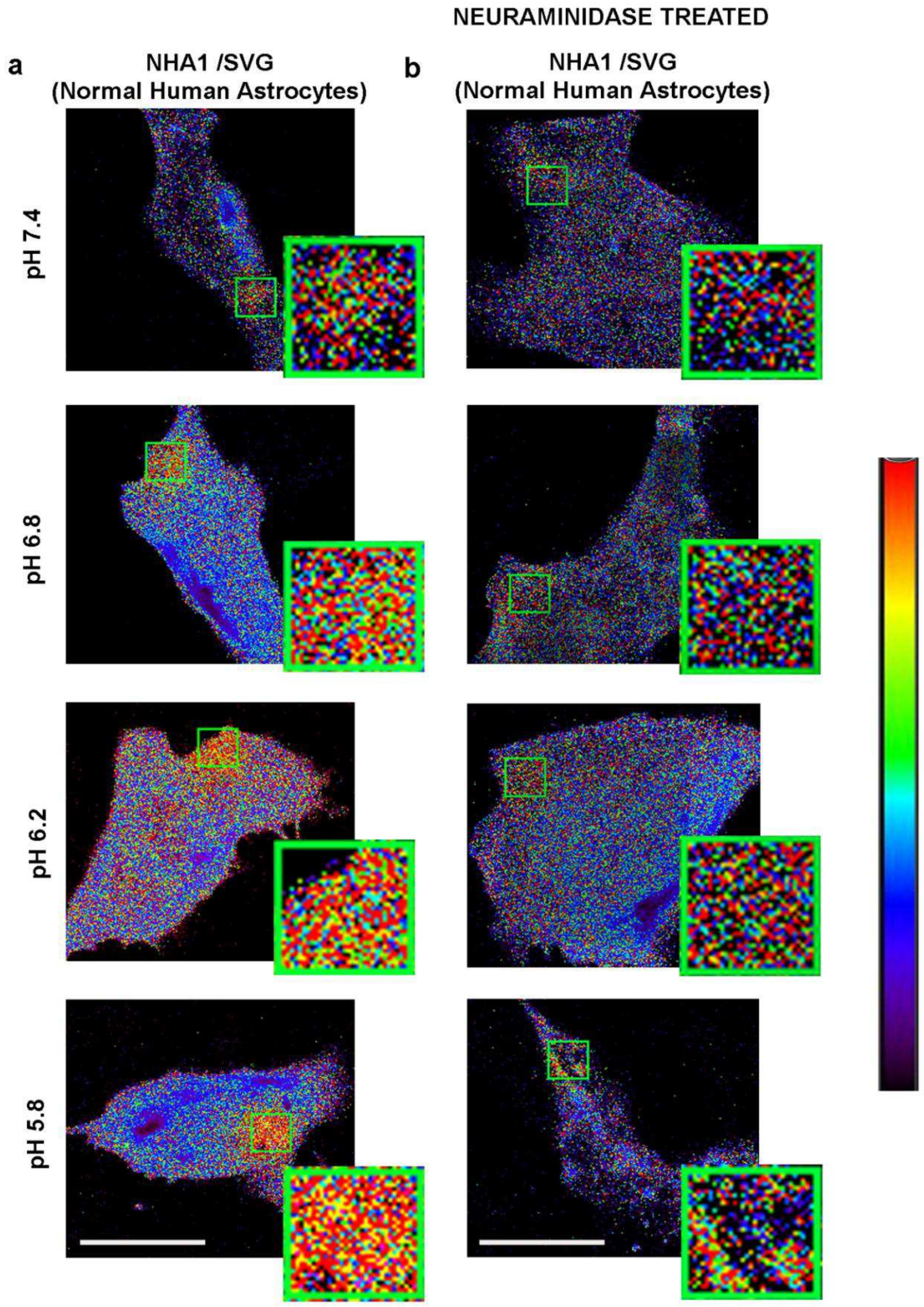

Fig. S4: **Representative force probe FRET ratiometric images of astrocytes treated with neuraminidase and then exposed to different pH units, related to Fig. 2a**

(a-b) Supplementary Fig. shows representative force probe FRET ratiometric images of NHA1 (SVG) passagable human astrocyte cells, treated or not with neuraminidase for 1 hour

at RT to shave surface sialic acid moieties and cells were then exposed to different pH units for 4 hrs.

The ROI area, bleached in the FRET channel, represents the corresponding gain in donor fluorescence in pH treatments. See zoomed image of ROI in the inset; no significant donor gain (increase in FRET ratio) was noticed in (a) vs (b) due to neuraminidase treatments.

Recordings from at least 30 cells were taken for single-cell measurements, in each pH condition, from 3 independent experiments. Image acquisition parameters for each channel were kept the same across each condition and over independent replicates.

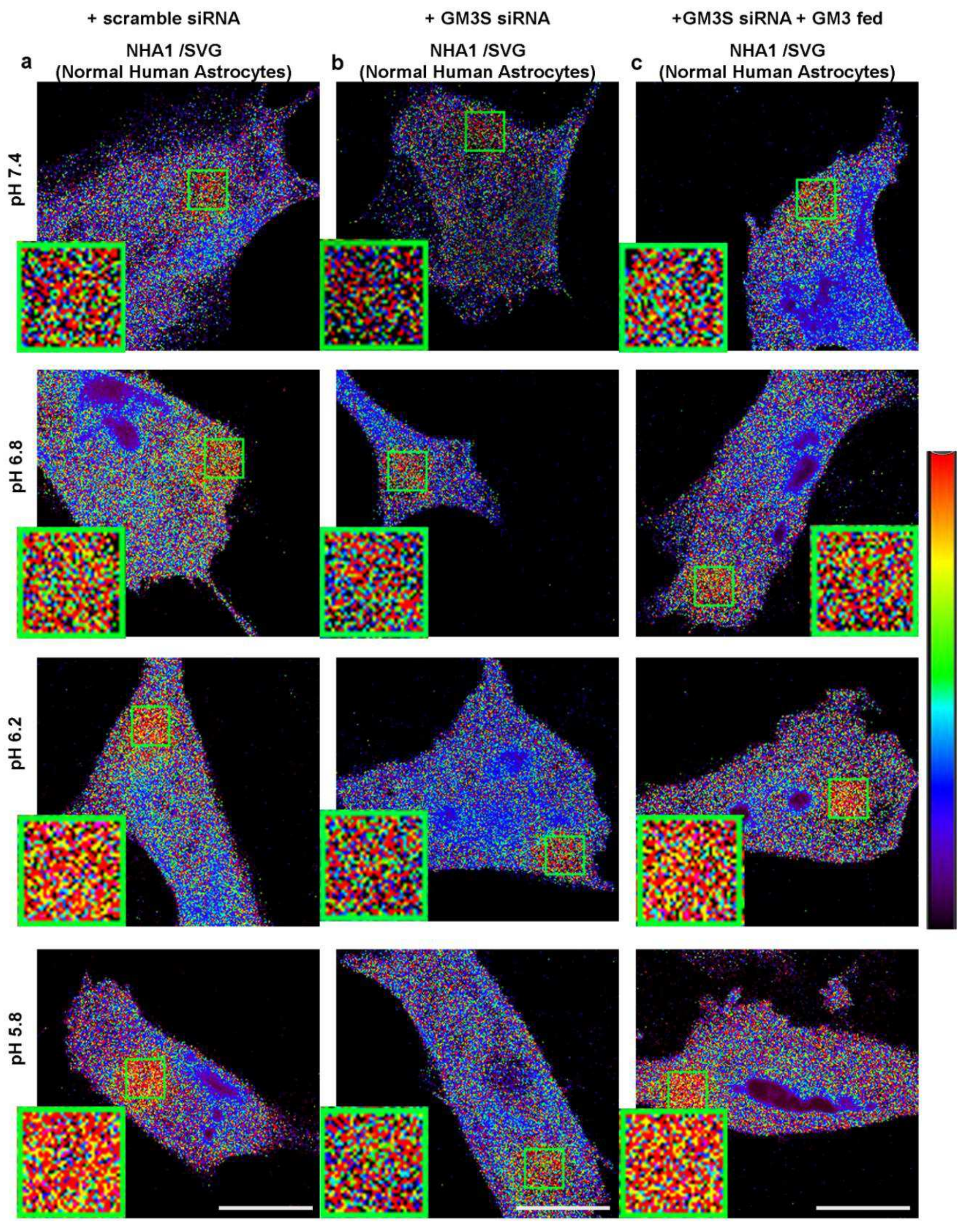

Fig. S5: Representative force probe FRET ratiometric images of astrocytes treated with siRNA of GM3 synthase and when GM3 synthase depleted cells were fed with GM3 lipid and then exposed to different pH units, related to Fig. 2b

(a-c) Supplementary Fig. panels show representative force probe FRET ratiometric images of astrocytes treated with 100pM scrambled siRNA (Mock), 100pM GM3S siRNA or 100pM

GM3S siRNA cells which were externally fed with GM3 lipid (50µM) and then exposed to different pH units. The ROI area, bleached in the FRET channel, represents a corroborating gain in donor fluorescence or not in low pH treatments with either scramble siRNA transfected, GM3S siRNA, or GM3S siRNA transfected astrocytes but were fed with exogenous GM3. See zoomed image of ROI in the inset; no significant donor gain (increase in FRET ratio) was observed in (a) vs (b) due to depletion of GM3 via GM3S siRNA transfection. Whereas in GM3 depleted astrocytes that were priorly fed with GM3 lipid and then exposed to pH treatments, cells showed significant donor gain in low pH values, clearly indicating the low pH mechanosensing role of GM3.

Recordings from at least 30 cells are taken for single-cell measurements, in each pH condition, from 3 independent experiments. Image acquisition parameters for each channel were kept the same across each condition and over independent replicates.

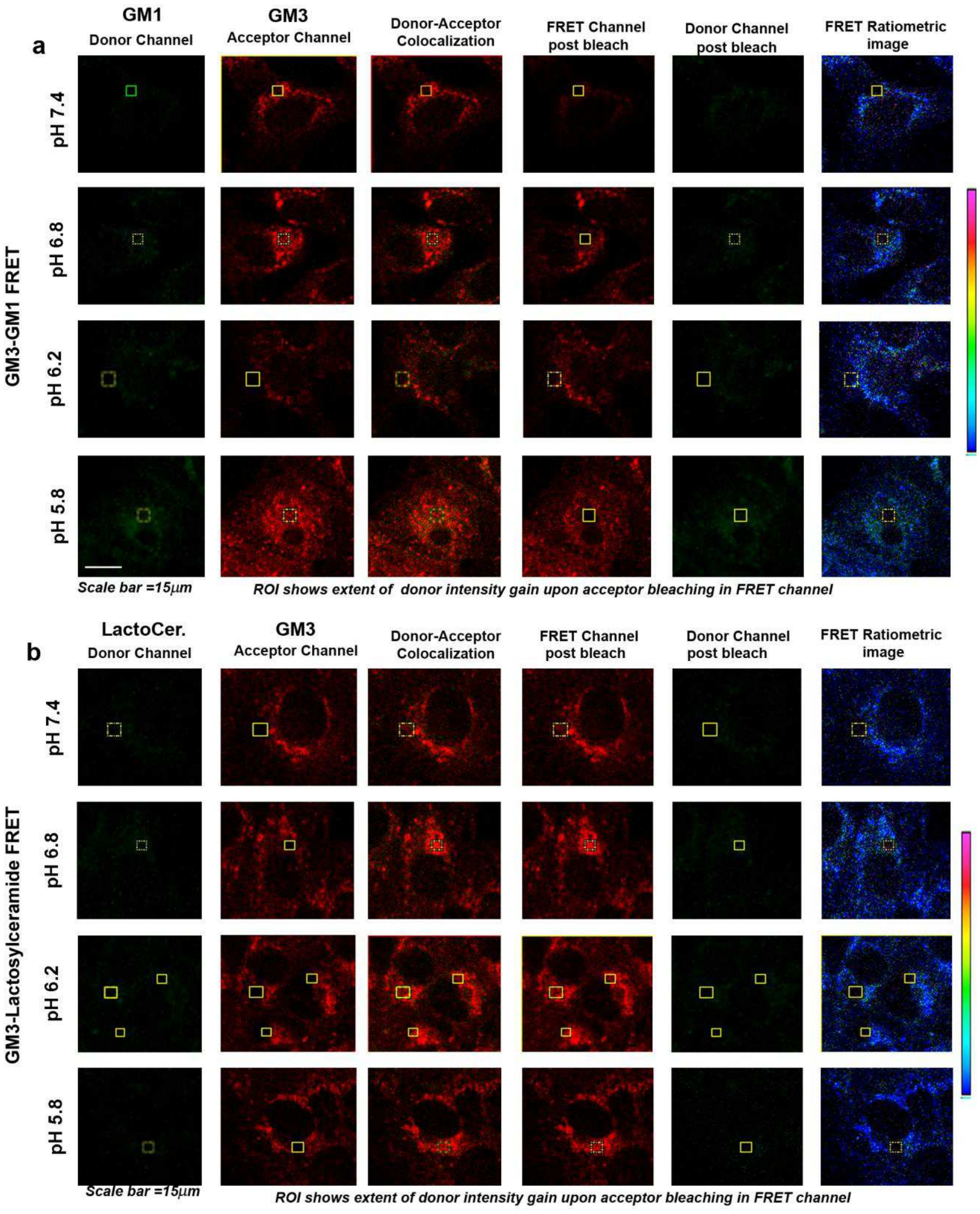

Fig. S6: Representative force probe FRET ratiometric images of astrocytes fed with Bodipy FL GM1 and Rhodamine GM3 or Bodipy FL Lactosylceramide and Rhodamine GM3 and then exposed to different pH units, related to Fig. 2f

(a) Shows representative FRET ratiometric images of astrocytes fed with Bodipy GM1 (3.5µM) and Rhodamine Red-GM3 (3.5µM) and then exposed to different pH units. The ROI area, bleached in the FRET channel, shows a weak donor fluorescence gain in low pH treatments, indicating low FRET efficiency between GM1 and GM3.

(b) Shows representative force probe FRET ratiometric images of astrocytes fed with Bodipy LactCer (3.5µM) and Rhodamine Red-GM3 (3.5µM) and then exposed to different pH units. The ROI area, bleached in the FRET channel, shows a weak donor fluorescence gain in low pH treatments, indicating low FRET efficiency between LactCer and GM3. Also, note that LactCer and GM1 do not significantly surface cluster on astrocytes exposed to low pHe values.

Recordings from at least 30 cells are taken for single-cell measurements, in each pH condition, from 3 independent experiments. Image acquisition parameters for each channel were kept the same across each condition and over independent replicates.

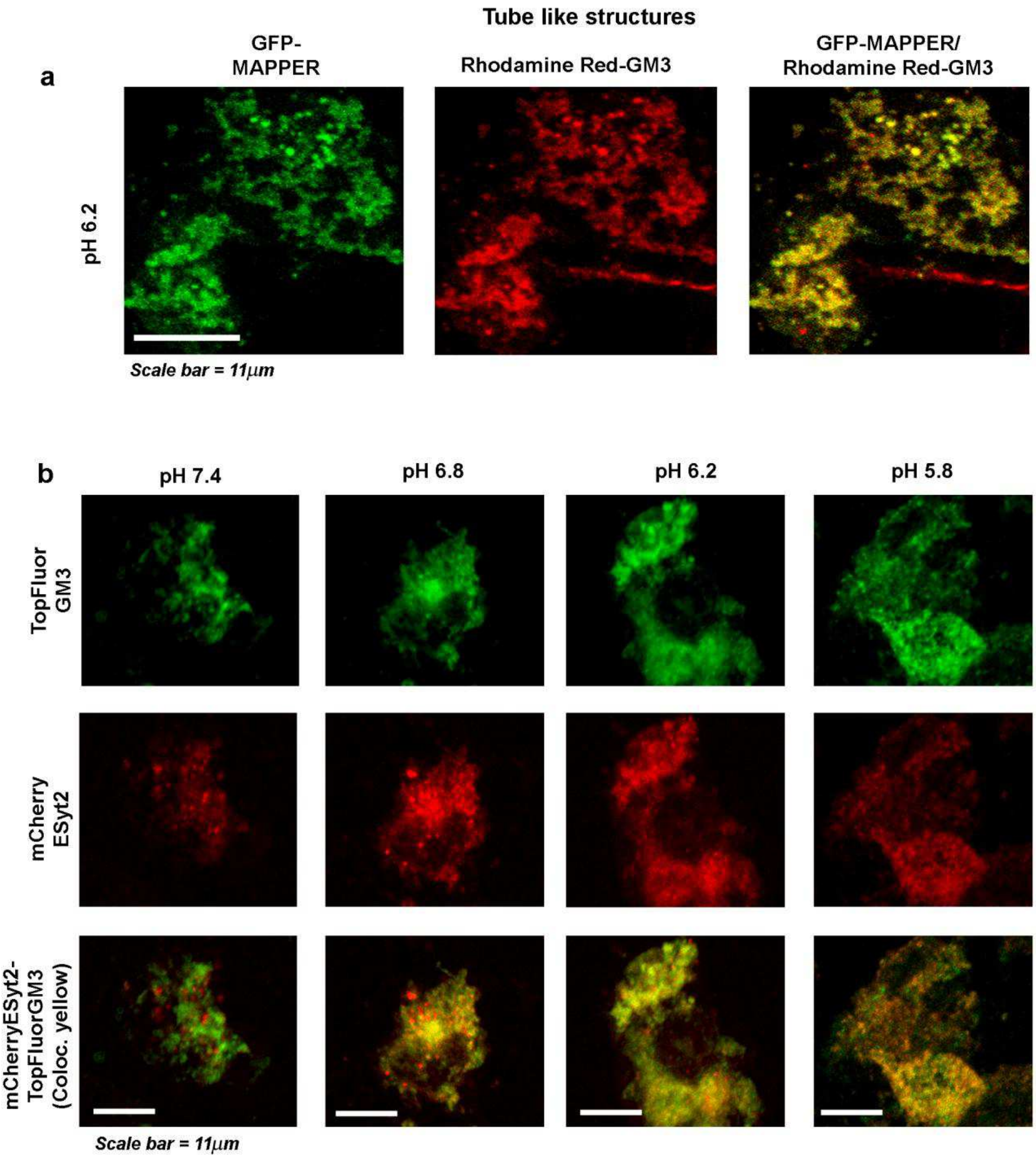

Fig. S7: Representative images of the colocalization of ER-PM markers, GFP-MAPPER and E-Syt2 with GM3 lipid, related to Fig. 3a

(a) GFP-MAPPER and GM3 lipid (detected through anti-GM3 antibody) were observed to be colocalized in tube-like and punctate structures when astrocytes were exposed to low pH units. Image recordings from at least 30 cells are taken for single-cell measurements, in each pH condition, from 3 independent experiments.

(b) Astrocytes were transfected with mCherry tagged E-Syt2 plasmid and were fed with fluorescently labelled GM3 (TopFluor GM3, 3.5µM, green) for 2 hrs. The astrocytes were subjected to pH treatments for 4 hrs and fixed with 1.5% PFA. Sequential images of mCherry tagged E-Syt2 and TopFluor GM3 in each condition were acquired through confocal microscopy.

Image acquisition conditions in each channel were kept the same in each condition overall independent experiment. At least 30 cells from random fields were taken for single-cell measurements, in each pH condition, from 3 independent experiments. Colocalization was analyzed in Fiji software.

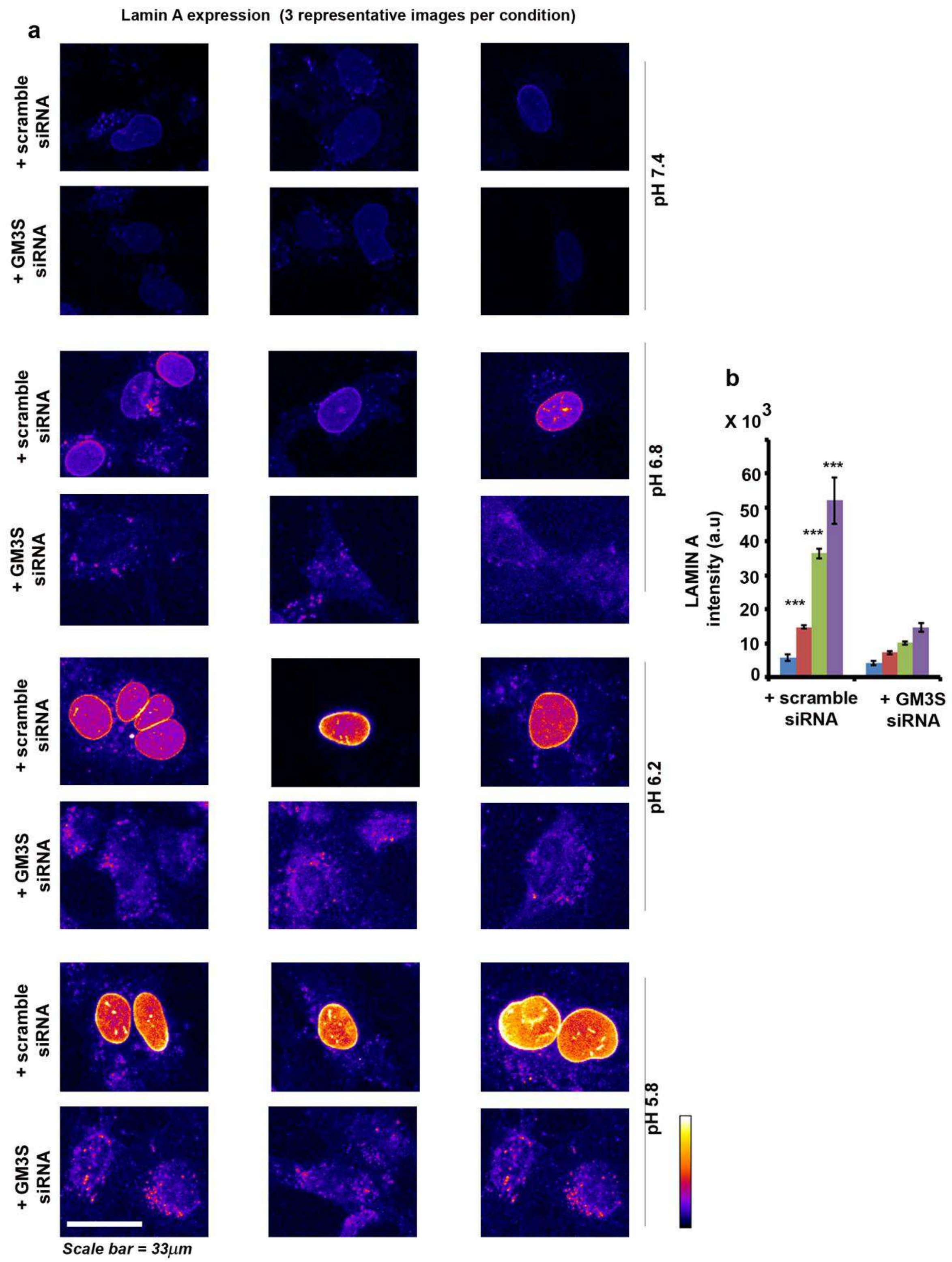

Fig. S8: Nuclear Lamin A expression in scramble siRNA transfected or GM3S siRNA transfected astrocytes upon exposure to different pH units.

Astrocytes were transfected with m-Cherry tagged Lamin A and transfected with scrambled siRNA (Mock) or GM3S siRNA for 36 hrs. Post transfection, astrocytes were given pH treatments for 4 hrs, and cells were fixed in 1.5% PFA.

(a) Shows representative images of mCherry Lamin A in different pH treatments with or without siRNA GM3S. Imaging of mCherry Lamin A signal in each condition was performed in a confocal microscope.

(b) mCherry Lamin A nuclear envelope intensity in at least 30 cells from random fields were taken for single-cell measurements, in each pH condition, with or without siRNA GM3S. Nuclear expression was analyzed in Fiji software.

All datasets are reported as mean± SD. Significance is shown as *p<0.05, **p<0.01, ***p<0.001. Mean is derived from 3 independent experiments. Image acquisition parameters were kept the same across each condition and over independent replicates.

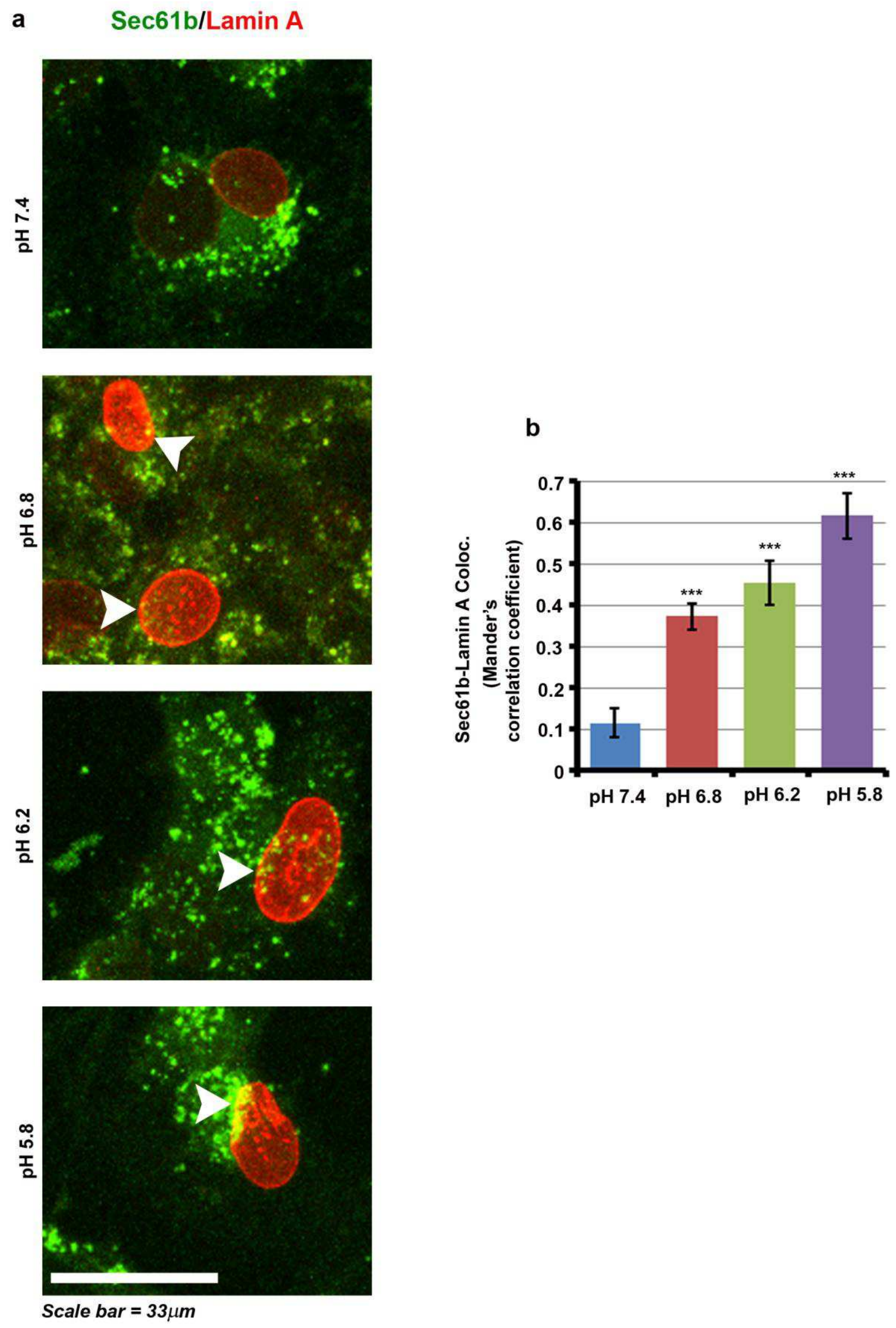

Fig. S9: Lamin A and Sec61b colocalization upon exposure to different pH units.

Astrocytes were transfected with m-Cherry tagged Lamin A and EGFP-Sec61b (ER membrane marker) for 36 hrs. Post transfection, astrocytes were given pH treatments for 4hrs and cells were fixed in 1.5% PFA.

(a) Shows representative images of mCherry Lamin A and EGFP-Sec61b in different pH treatments. Imaging of mCherry Lamin A and EGFP-Sec61b signal in each condition were performed in a confocal microscope. Image acquisition parameters for each channel were kept the same in each condition overall independent experiment. White arrowheads show colocalization in low pH treatments.

(b) mCherry Lamin A and EGFP-Sec61b signal colocalization in different pH treatments are analyzed in Fiji software and plotted graphically.

All datasets are reported as mean± SD. Significance is shown as *p<0.05, **p<0.01, ***p<0.001. Mean is derived from 3 independent experiments. Recordings from at least 30 cells were taken in single-cell measurements, in each pH condition, from 3 independent experiments.

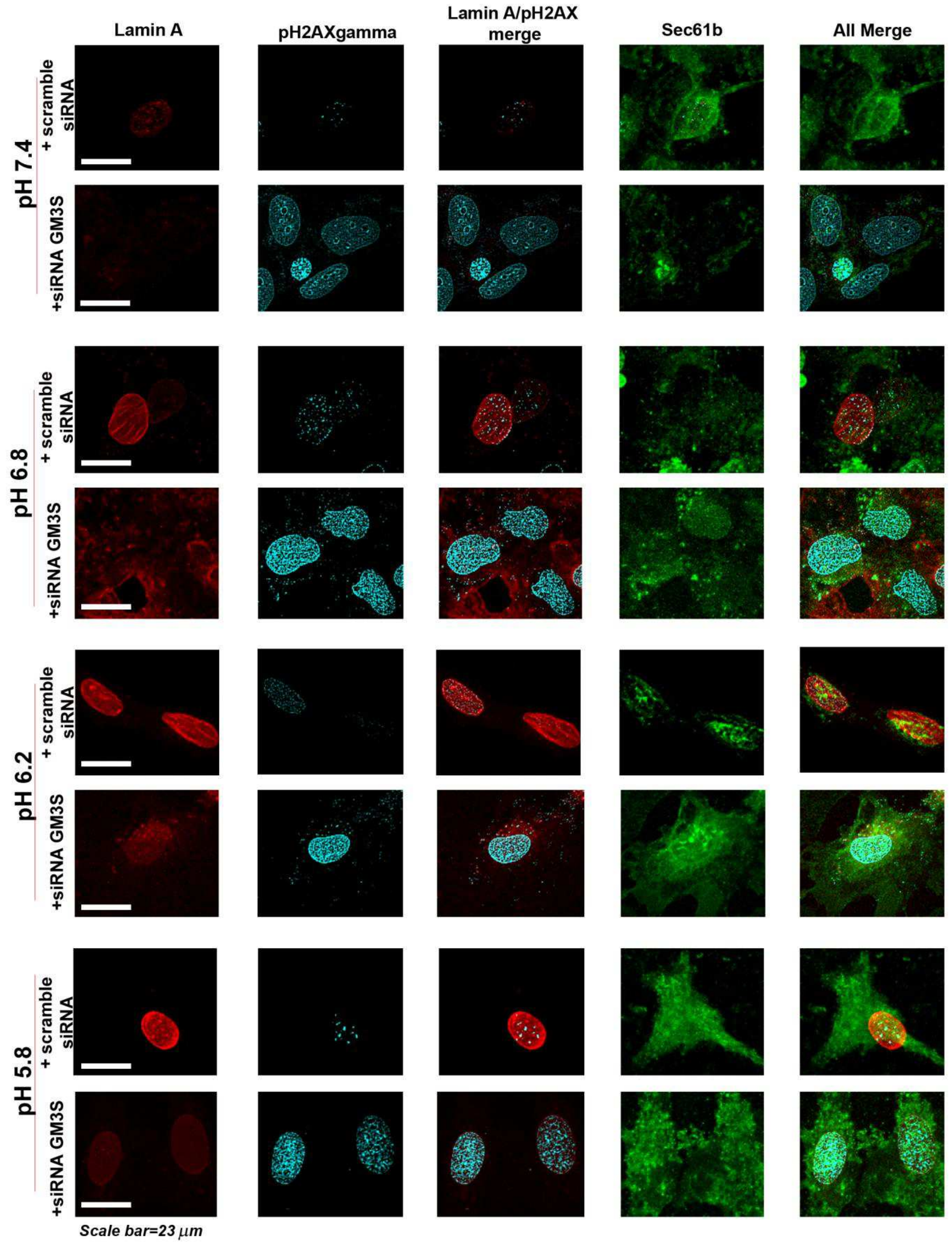

Fig. S10: Nuclear γH2AX levels in mock siRNA transfected or siRNA GM3S transfected astrocytes exposed to different pH units

Astrocytes were transfected with m-Cherry tagged Lamin A and EGFP-Sec61b and were further transfected with either scrambled siRNA (Mock) or GM3S siRNA for 36 hrs. After

transfection, astrocytes were given pH treatments for 4 hours, and cells were fixed in 1.5% PFA. Post fixation, astrocytes were immunostained with γH2AX (phospho S139) to reveal DNA damage foci.

Representative images of m-Cherry tagged Lamin A, EGFP-Sec61b and γH2AX (phospho S139) expression in each pH condition with or without siRNA GM3S is shown. Graphical representation of γH2AX (phospho S139) is shown in Fig. S11.

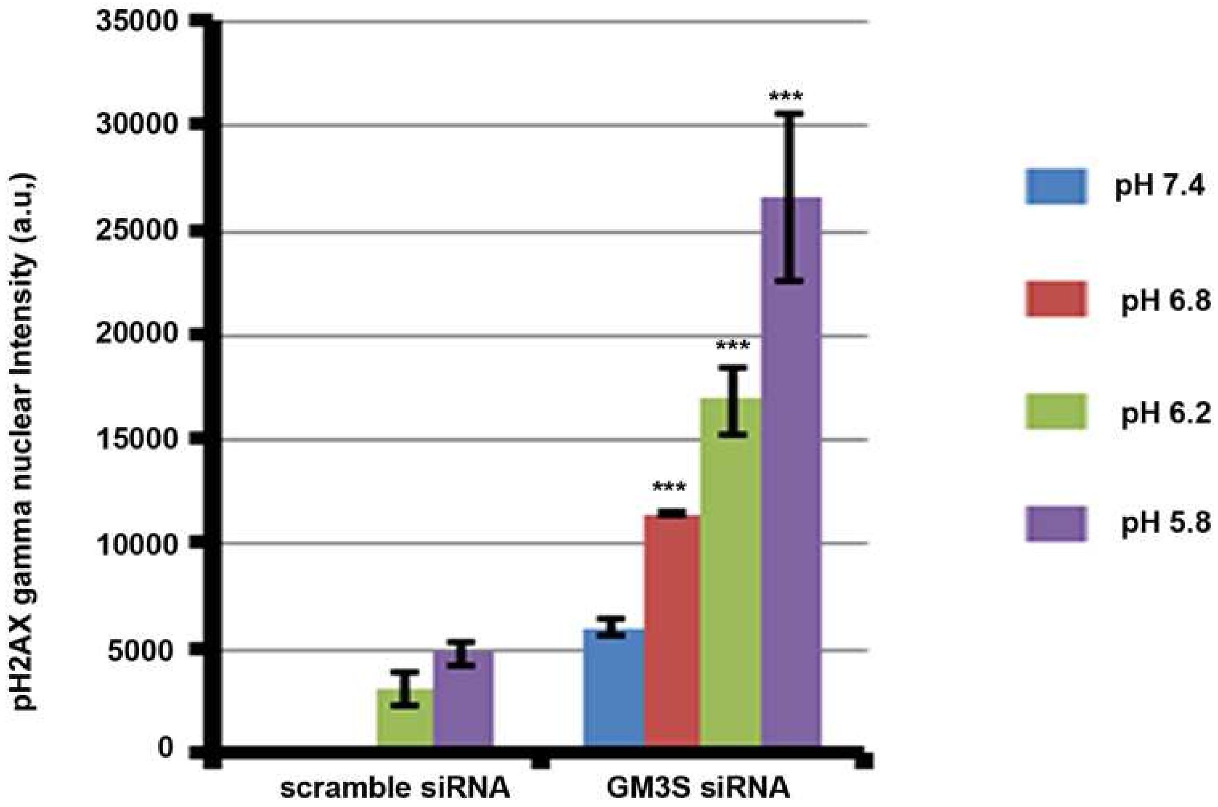

Fig. S11: Quantitation of nuclear γH2AX levels in mock siRNA transfected or siRNA GM3S transfected astrocytes exposed to different pH units

Astrocytes were transfected with m-Cherry tagged Lamin A and EGFP-Sec61b and were further transfected with either scrambled siRNA (Mock) or GM3S siRNA for 36 hrs. After transfection, astrocytes were given pH treatments for 4 hours, and cells were fixed in 1.5% PFA. Post fixation, astrocytes were immunostained with γH2AX (phospho S139) to reveal DNA damage foci.

γH2AX (phospho S139) nuclear intensity in at least 30 cells from random fields was taken for single-cell measurements, in each pH condition, with or without siRNA GM3S. Nuclear expression was analyzed in Fiji software. All datasets are reported as mean± SD. Significance is shown as *p<0.05, **p<0.01, ***p<0.001. Mean is derived from 3 independent experiments.

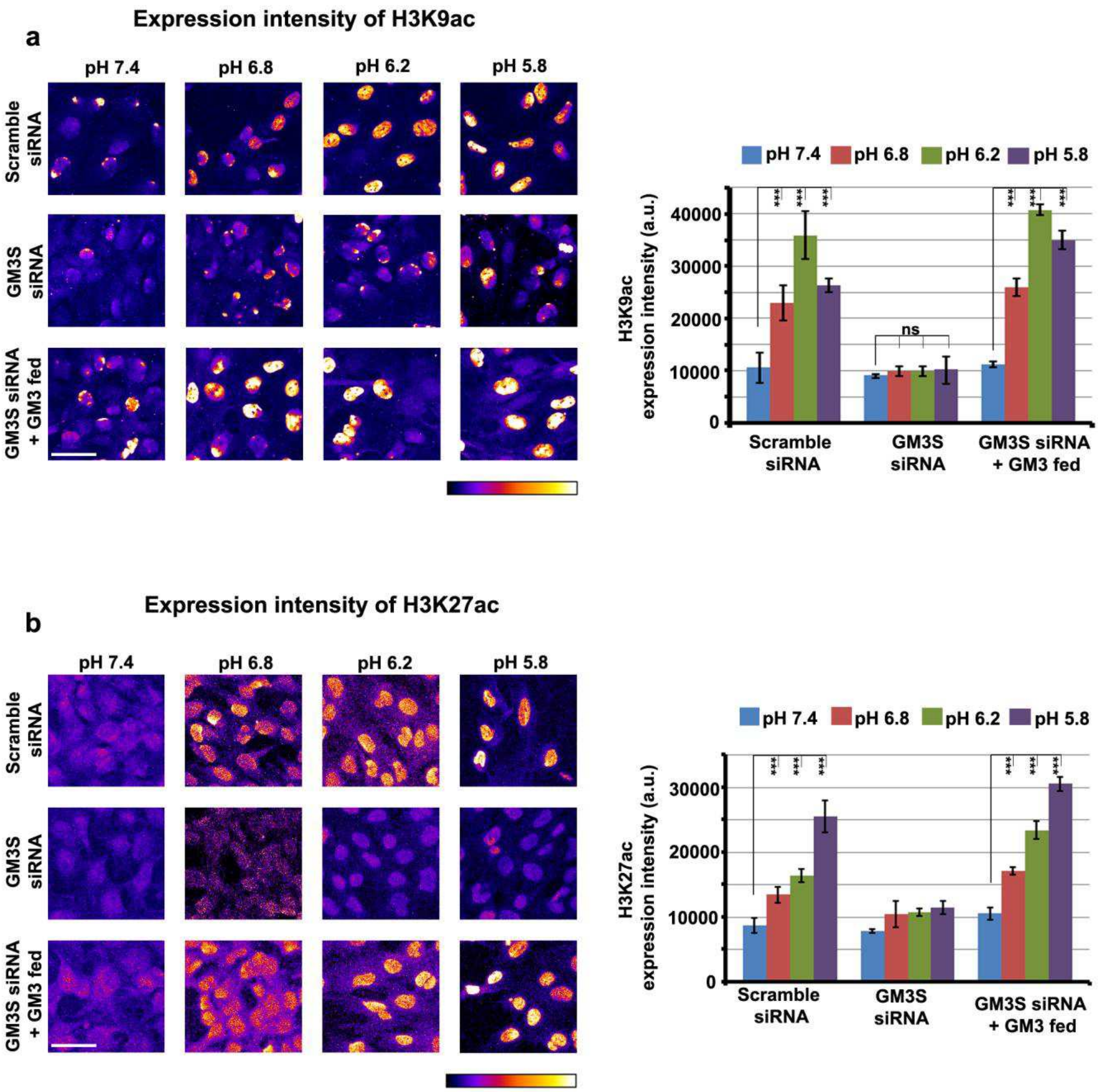

Fig. S12: Astrocytes exposed to low pH show enhanced nuclear localization of H3K9Ac and H3K27Ac, epigenetic activators of transcription, in the presence of GM3

(a-b) Astrocytes (SVG/NHA1), treated with either (i) 100pM scrambled siRNA (Mock) or (ii) 100pM GM3S siRNA or (iii) 100pM GM3S siRNA cells which were externally fed with GM3 lipid (50µM), were further exposed to different pH units for 4 hrs. Post incubation, cells were fixed in 1.5% PFA, washed in 1X PBS, permeabilized with 0.25% saponin and incubated with anti-H3K9Ac and anti-H3K27ac antibody for single immuno-labelling at 4°C for 16 hrs, in independent sets of experiments. The signal was developed with AlexaFluor594 conjugated antibody.

In low pH treated GM3 depleted astrocytes, H3K9ac and H3K27ac were not found to increase. However, GM3 depleted astrocytes fed priorly with GM3 lipid showed a significant rise in H3K9ac and H3K27ac levels.

Imaging of H3K9ac and H3K27ac in each condition was performed in a confocal microscope. Image acquisition parameters for each antigen were kept the same in each condition overall independent experiments. At least 50 cells from random fields were taken for single-cell measurements, in each pH condition, from 3 independent experiments. Nuclear expression was analyzed in Fiji software. Images are represented as fire LUT pseudocolour images with intensity calibration bar for better representation of expression differences in different conditions.

All datasets are reported as mean± SD. Significance is shown as *p<0.05, **p<0.01, ***p<0.001. Mean is derived from 3 independent experiments. Image acquisition parameters were kept the same across each condition and over independent replicates.

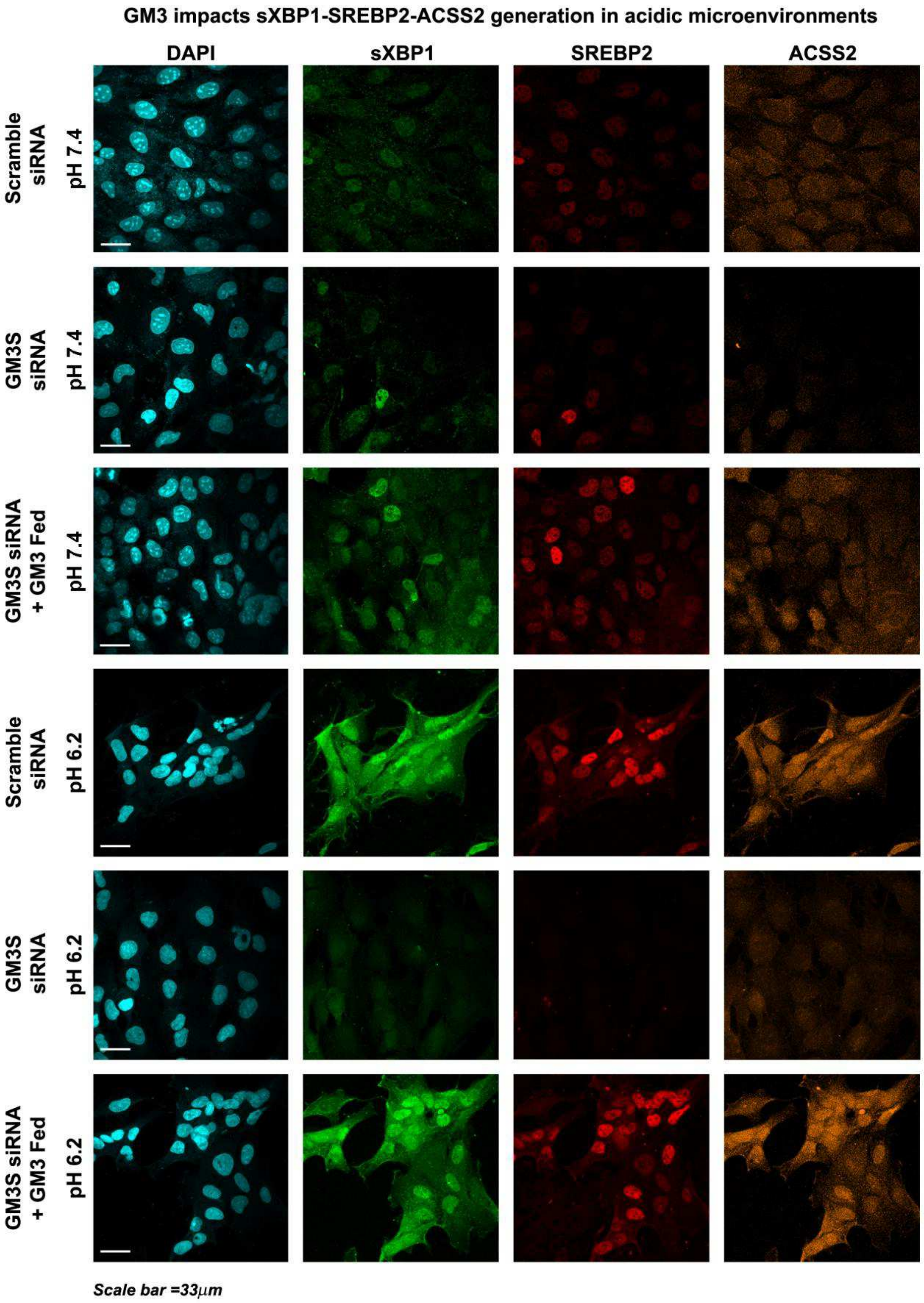

Fig. S13: GM3 enhances co-expression of sXBP1-SREBP2-ACSS2 lipogenesis axis in low pH microenvironment of astrocytes:

Astrocytes (SVG/NHA1), treated with either (i) 100pM scrambled siRNA (Mock) or (ii) 100pM GM3S siRNA or (iii) 100pM GM3S siRNA cells which were externally fed with

GM3 lipid (50µM), were further exposed to different pH units for 8 hrs. Post incubation, cells were fixed in 1.5% PFA, washed in 1X PBS, permeabilized with 0.25% saponin and incubated with anti-sXBP1, anti-SREBP2 and anti-ACSS2 antibodies for triple immuno-labelling at 4°C for 16 hrs, in independent sets of experiments.

Low pHe incubated astrocytes showed nuclear upregulation and co-localization of sXBP1-SREBP2-ACSS2, only in the presence of GM3 enriched conditions.

Imaging for each antigen was performed in a confocal microscope. Image acquisition parameters for each antigen were kept the same in each condition over independent experiments.

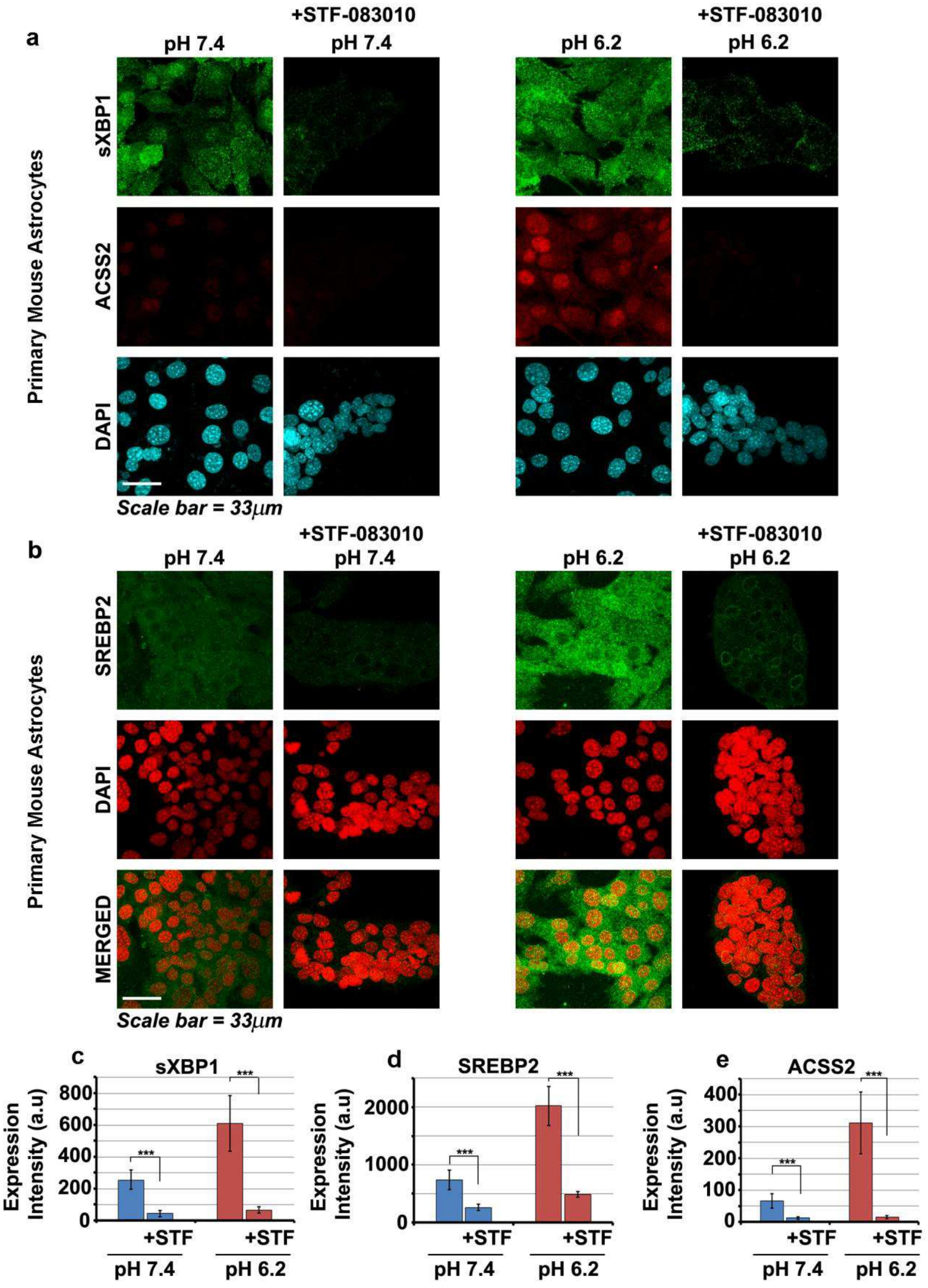

Fig. S14: sXBP1 is required for significant co-expression of SREBP2-ACSS2 lipogenesis axis in low pH microenvironment of mouse primary astrocytes:

(a,b) Mouse primary astrocytes were treated with sXBP1 inhibitor (STF-083010) or not for 2 hours. Astrocytes were further incubated with physiological and low pH media for 8hrs (as sXBP1 mRNA transcript is determined to be upregulated within 6-8 hrs). STF-083010 was

also added in the medium during pH treatments. Post incubation, cells were fixed with 1.5% PFA, washed in 1X PBS, permeabilized with 0.25% saponin and incubated with anti-sXBP1 and anti-ACSS2 antibodies for double immunolabelling at $4^{o}C$ for 16 hrs. The sXBP1 signal was developed with AlexaFluor488 conjugated secondary antibody, and ACSS2 was developed by AlexaFluor 594 conjugated antibody. Single immunolabelling with anti-SREBP2 was also performed.

Imaging of sXBP1, SREBP2 and ACSS2 in each condition were performed in a confocal microscope. Image acquisition conditions in each channel were kept the same in each condition over independent experiments. At least 50-70 cells from random fields were taken for single-cell measurements, in each pH condition, from 3 independent experiments.

Nuclearly localized expression of each antigen was analyzed in Fiji software.

(c-e) Analysis shows that inhibition of sXBP1 generation led to significantly low lipogenesis-associated transcription factors, namely SREBP2-ACSS2. All datasets are reported as mean± SD. Significance is shown as *$p<0.05$, **$p<0.01$, ***$p<0.001$. Mean is derived from 3 independent experiments.

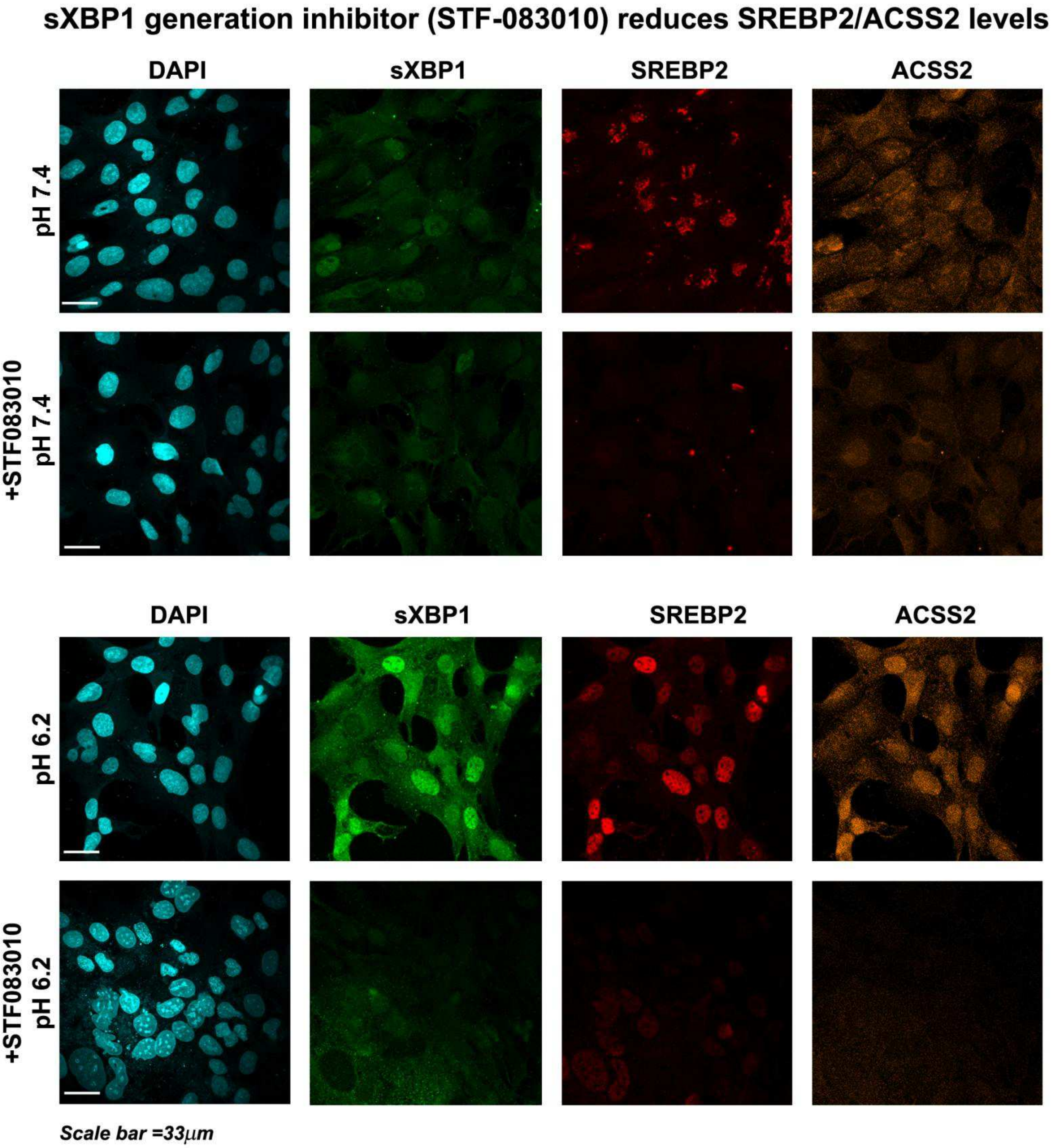

Fig. S15: sXBP1 is required for significant co-expression of SREBP2-ACSS2 lipogenesis axis in low pH microenvironment of human astrocytes:

Human astrocytes were treated either with sXBP1 inhibitor (STF-083010) or not for 2 hours. Astrocytes were further incubated with physiological and low pH media for 8hrs (as sXBP1 mRNA transcript is determined to be upregulated within 6-8 hrs). STF-083010 was also added in the medium during pH treatments. Post incubation, cells were fixed in 1.5% PFA,

washed in 1X PBS, permeabilized with 0.25% saponin and incubated with anti-sXBP1 and anti-ACSS2 antibodies for double immunolabelling at 4°C for 16 hrs. The sXBP1 signal was developed with AlexaFluor488 conjugated secondary antibody, ACSS2 was developed by AlexaFluor647 conjugated antibody, and SREBP2 signal was developed with AlexaFluor 596 conjugated secondary antibody.

Sequential imaging of sXBP1, SREBP2 and ACSS2 in each condition were performed in a confocal microscope. Image acquisition conditions in each channel were kept the same in each condition over independent experiments. Data images clearly show that inhibition of sXBP1 generation led to significantly low levels of lipogenesis associated transcription factors, namely SREBP2-ACSS2.

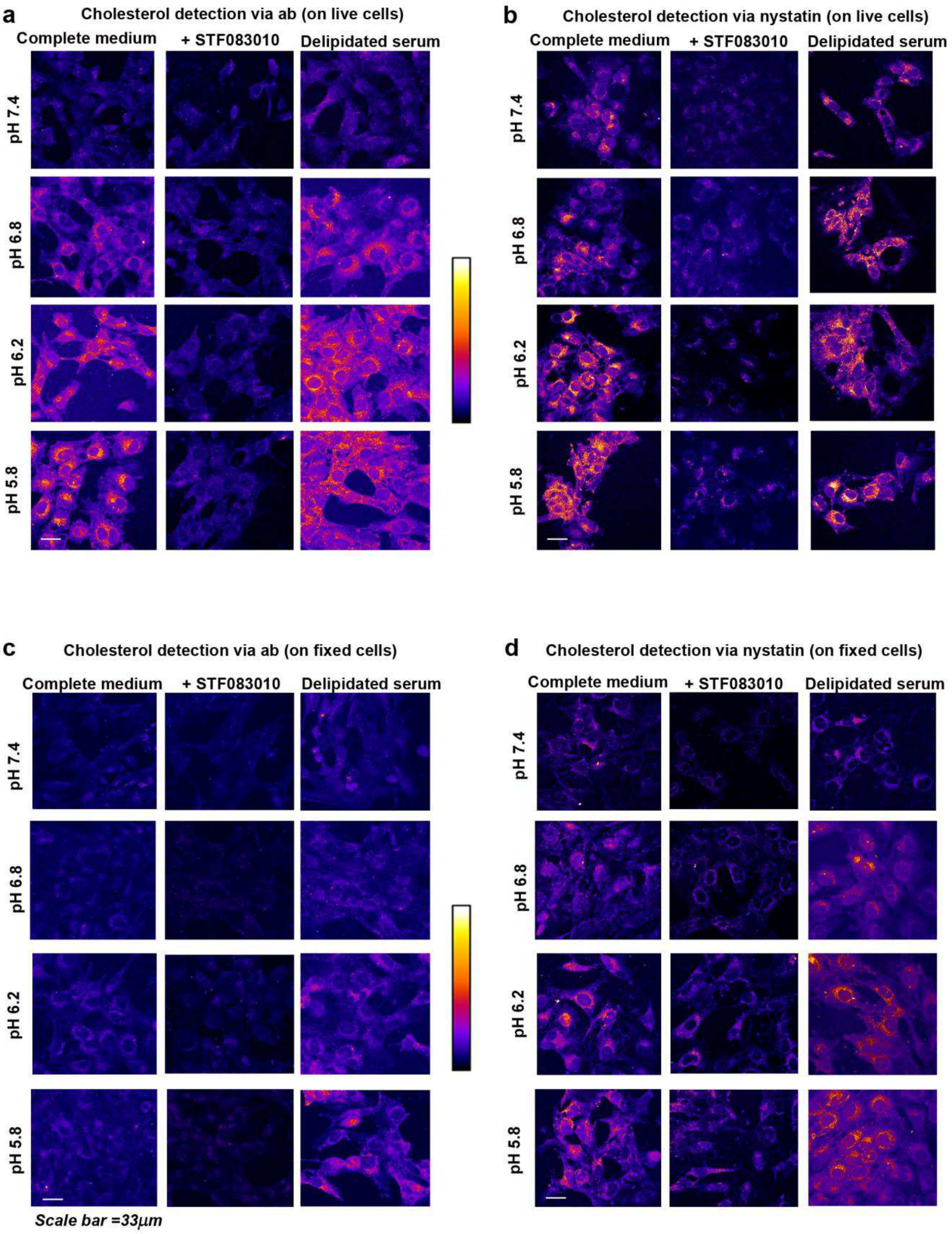

Fig. S16: Representative images of astrocytes' surface cholesterol levels in low pH units with or without STF-083010 treatment and when pH treatments were given in delipidated media, related to Fig. 4h.

The experiment was performed in four independent sets with 3 biological replicates each for detection of surface cholesterol through anti-cholesterol antibody and nystatin on live and fixed astrocytes.

(i) SVG-astrocytes were given pH treatments for 6 hrs in a complete medium containing 10% FBS.

(ii) For inhibiting cholesterol synthesis via the sXBP1 axis, astrocytes were first treated with STF-083010 (60µM) for 6 hrs before pH treatment. Cells were then given pH treatment with STF-083010 in the medium for 6 hrs.

(iii) For experiments in delipidated serum, astrocytes were incubated with a medium containing 10% delipidated serum for 2 hrs prior to pH treatment; post this, pH treatments were given for the next 6 hrs.

(a) Post pH treatments, live astrocytes in described experimental set-ups (i-iii) were transferred at 4°C (cold room, on ice) and were surface incubated with the anti-cholesterol antibody for 1 hr in the cold to prevent endocytosis. The cells were first washed with cold 1X PBS buffer to remove unbound antibodies and then fixed with 1.5% PFA. The surface was blocked to inhibit non-specific signal development. Surface cholesterol signal was developed with AlexaFluor 594 conjugated secondary antibody, incubated on the surface for 1hr at RT.

(b) Post pH treatments, live astrocytes in described experimental set-ups (i-iii) were transferred at 4°C (cold room, on ice) and were surface incubated with 50µg/ml of nystatin for 40 min. The surface was washed with cold 1X PBS, and the cells were fixed with 1.5% cold PFA. Slides were consecutively washed thoroughly with 1X PBS and then mounted with 70% glycerol/PBS.

(c) Post pH treatments, astrocytes in described experimental set-ups (i-iii) were fixed with 1.5% PFA at RT. Cells were first blocked with 5% BSA and 2% normal donkey serum solution, and then the surface was incubated with an anti-cholesterol antibody for 1 hr. Surface cholesterol signal was developed with AlexaFluor 594 conjugated secondary antibody, incubated on the cell surface for 1hr at RT.

(d) Post pH treatments, astrocytes in described experimental set-ups (i-iii) were fixed with 1.5% PFA at RT. Astrocyte surface was incubated with 50µg/ml of nystatin for 40 min. The surface was washed with 1X PBS, and cells were mounted in 70% glycerol/PBS.

Imaging of anti-cholesterol antibody and nystatin signal in each condition was performed via confocal microscopy. Image acquisition parameters were kept the same in each condition over independent experiments. At least 60 cells from random fields were taken for single-cell measurements, in each pH condition, from 3 independent experiments. Expressions were analyzed in Fiji software. For better representation of Intensity differences in the image pixels, images were converted into pseudocolour fire LUT images. The calibration bar indicates low to high pixel intensities.

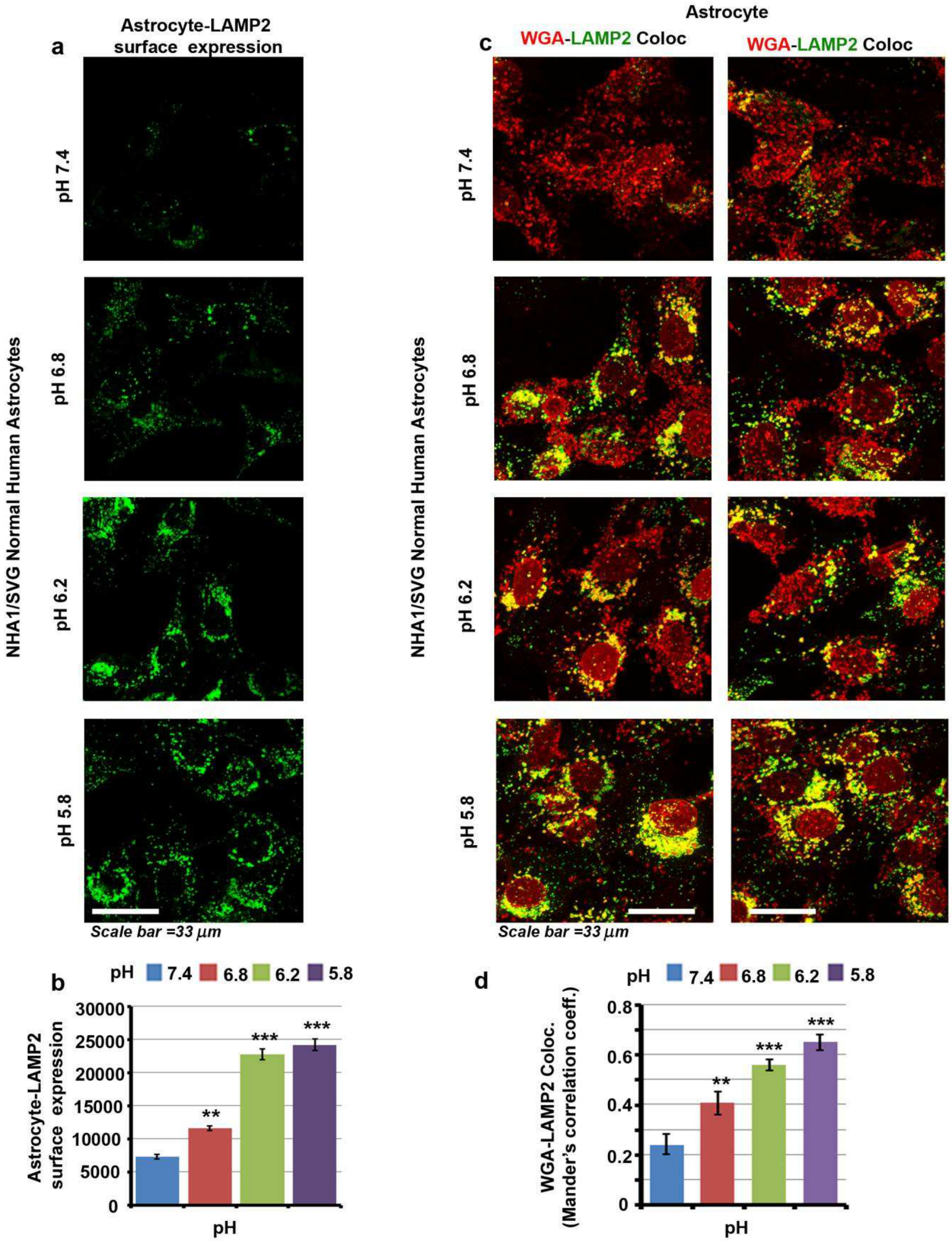

Fig. S17: Surface expression and colocalization of LAMP2 with plasma membrane marker WGA in astrocytes exposed to different pH units.

Astrocyte cell lines (SVG) was incubated in different pH conditions for 4 hrs. Post incubation, cells were fixed in 1.5% PFA, washed in 1X PBS and incubated with the anti-LAMP2 primary antibody on the cell surface (without permeabilization step) for 16 hrs at

4°C. The surface LAMP2 signal was developed with AlexaFluor 488 conjugated secondary antibody. After this, WGA conjugated with AlexaFluor 594, a cell surface marker, was incubated on cells for 45 minutes. The cells were washed and mounted in 70% glycerol/PBS mountant. Sequential imaging of surface LAMP2 and WGA signals were performed in each condition via confocal microscopy. Image acquisition conditions in each channel were kept the same in each condition over independent experiments. At least 60 cells from random fields were taken in single-cell measurements, in each pH condition, from 3 independent experiments. **(a-b)** Expression and **(c-d)** colocalization of LAMP2 with WGA as surface marker were analyzed in Fiji software.

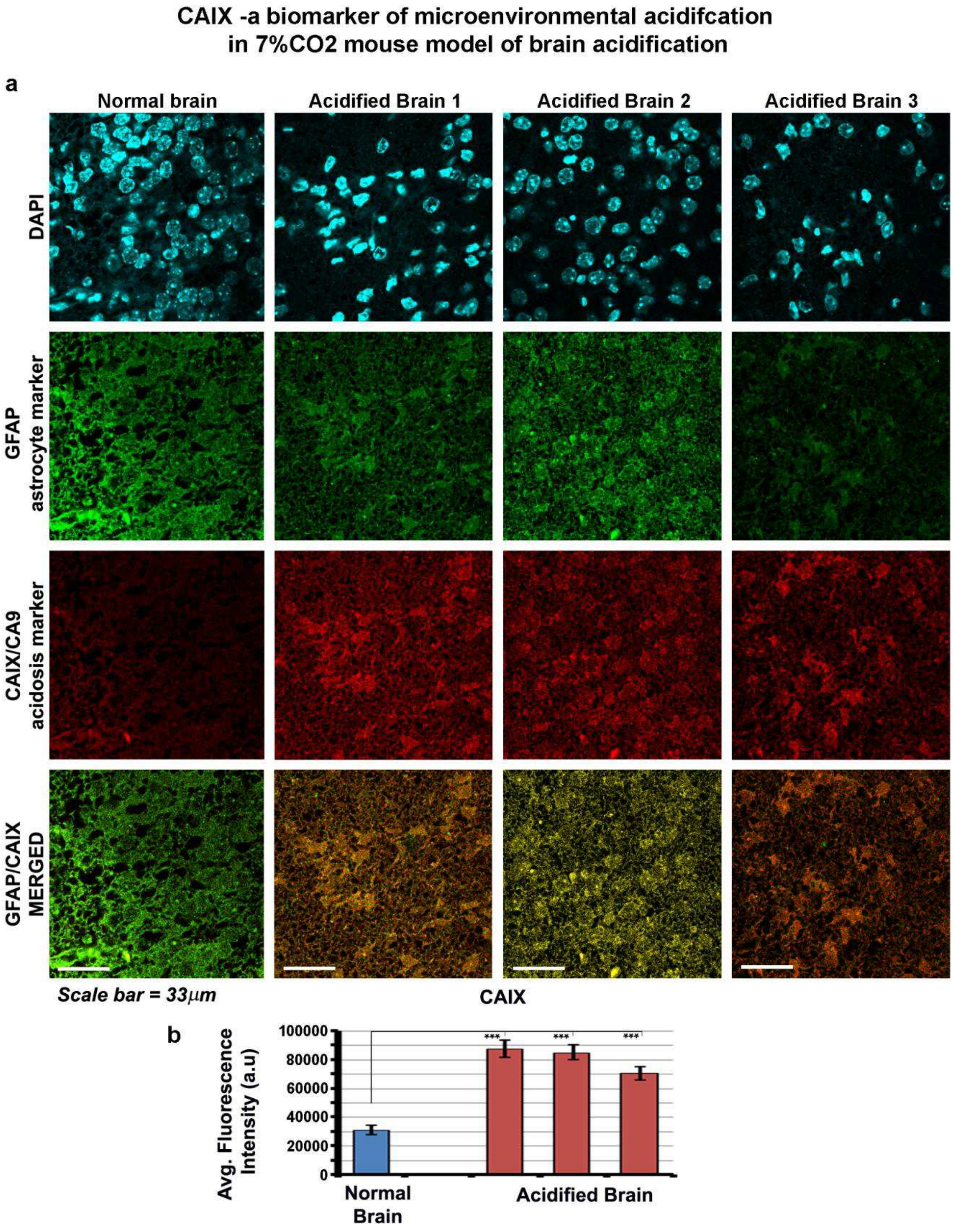

Fig. S18: In vivo mouse model of brain acidification faithfully upregulates CAIX/CA9 protein, a marker of the acidic microenvironment.

Mouse brain acidification was generated by 7% CO2 inhalation for 2.5 hours. See the methods section for more details.

(a,b) Colocalization of GFAP (an astrocyte marker) with CAIX (a surface protein expressed on cells in an acidic microenvironment) clearly shows that the procedure faithfully generates

an acidic microenvironment in the brain. CAIX expression was quantified by measuring fluorescence intensity in 5 random fields per section per 3 independent mouse brains, using Fiji software. The brain regions used for imaging and intensity quantification per section were kept the same across independent brains. All datasets are reported as mean± SD. Significance is shown as *p<0.05, **p<0.01, ***p<0.001. Mean is derived from 3 independent experiments.

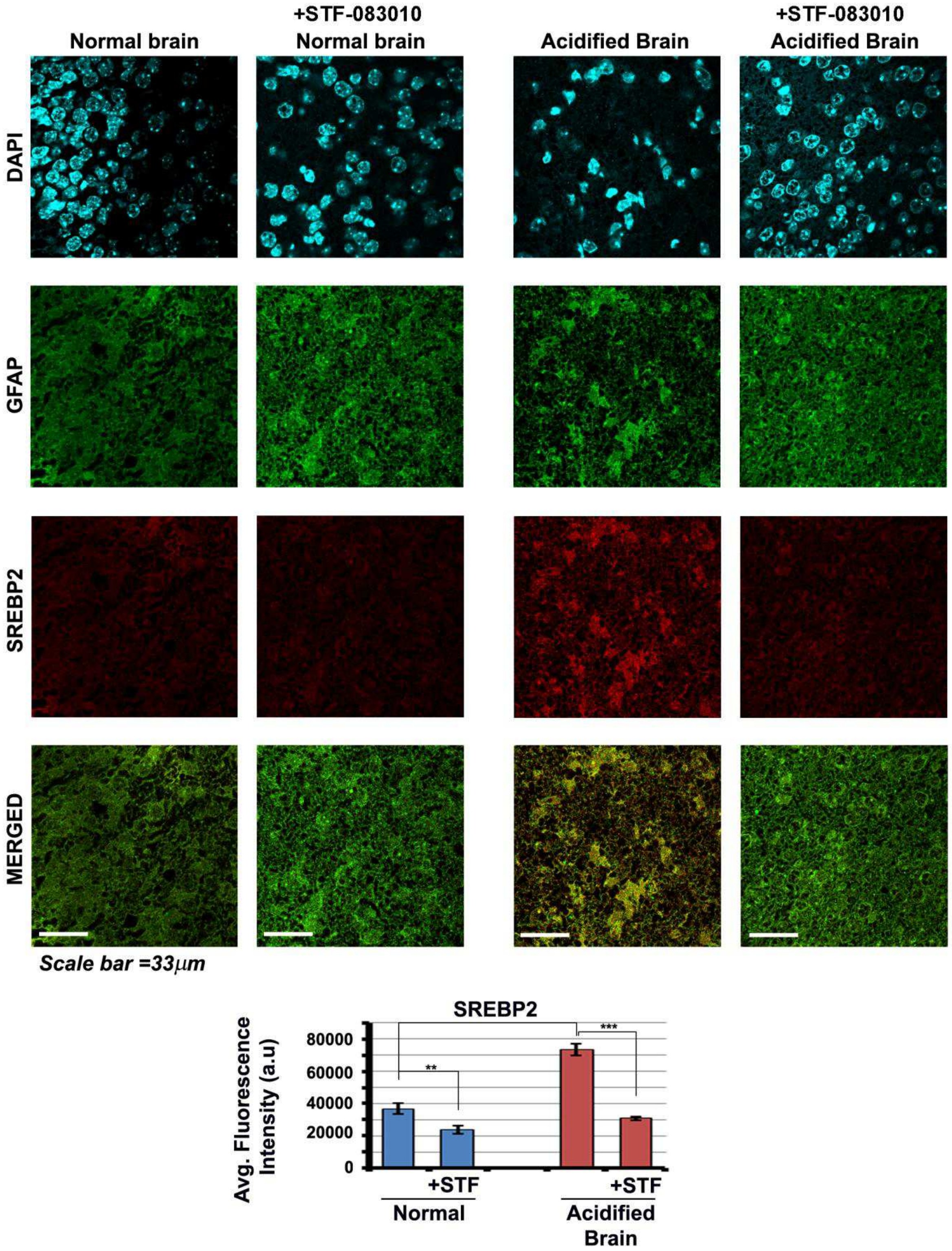

Fig. S19: In vivo mouse model of brain acidification faithfully upregulates SREBP2 in the acidified brain only in the presence of sXBP1.

Mouse brain acidification was generated by 7% CO2 inhalation for 2.5 hours. Mice were simultaneously either subcutaneously injected with STF-083010 or not. See the methods section for more details.

SREBP2 showed significant nuclear translocation in the astrocytes from acidified brain vs normal brain, which was not so in STF-083010 injected acidified brains. GFAP is an astrocyte marker.STF-083010 inhibits sXBP1 generation.

SREBP2 expression was quantified by measuring fluorescence intensity in 5 random fields per section per 3 independent mouse brains, using Fiji software. The brain regions used for imaging and intensity quantification per section were kept the same across independent brains. All datasets are reported as mean± SD. Significance is shown as *p<0.05, **p<0.01, ***p<0.001. Mean is derived from 3 independent experiments.

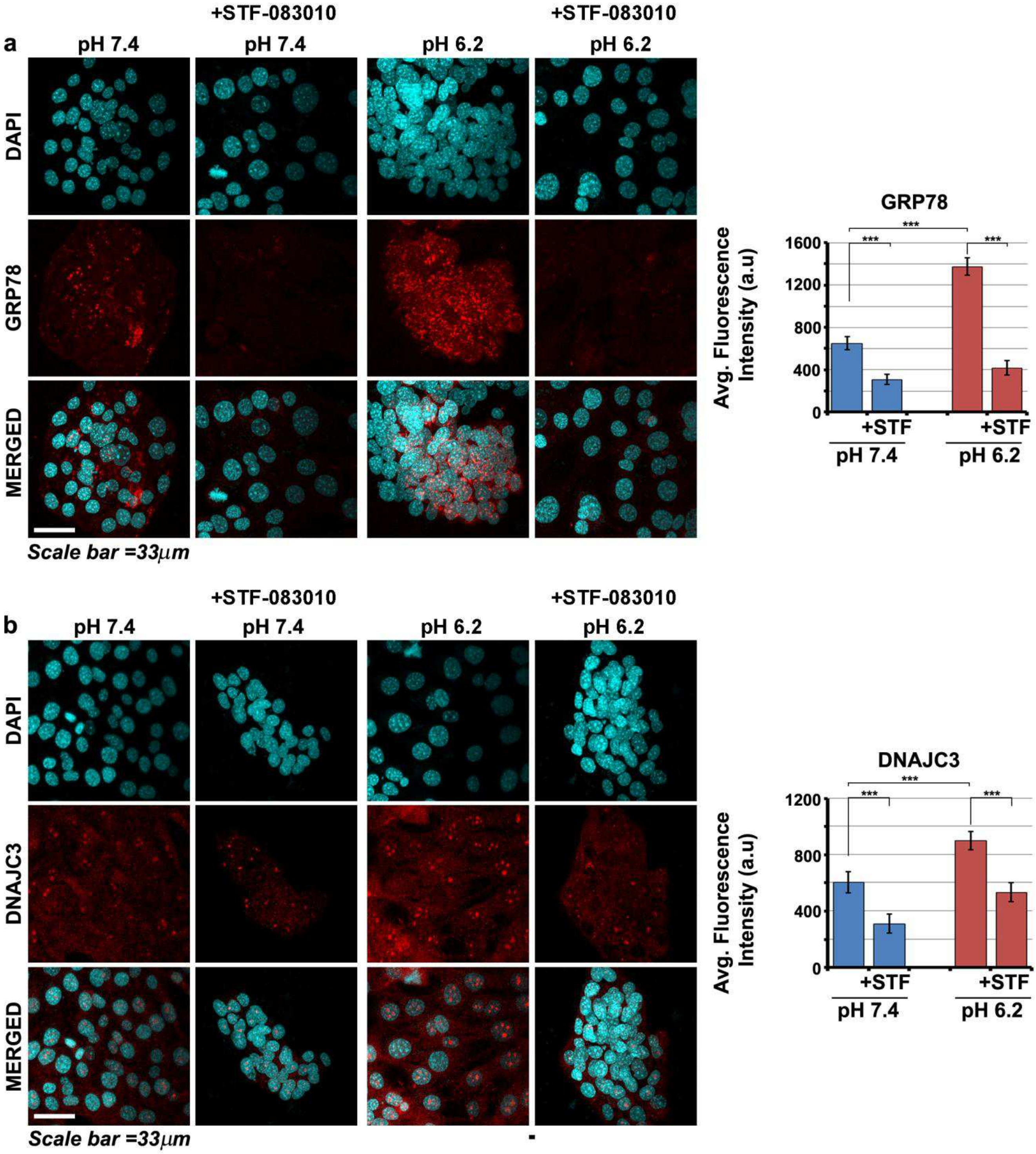

Fig. S20: GRP78 and DNAJC3, the downstream cytoprotective targets of sXBP1 are inhibited by STF-083010 treatment in low pH treated mouse primary astrocytes.

Mouse primary astrocytes were treated with sXBP1 inhibitor (STF-083010) or not for 2 hours. Astrocytes were further incubated with physiological and low pH media for 8hrs (as sXBP1 mRNA transcript is determined to be upregulated within 6-8 hrs. STF-083010 was also added in the medium during pH treatments. Post incubation, cells were fixed in 1.5%

PFA, washed in 1X PBS, permeabilized with 0.25% saponin and incubated with anti-GRP78 and anti-DNAJC3 antibodies for double immunolabelling at 4^oC for 16 hrs.

(a,b) GRP78 and DNAJC3 are upregulated by the cytoprotective arm of the ER stress. These are also downstream targets of sXBP1. Acidified astrocytes showed significant levels of these cytoprotective proteins, which was not so in STF-083010 treated astrocytes.

Imagings of GRP78 and DNAJC3 in each condition were performed in a confocal microscope. Image acquisition conditions in each channel were kept the same in each condition over independent experiments. At least 50-70 cells from random fields were taken for single-cell measurements, in each pH condition, from 3 independent experiments. The expression of each antigen was analyzed in Fiji software. All datasets are reported as mean± SD. Significance is shown as *p<0.05, **p<0.01, ***p<0.001. Mean is derived from 3 independent experiments

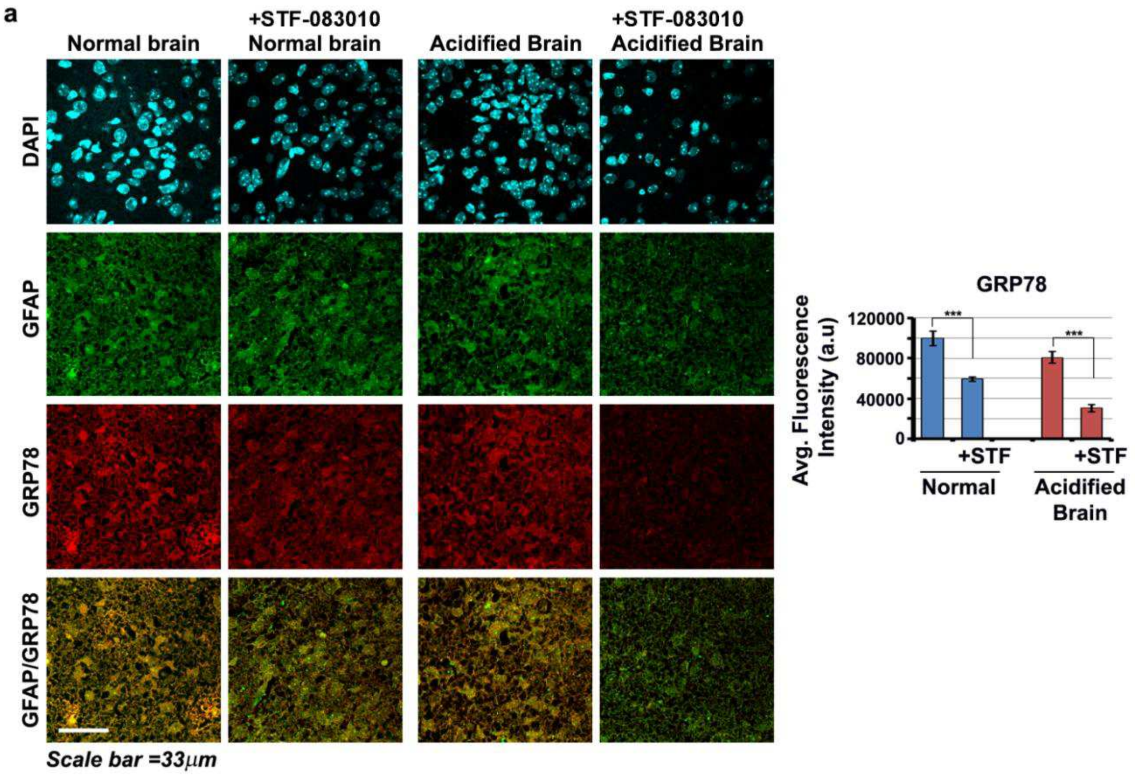

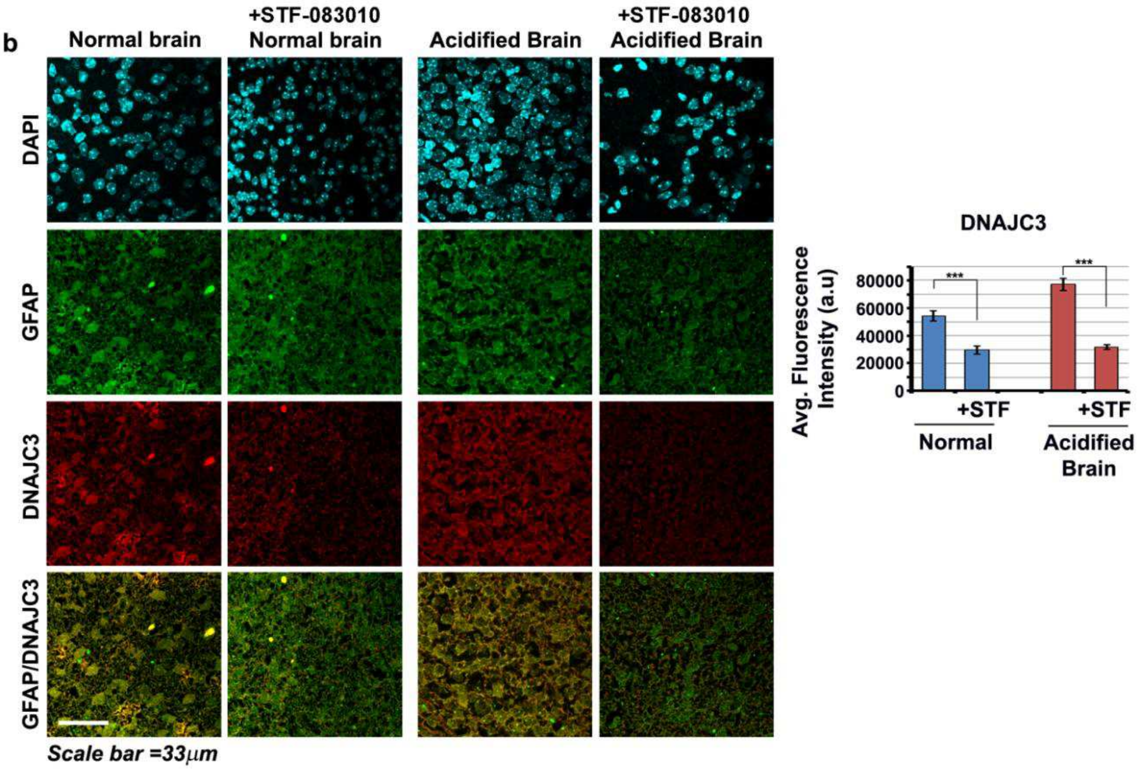

Fig. S21: GRP78 and DNAJC3, the downstream cytoprotective targets of sXBP1 are inhibited by STF-083010 treatment in acidified brain.

Mouse brain acidification was generated by 7% CO2 inhalation for 2.5 hours. Mice were simultaneously either subcutaneously injected with STF-083010 or not. See the methods section for more details.

(a,b) GRP78 and DNAJC3 are upregulated by the cytoprotective arm of the ER stress. These are also downstream targets of sXBP1. Acidified mouse brain showed significant levels of these cytoprotective proteins in the astrocytes from acidified brain vs normal brain, which was not so in STF-083010 injected acidified brains. GFAP is an astrocyte marker. GRP78 and DNAJC3 expression were quantified by measuring fluorescence intensity in 5 random fields per section per 3 independent mouse brains, using Fiji software. The brain regions used for imaging and intensity quantification per section were kept the same across independent brains across antigen. All datasets are reported as mean± SD. Significance is shown as *p<0.05, **p<0.01, ***p<0.001. Mean is derived from 3 independent experiments.

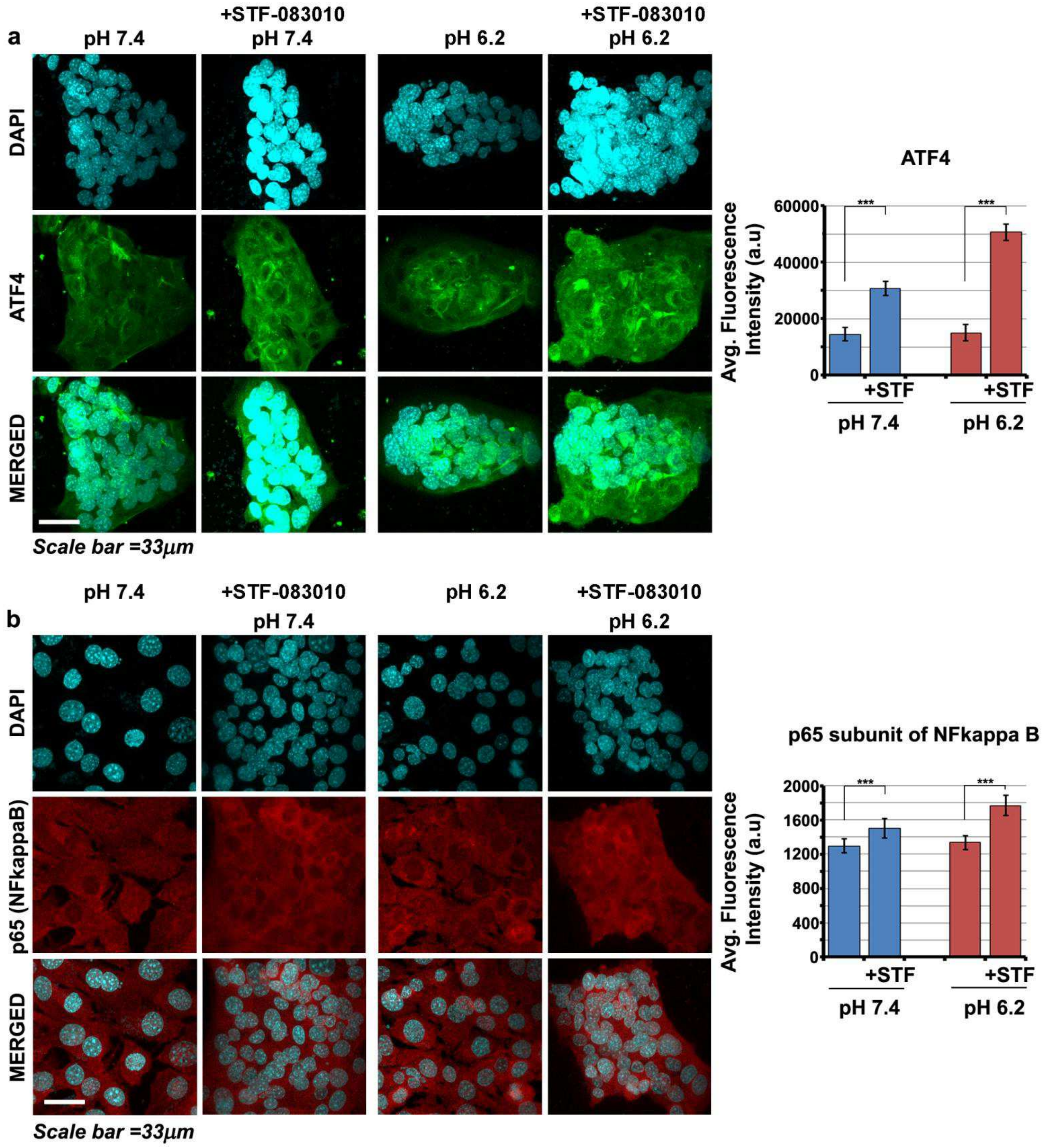

Fig. S22: ATF4 and NF-kB, the downstream cell death associated targets of ER stress are upregulated by STF-083010 treatment in low pH treated mouse primary astrocytes.

(a,b) Mouse primary astrocytes were treated with sXBP1 inhibitor (STF-083010) or not for 2 hours. Astrocytes were further incubated with physiological and low pH media for 8hrs (as sXBP1 mRNA transcript is determined to be upregulated within 6-8 hrs). STF-083010 was also added in the medium during pH treatments. Post incubation, cells were fixed in 1.5%

PFA, washed in 1X PBS, permeabilized with 0.25% saponin and incubated with anti-GRP78 and anti-DNAJC3 antibodies for double immunolabelling at $4^{o}C$ for 16 hrs. ATF4 and NF-kB, the downstream cell death associated targets of ER stress, were kept low in acidified astrocytes which were not so when acidified astrocytes were also treated with STF-083010.

Imagings of ATF4 and NF-kB in each condition were performed in a confocal microscope. Image acquisition conditions in each channel were kept the same in each condition over independent experiments. At least 50-70 cells from random fields were taken for single-cell measurements, in each pH condition, from 3 independent experiments. The expression of each antigen was analyzed in Fiji software. All datasets are reported as mean± SD. Significance is shown as *p<0.05, **p<0.01, ***p<0.001. Mean is derived from 3 independent experiments.

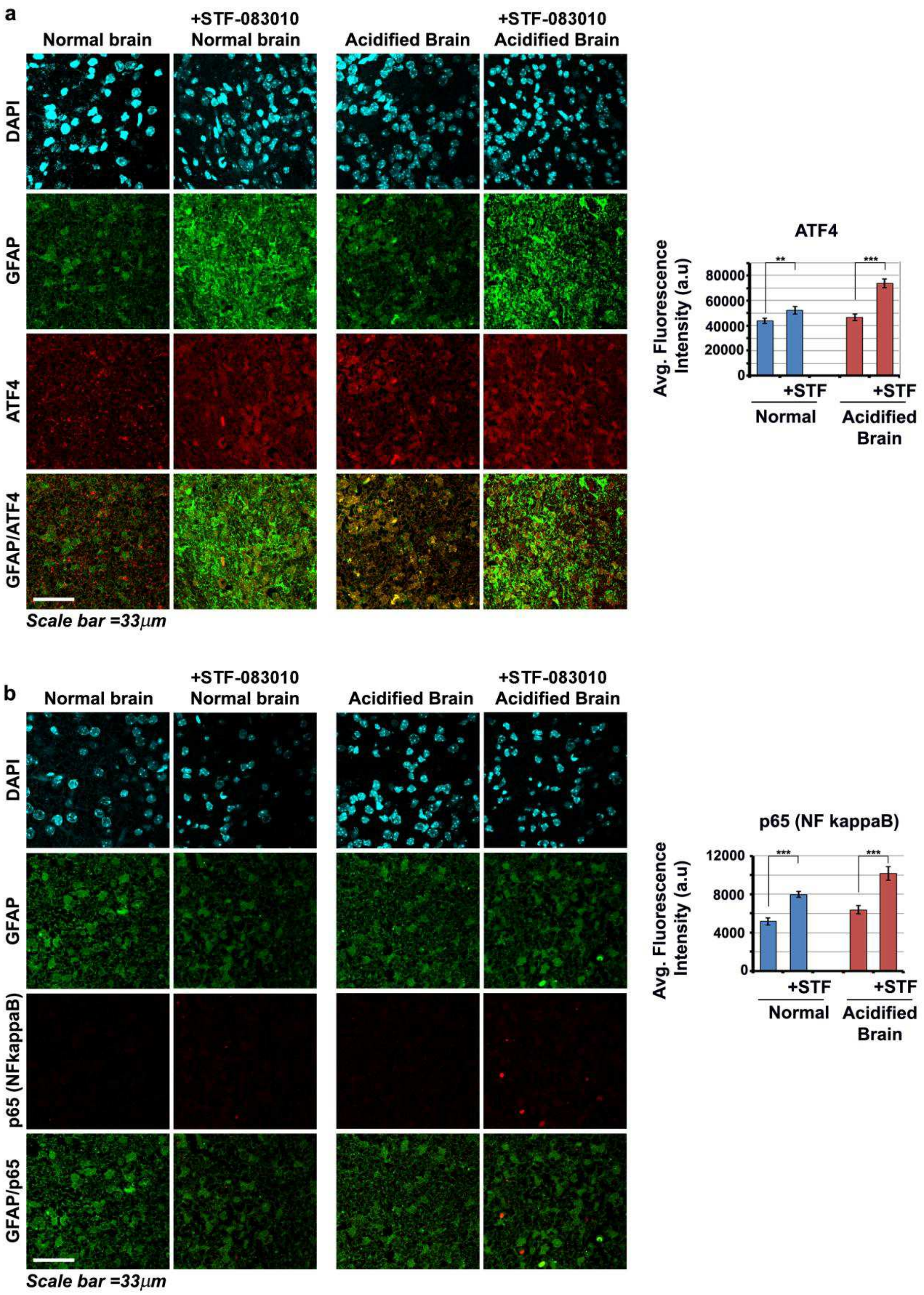

Fig. S23: ATF4 and NF-kB, the downstream cell death associated targets of ER stress are upregulated by STF-083010 treatment in acidified brain.

Mouse brain acidification was generated by 7% CO2 inhalation for 2.5 hours. Mice were simultaneously either subcutaneously injected with STF-083010 or not. See the methods section for more details.

(a,b) ATF4 and p65 (subunit of NF-kB) are upregulated by the cell death associated arm of the ER stress. Acidified mouse brain showed significantly lower levels of these proteins in the astrocytes from acidified brain vs normal brain, which was not so in STF-083010 injected acidified brains. GFAP is an astrocyte marker. ATF4 and p65 (subunit of NF-kB) expression were quantified by measuring fluorescence intensity in 5 random fields per section per 3 independent mouse brains, using Fiji software. The brain regions used for imaging and intensity quantification per section were kept the same across independent brains across antigen. All datasets are reported as mean± SD. Significance is shown as *$p<0.05$, **$p<0.01$, ***$p<0.001$. Mean is derived from 3 independent experiments.

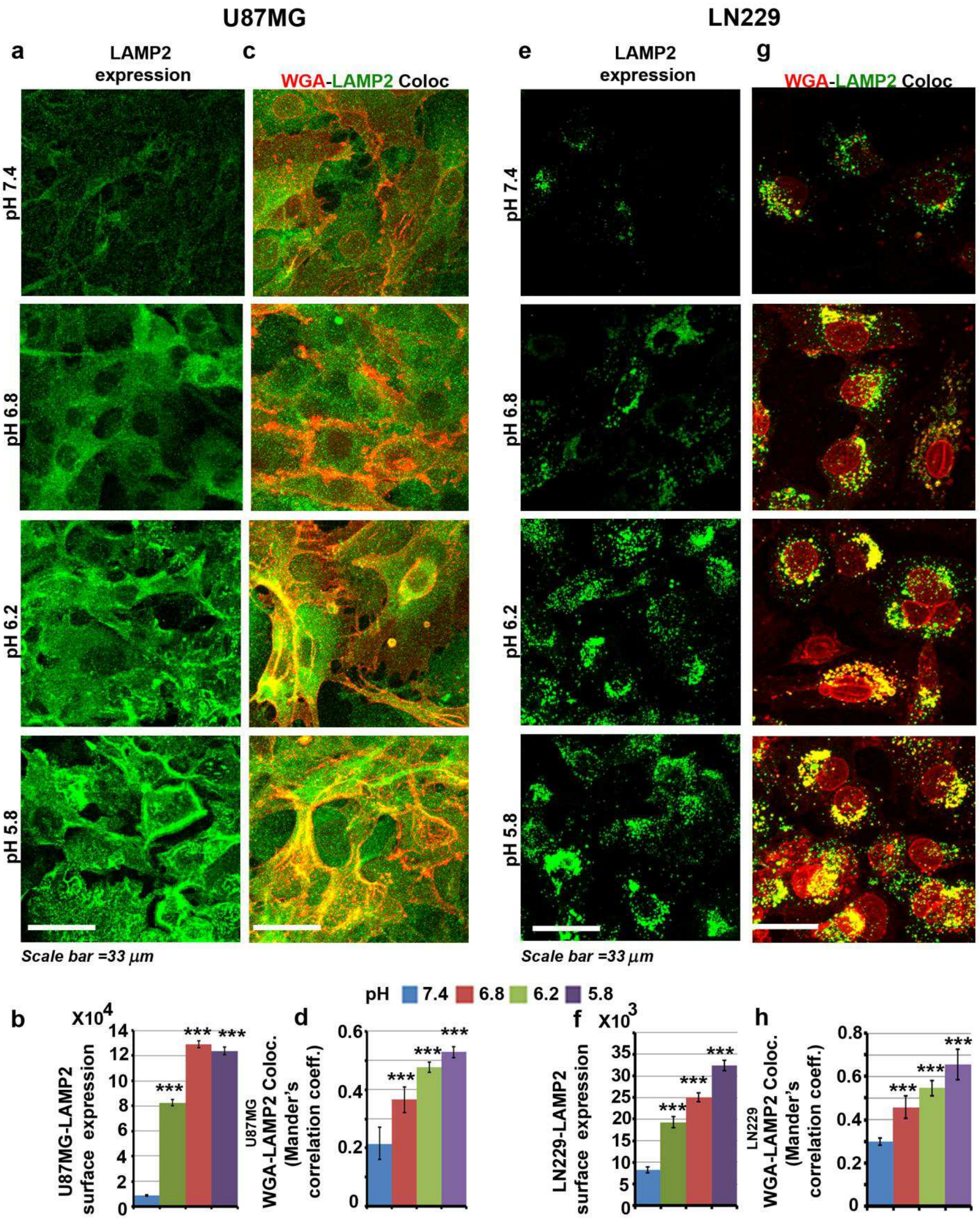

Fig. S24: Surface expression and colocalization of LAMP2 with plasma membrane marker WGA in glioblastoma tumour cell lines exposed to different pH units.

GBM tumour cell lines (U87MG, LN229) were incubated in different pH conditions for 4 hrs. Post incubation, cells were fixed in 1.5% PFA, washed in 1X PBS and incubated with the anti-LAMP2 primary antibody on the cell surface (without permeabilisation step) for 16 hrs at 4°C.

The surface LAMP2 expression signal was developed with AlexaFluor 488 conjugated secondary antibody **(a,e)**. Post this, WGA conjugated with AlexaFluor 594, a cell surface marker, was incubated on cells for 45 minutes **(c,g)**. The cells were washed and mounted in 70% glycerol/PBS mountant.

Sequential imaging of surface LAMP2 and WGA signals were performed in each condition via confocal microscopy. Image acquisition conditions in each channel were kept the same in each condition over independent experiments. At least 60 cells from random fields were taken for single-cell measurements, in each pH condition, from 3 independent experiments. **(a,e** and **b,f)** Fiji software was used to analyse the expression **(c,g** and **d,h)** colocalization of LAMP2 with WGA.

All datasets are reported as mean± SD. Significance is shown as *p<0.05, **p<0.01, ***p<0.001. Mean is derived from 3 independent experiments.

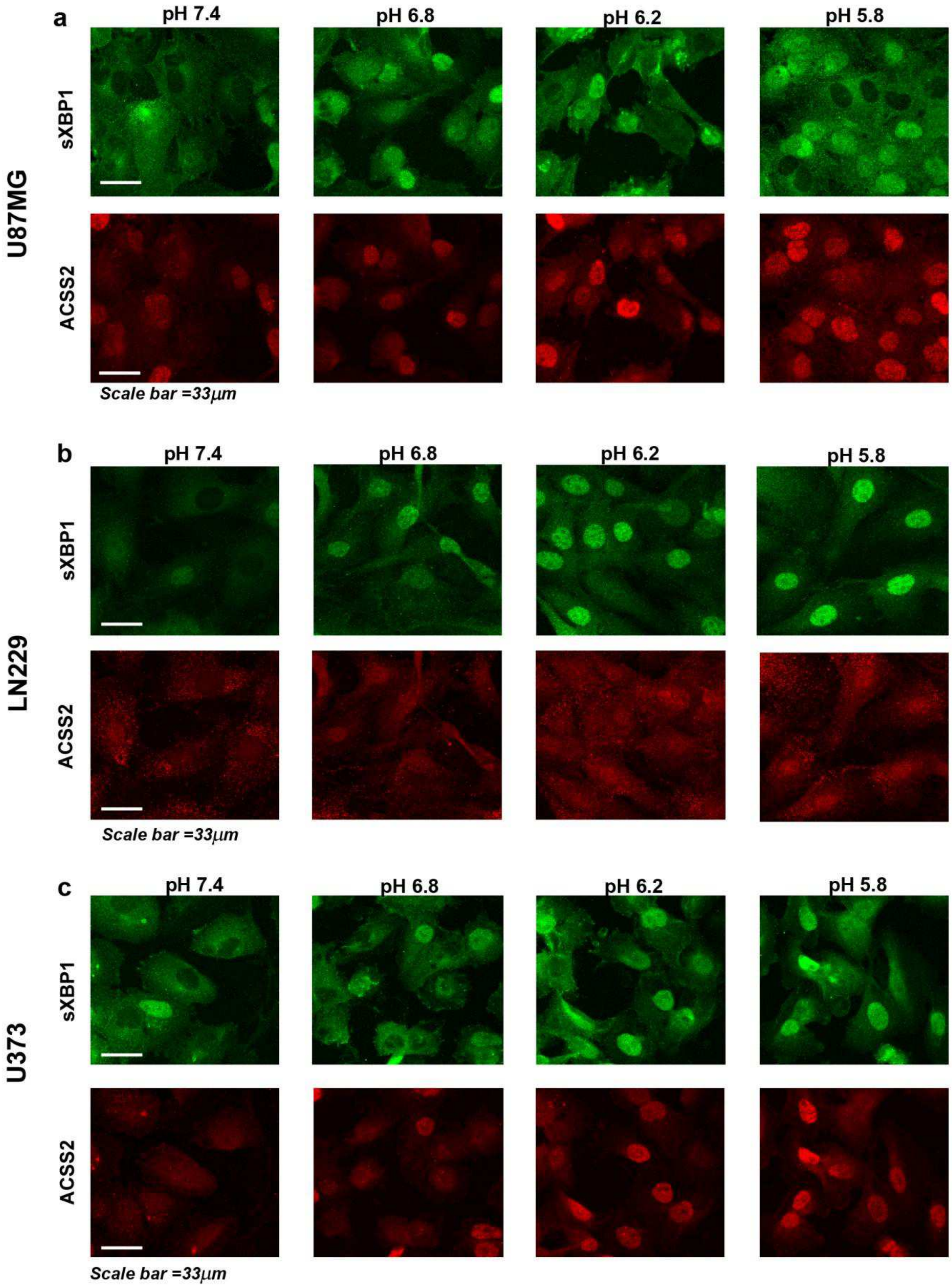

Fig. S25: Representative images of the expression of sXBP1 and ACSS2 in GBM tumour cell lines exposed to different pH units, related to Fig. 8a,b

(a-c) GBM tumour cell lines (U87MG, LN229 and U373) were incubated in different pH conditions for 8 hrs (as sXBP1 mRNA transcript is determined to be upregulated within 6-8

hrs). Post incubation, cells were fixed in 1.5% PFA, washed in 1X PBS, permeabilised with 0.25% saponin and incubated with anti-sXBP1 and anti-ACSS2 antibody for double immunolabelling at 4°C for 16 hrs. The sXBP1 signal was developed with AlexaFluor 488 conjugated secondary antibody, and that of ACSS2 was developed by AlexaFluor 594 conjugated antibody.

Sequential imaging of sXBP1 and ACSS2 in each condition were performed in a confocal microscope. Image acquisition conditions in each channel were kept the same in each condition over independent experiments. At least 50-70 cells from random fields were taken for single-cell measurements, in each pH condition, from 3 independent experiments. Cell total and nuclear expression were analysed in Fiji software.

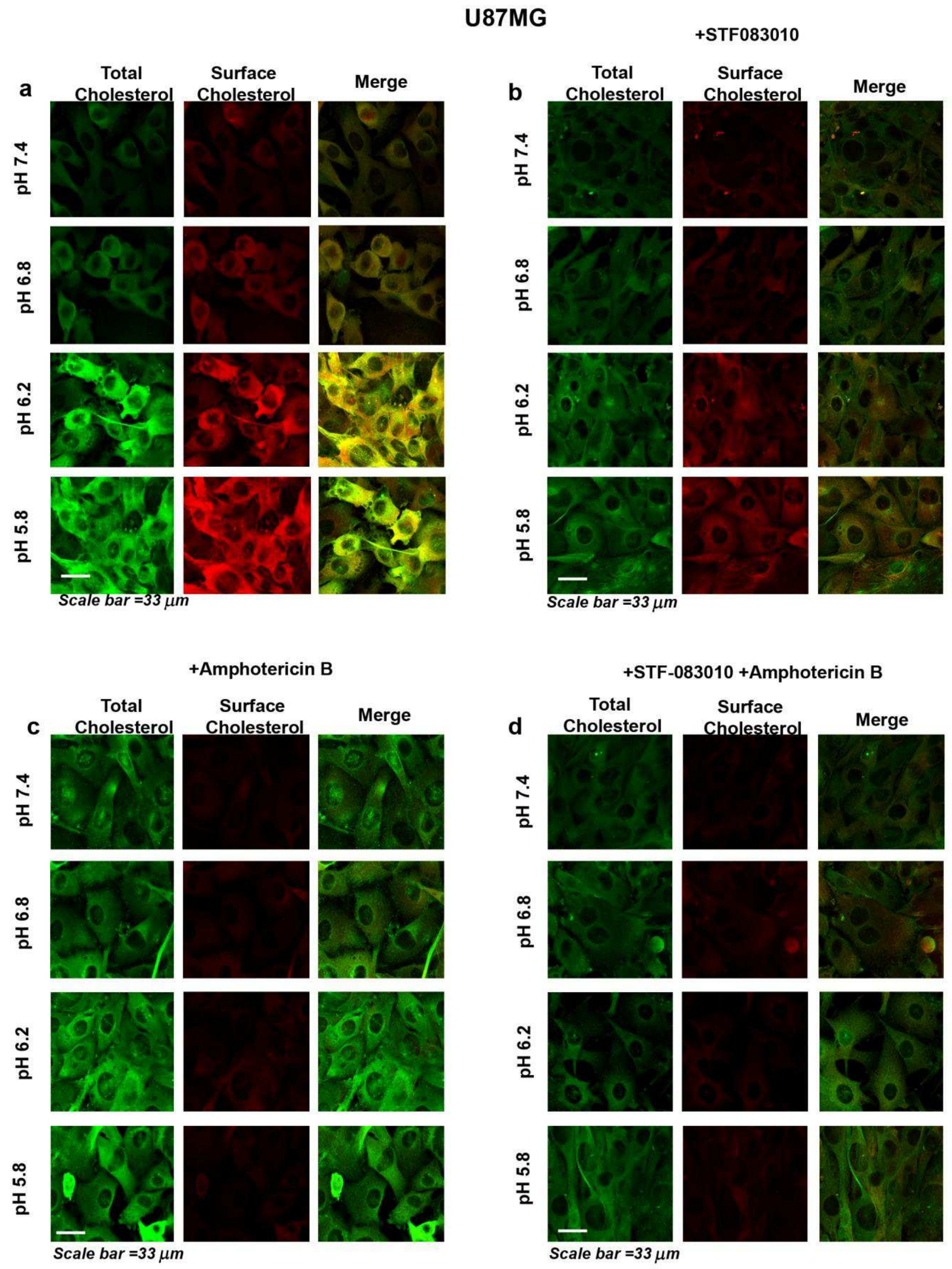

Fig. S26: Representative images of the surface and total cholesterol levels in U87MG glioblastoma tumour cell line exposed to different pH units, related to Fig. 9a.

(a-d) U87MG GBM cells were incubated with different pH media for 4 hrs followed by another 12 hrs of drug treatments or not in the respective pHs. The drug treatments were as follows (i) STF-083010 (60µM), (ii) Amphotericin B (10µM) (iii) STF-083010 +

Amphotericin B at respective pHs. Cells were then fixed with 1.5% PFA, washed in 1X PBS, and the anti-cholesterol primary antibody was incubated on the cell surface (without permeabilization step) for 16 hrs at 4^oC. Post this; cells were incubated in AlexaFluor 594 conjugated secondary antibody. Cells were fixed again in 1.5% PFA, washed and permeabilized with 0.25% saponin for 20 minutes. After washing steps, the anti-cholesterol primary antibody was again incubated, and a signal for total cholesterol was developed with AlexaFluor 488 conjugated secondary antibody.

Imaging of surface and total cholesterol signals in each condition were performed in a confocal microscope. Image acquisition conditions in each channel were kept the same in each condition over independent experiments. At least 50-70 cells from random fields were taken in single-cell measurements, in each pH condition, from 3 independent experiments. Expression was analyzed in Fiji software.

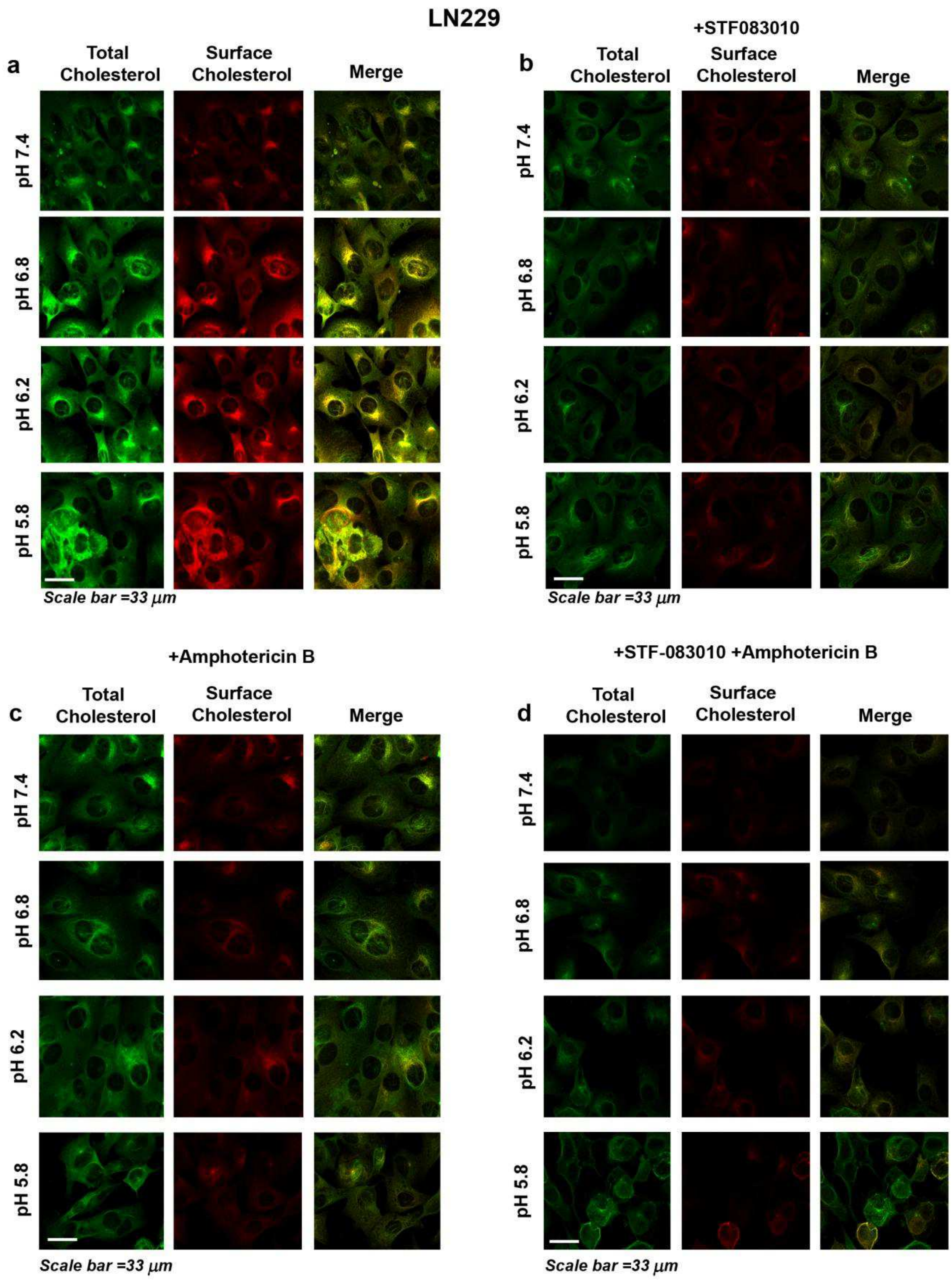

Fig. S27: Representative images of the surface and total cholesterol levels in LN229 glioblastoma tumour cell line exposed to different pH units with or without drug treatments, related to Fig. 9b.

(a-d) LN229 GBM tumour cells were incubated with different pH media for 4 hrs followed by another 12 hrs of drug treatments or not in the respective pHs. The drug treatments were as follows (i) STF-083010 (60µM), (ii) Amphotericin B (10µM) (iii) STF-083010 +

Amphotericin B at respective pHs. Cells were then fixed with 1.5% PFA, washed in 1X PBS, and the anti-cholesterol primary antibody was incubated on the cell surface (without permeabilization step) for 16 hrs at 4°C. Post this; cells were incubated in AlexaFluor 594 conjugated secondary antibody. Cells were fixed again in 1.5% PFA for 20 min at RT, were then washed and permeabilized with 0.25% saponin for 20 min. After washing steps, the anti-cholesterol primary antibody was again incubated, and a signal for total cholesterol was developed with AlexaFluor 488 conjugated secondary antibody.

Imaging of surface and total cholesterol signals in each condition were performed in a confocal microscope. Image acquisition conditions in each channel were kept the same in each condition over independent experiments. At least 50-70 cells from random fields were taken in single-cell measurements, in each pH condition, from 3 independent experiments. Expression was analyzed in Fiji software.

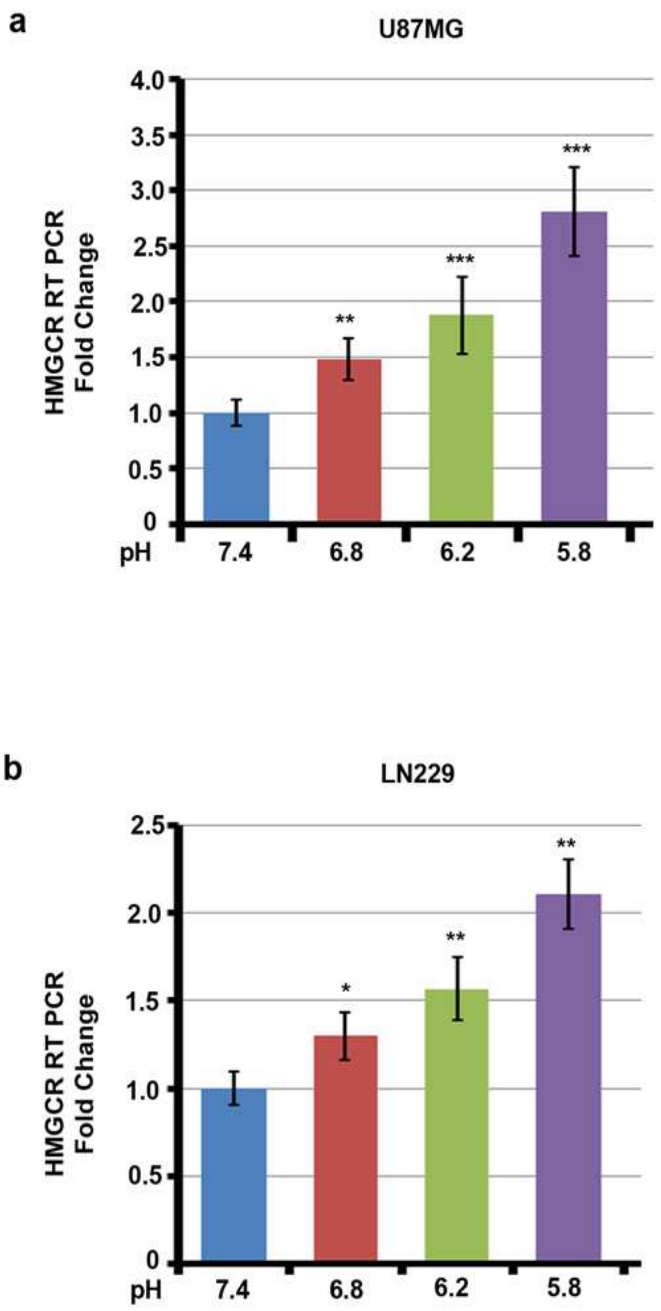

Fig. S28: mRNA expression of HMGCR in GBM cells exposed to different pH units, **related to Fig. 9c**.

(a,b) Cells (U87MG and LN229) were incubated with different pH media for 16 hours, matching the time point analysis of the LDL uptake assay. After treatments, RNA was isolated, and HMGCR, a rate-limiting enzyme in cholesterol biosynthesis, was analyzed. Results showed a significant rise in HMGCR expression within the short time when GBM cells were exposed to low pH media. All datasets are reported as mean± SD. Significance is shown as *p<0.05, **p<0.01, ***p<0.001. Mean is derived from 3 independent experiments.

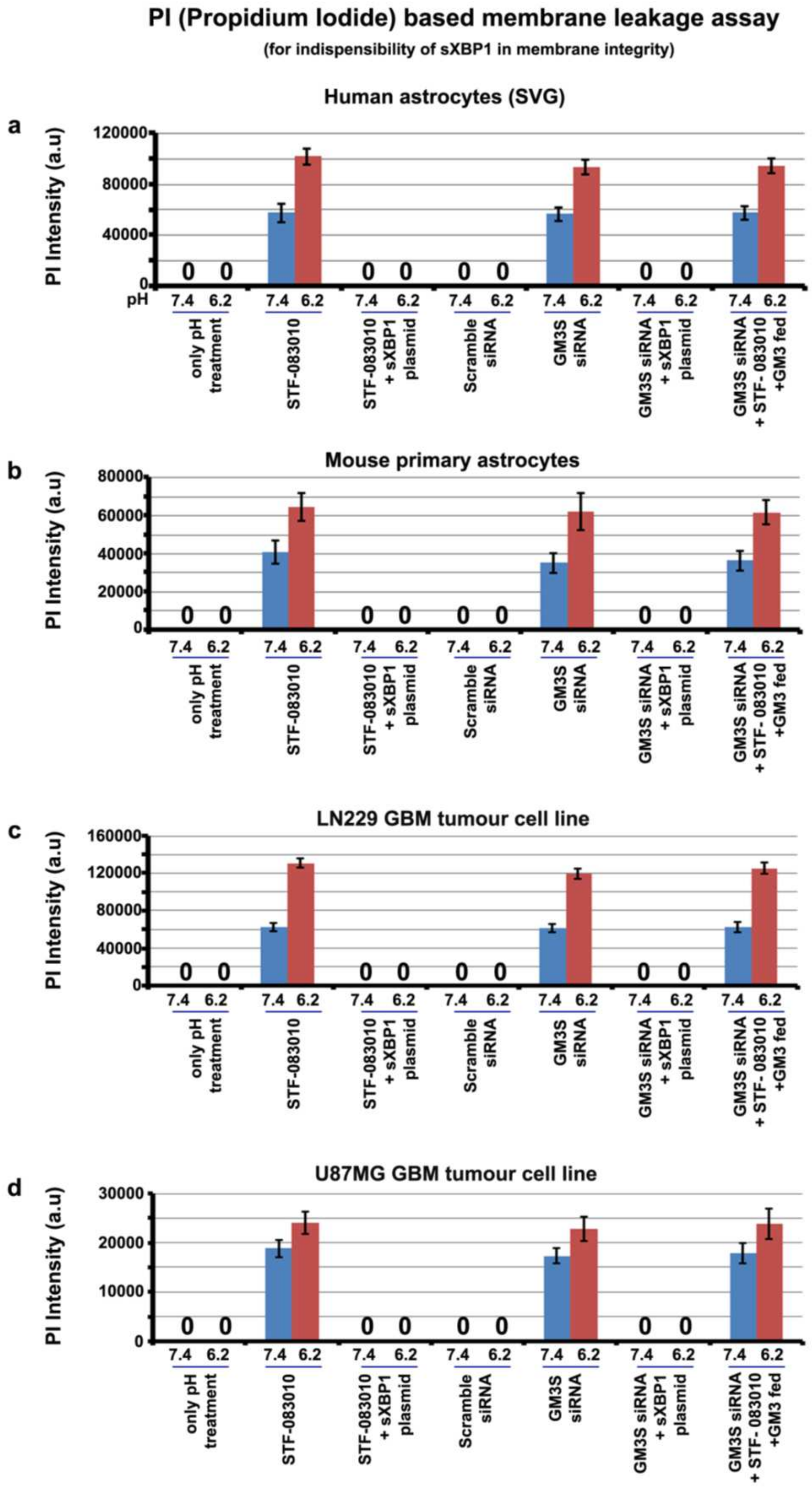

Fig. S29: Propidium Iodide (PI) based membrane leakage assay shows that sXBP1 is a crucial downstream effector of GM3 in the generation of low pH adaptation in astrocytes and astrocytic tumours:

(a-b) Astrocytes (human and mouse) as well as **(c-d)** astrocytic tumours cells (LN229 and U87MG) were treated with sXBP1 inhibitor, STF-083010 for 2 hours before pH treatments. STF-083010 was further added in media adjusted to normal or low pH. Cells were treated with the pH±STF for 8 hours. PI was added to the medium in the last 3 minutes of the incubation time. The fluorescence intensity of PI was quickly captured in each condition and quantified using Fiji software. Cells treated with STF-083010 showed leakage in both normal and low pH. However, no significant membrane leakage was observed when cells were priorly transfected with sXBP1 and then the pH±STF-083010 regime was followed. Besides, in cells depleted of GM3 via downregulation of its synthesis enzymes GM3S, if sXBP1 was expressed via plasmid co-transfection, no membrane leakage ensued. Crucially, if GM3 depleted cells were also co-treated with STF, then exogenous supplementation of GM3 lipid could not rescue the leakage in the absence of sXBP1. This shows that sXBP1 is a non-replaceable downstream target of GM3 in disabling membrane leakage, more prominently in low pH microenvironments. All datasets are reported as mean± SD. Significance is shown as *$p<0.05$, **$p<0.01$, ***$p<0.001$. Mean is derived from 3 independent experiments.

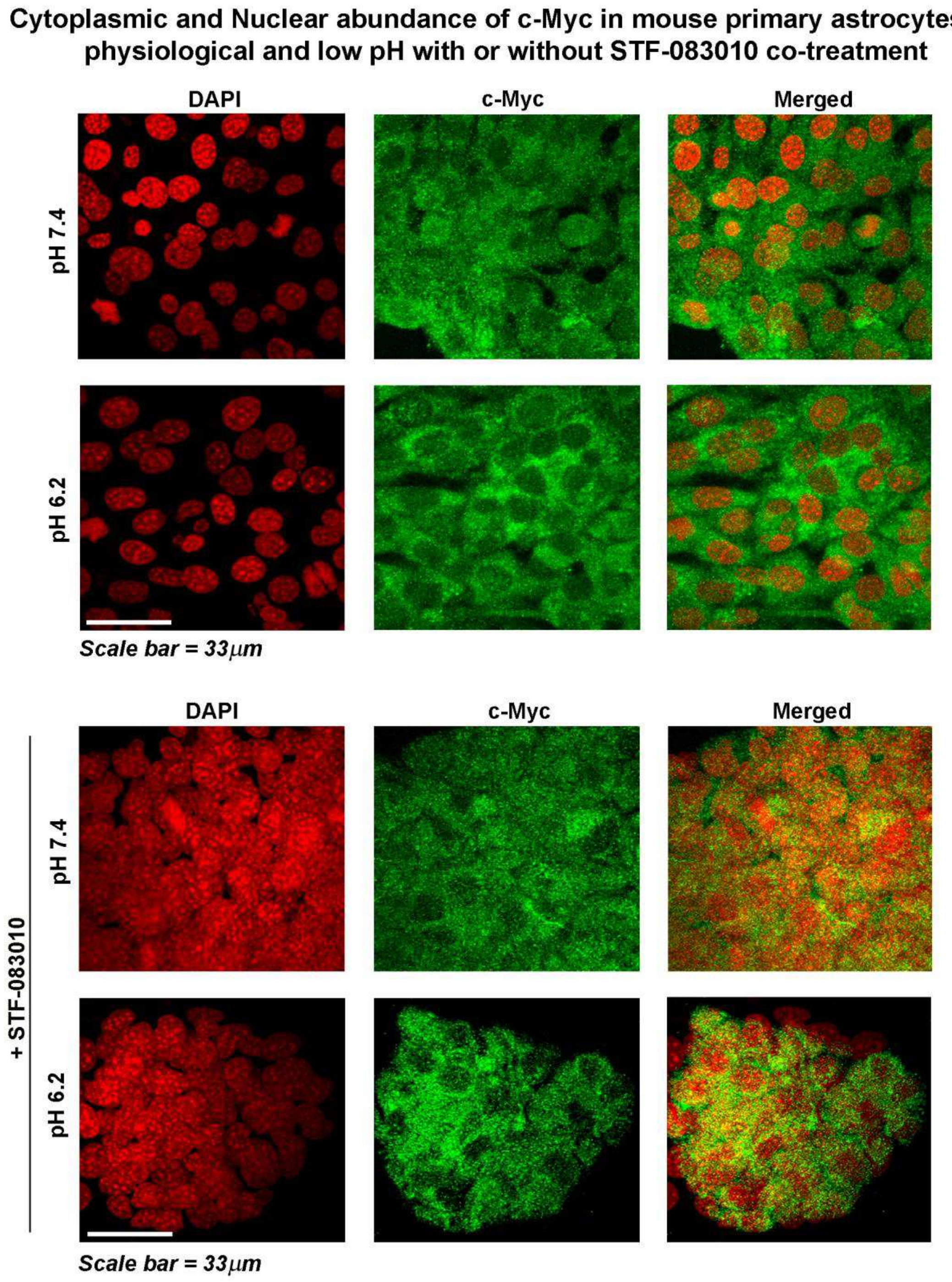

Fig. S30: c-Myc, a crucial oncogene, is <u>not</u> a non-redundant downstream target of sXBP1 and cannot by itself rescue astrocyte membrane leakage in a low pH microenvironment.

Mouse primary astrocytes were treated with sXBP1 inhibitor (STF-083010) or not for 2 hours. Astrocytes were further incubated with physiological and low pH media for 8hrs (as sXBP1 mRNA transcript is determined to be upregulated within 6-8 hrs). STF-083010 was also added in the medium during pH treatments. Post incubation, cells were fixed in 1.5% PFA, washed in 1X PBS, permeabilized with 0.25% saponin and incubated with the anti-

cMyc antibody for immunolabelling at 4°C for 16 hrs. The developed signal shows that both normal and low pH treated mouse primary astrocytes express high levels of c-Myc even in the absence of sXBP1. **Fig. S29** shows that the absence of sXBP1 causes membrane leakage, leading to cell death. Therefore, in the absence of sXBP1, c-Myc is abundantly expressed but by itself was not sufficient in enabling cell protection.

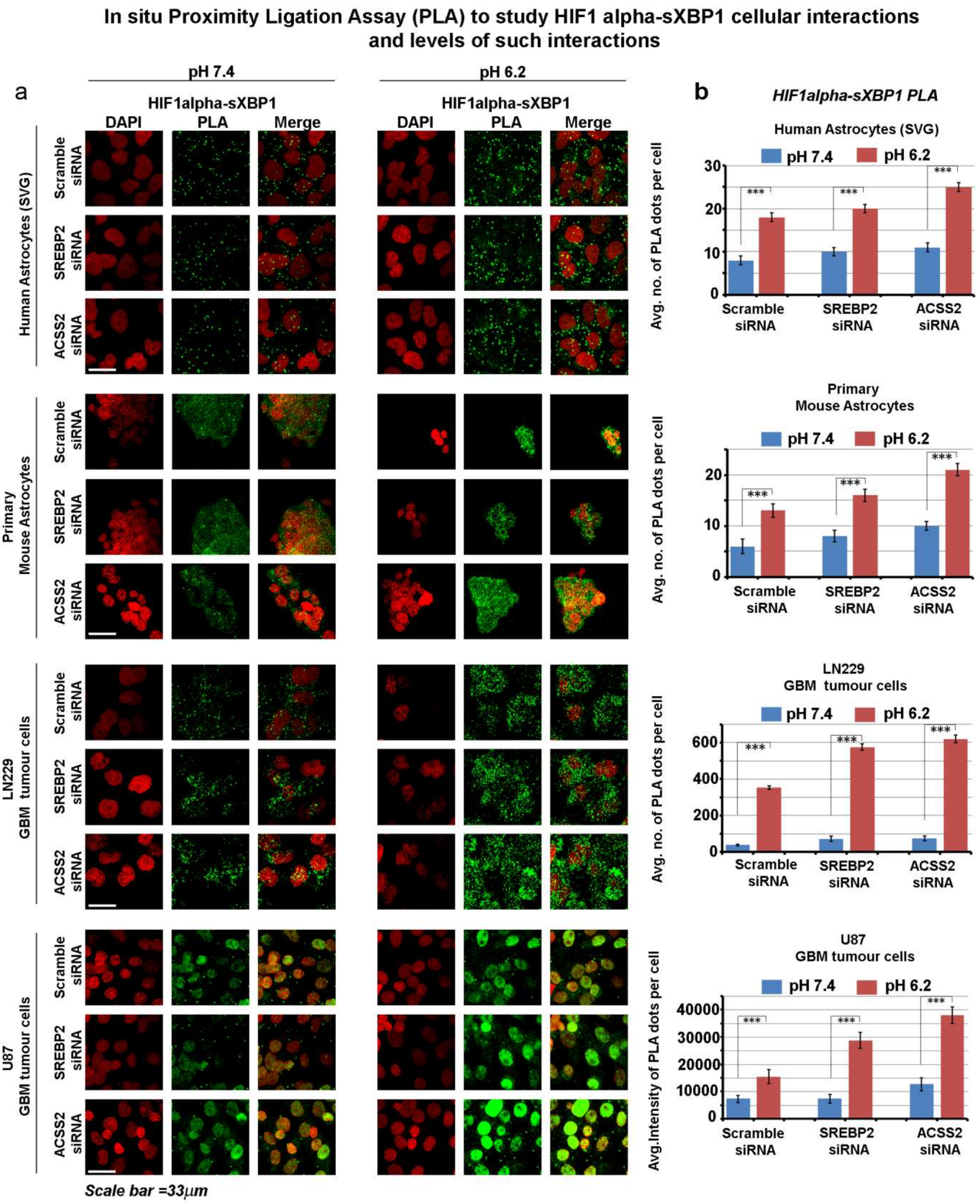

Fig. S31: In situ proximity ligation assay (PLA) shows that HIF1α interaction with sXBP1is not reduced in astrocytes and astrocytic tumours cells that were depleted of SREBP2 and ACSS2.

Astrocytes (human and mouse primary) and astrocytic tumour cells (LN229 and U87MG) were treated with SREBP2/ACSS2 siRNA or not and were then subjected to normal and low pH treatments for 8 hours. HIF1α interaction with sXBP1 is reported to be associated with the survivability of tumour cells. In situ PLA assay (DUOLINK In situ, Sigma, USA) was

performed according to the manufacturer's instructions to identify the extent of HIF1α interaction with sXBP1 in cells depleted of sXBP1 lipogenesis targets SREBP2/ACSS2. **a)** The fluorescent dots that represent the positive protein-protein interaction events were counted per cell. The average number of fluorescent dots was arrived at by measuring 30 cells from each condition per independent experiment. Data was compiled from 3 independent experiments. In U87MG, the nuclear interaction of HIF1α-sXBP1 was so high and dense that counting individual dots was impossible. **b)** Data was therefore plotted as the average fluorescent intensity of PLA signal per cell. Results show that HIF1α interaction with sXBP1was not reduced in SREBP2/ACSS2 depleted condition. However, please follow **Fig S34**, which shows that cell leakage was induced in the absence of SREBP2/ACSS2. This means that HIF1α-sXBP1 interaction was insufficient to lend survivability to cells and that SREBP2/ACSS2 are the essential pro-survival targets of sXBP1 in the acidic microenvironment.

All datasets are reported as mean± SD. Significance is shown as *p<0.05, **p<0.01, ***p<0.001. Mean is derived from 3 independent experiments.

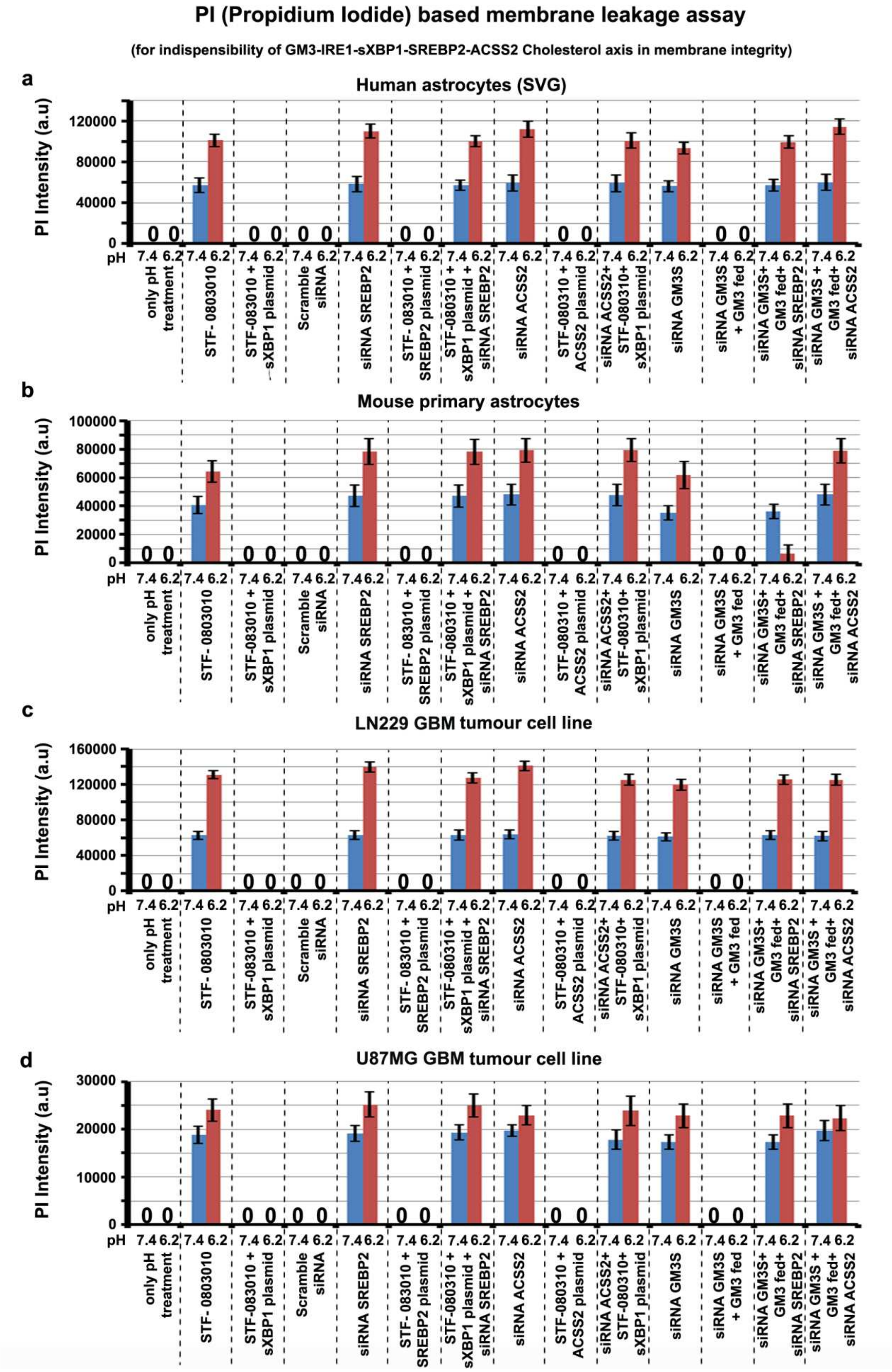

Fig. S32: Propidium Iodide (PI) based membrane leakage assay shows that SREBP2 and ACSS2 are the essential downstream prosurvival targets of GM3-sXBP1 low pH adaptation machinery in astrocytes and astrocytic tumours:

(a-b) Astrocytes (human and mouse) as well as (c-d) astrocytic tumours cells (LN229 and U87MG) were treated with sXBP1 inhibitor, STF-083010 for 2 hours before pH treatments. STF-083010 was further added in media adjusted to normal or low pH. Cells were treated with the pH±STF for 8 hours. PI was added to the medium in the last 3 minutes of the incubation time, and then the medium was quickly replaced with the non-PI added medium. The fluorescence intensity of PI was quickly captured in each condition and quantified using Fiji software.

Cells treated with STF-083010 showed leakage in both normal and low pH. No significant membrane leakage was observed when the pH±STF regime was followed on the cells transfected with sXBP1. Besides, cells depleted of SREBP2, ACSS2 and GM3 were susceptible to membrane leakage, indicating them as important prosurvival factors. No membrane leakage ensued when sXBP1 or GM3 depleted cells were supplemented with SREBP2 or ACSS2 via plasmid transfection. However, in sXBP1 depleted cells (via STF), mere supplementation of sXBP1 via plasmid transfection was insufficient if SREBP2 and ACSS2 were kept depleted. In the same way, in GM3 depleted cells, mere supplementation of exogenous GM3 lipid was not sufficient if SREBP2 and ACSS2 were kept depleted.

This shows that SREBP2 and ACSS2 were non-replaceable downstream targets of sXBP1/GM3 in disabling membrane leakage, more prominently in low pH microenvironments. All datasets are reported as mean± SD. Significance is shown as *$p<0.05$, **$p<0.01$, ***$p<0.001$. Mean is derived from 3 independent experiments.

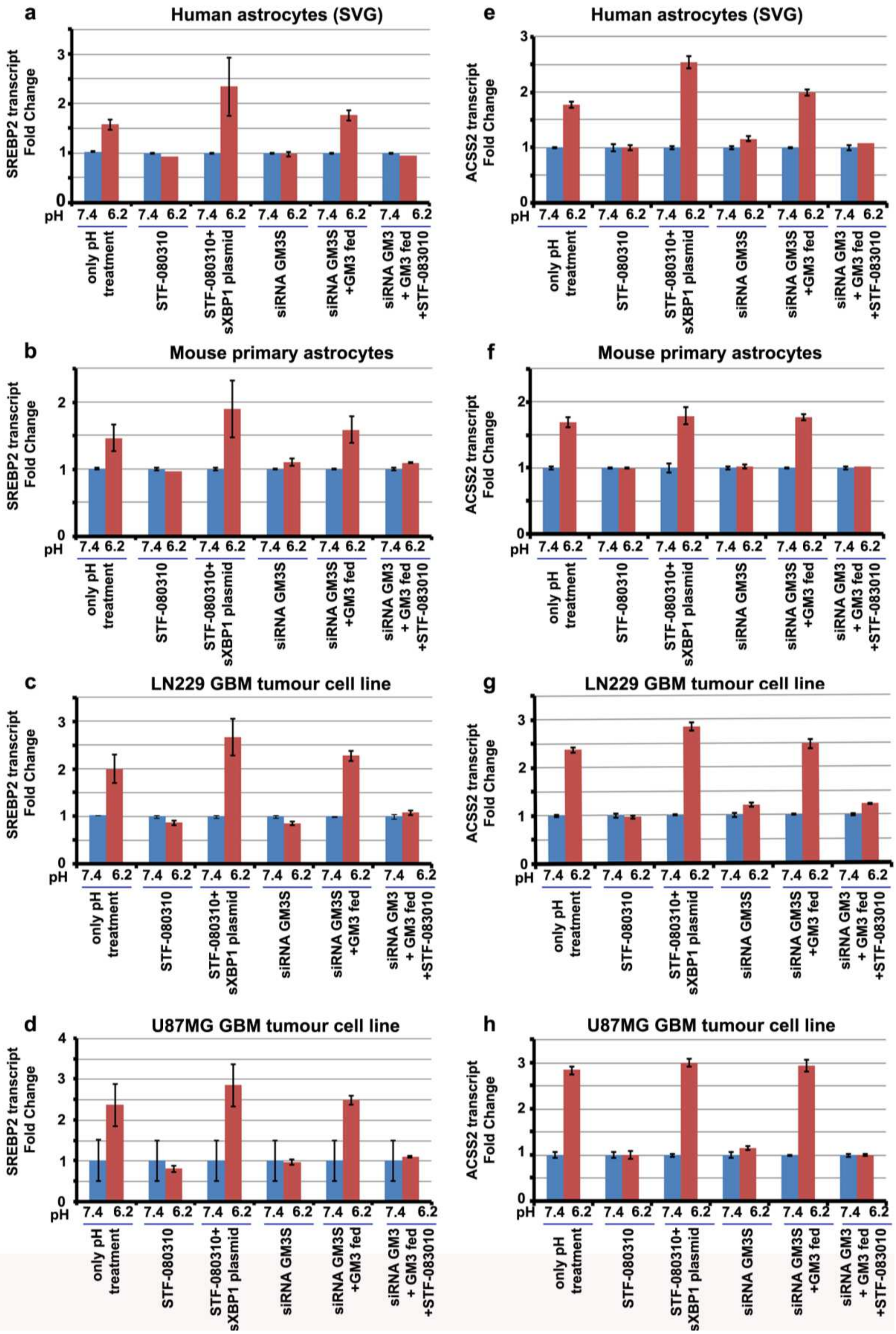

Fig. S33: SREBP2 and ACSS2 transcript levels confirm that these are the essential downstream pro-survival targets of GM3-sXBP1 low pH adaptation machinery in astrocytes and astrocytic tumours:

(a-d) Astrocytes (human and mouse primary) and astrocytic tumour cells (LN229 and U87MG) were treated with sXBP1 inhibitor, STF-083010 for 2 hours before pH treatments. STF-083010 was further added in media adjusted to normal or low pH. Cells were treated with the pH±STF for 8 hours.

(e-h) In another condition, cells were priorly transfected with sXBP1 plasmid, but sXBP1 generation via IRE1 RNAse activity was kept inhibited through treatment with STF-083010.

In these conditions, real-time PCR analysis of SREBP2 and ACSS2 showed that so far, sXBP1 was present in cells, SREBP2 and ACSS2 were transcribed, more significantly in low pH incubated cells. Further, in GM3 depleted cells, SREBP2 and ACSS2 transcripts were significantly less. In GM3 depleted cells that were fed with exogenous GM3, SREBP2 and ACSS2 transcripts were significantly upregulated. However, this was not so if GMS siRNA+ GM3 fed condition had depletion of sXBP1 (via STF-083010).

The result clearly suggests that SREBP2 and ACSS2 are crucially upregulated via GM3 and sXBP1 upstream axis.

All datasets are reported as mean± SD. Significance is shown as *p<0.05, **p<0.01, ***p<0.001. Mean is derived from 3 independent experiments.

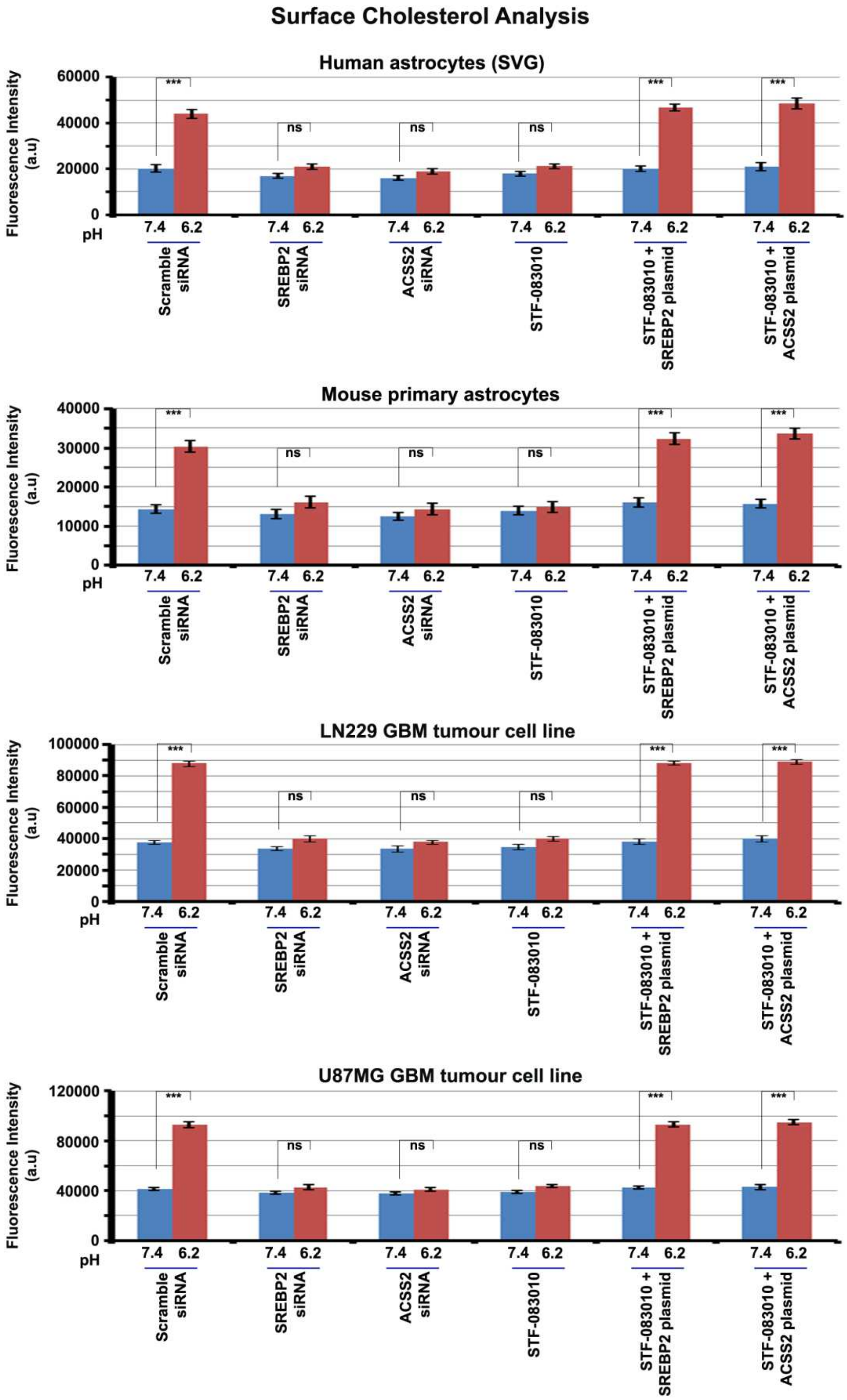

Fig.S34: SREBP2 and ACSS2 are crucial downstream targets of sXBP1 in the enhancement of excess surface cholesterol in low pH treated astrocytes and astrocytic tumour cells:

(a-b) Astrocytes (human and mouse primary) as well as **(c-d)** astrocytic tumour cells (LN229 and U87MG) were depleted of SREBP2 and ACSS2 transcript via siRNA. sXBP1 depletion

was performed via treatment with STF-083010. sXBP1 depleted cells were independently transfected with SREBP2 or ACSS2 plasmids to test their rescue role. The cells in these experimental set-ups were incubated with normal or low pH microenvironments. The system was probed for the levels of surface cholesterol via Nystatin staining and confocal imaging followed with fluorescence intensity quantification in Fiji Software. Image acquisition parameters were kept the same across conditions.

The result shows that surface cholesterol is significantly less in sXBP1, SREBP2 and ACSS2 depleted low pH conditions. However, in sXBP1 depleted low pH conditions, plasmid-mediated expression of sXBP1 downstream targets SREBP2/ACSS2 could restore the levels of surface cholesterol. Therefore, SREBP2 and ACSS2 are crucial downstream targets of sXBP1 in the enhancement of excess surface cholesterol in low pH treated astrocytes and astrocytic tumour cells.

At least 200 cells from random fields were taken for single-cell measurements, in each pH condition, from 3 independent experiments. All datasets are reported as mean± SD. Significance is shown as *$p<0.05$, **$p<0.01$, ***$p<0.001$. Mean is derived from 3 independent experiments.

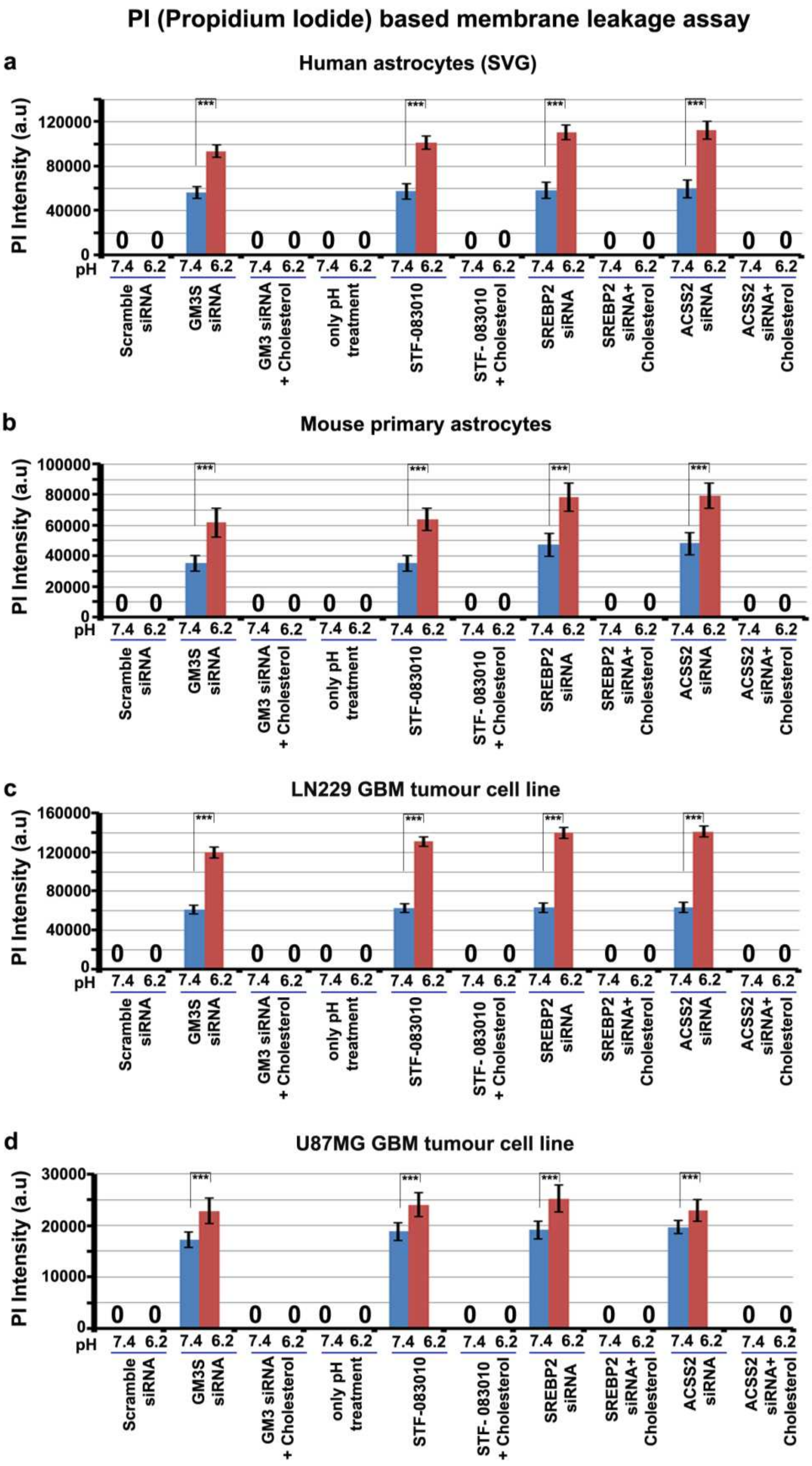

Fig. S35: Propidium Iodide (PI) based membrane leakage assay shows that cholesterol is an essential pro-survival responder in low pH adaptation machinery in astrocytes and astrocytic tumours:

(a-d) In order to validate that cholesterol was the final saviour in GM3-sXBP1-SREBP2-ACSS2 acid stress adaptation axis in astrocytes (human and mouse primary) and astrocytic tumour cells (LN229 and U87MG), we supplemented the plasma membrane with exogenous cholesterol (150µM, 2 hrs) in (i) GM3 depleted cells [GM3S siRNA] (ii) sXBP1 depleted cells [STF-083010 treated cells], (iii) SREBP2 depleted cells [SREBP2 siRNA] and (iv) ACSS2 depleted cells [ACSS2 siRNA]. Post this; pH treatments were given for 8 hours. The PI assay was performed to understand whether cholesterol supplementation could rescue leakage in the condition described in (i)-(iv).

It is clear that cholesterol feeding was sufficient to prevent leakage in GM3, sXBP1, SREBP2, and ACCS2 inhibited cells. Hence, GM3-sXBP1-SREBP2-ACSS2 major pro-survival function in astrocytes and astrocytic tumour cells can be attributed to their cholesterol biosynthesis activity.

All datasets are reported as mean ± SD. Significance is shown as *$p<0.05$, **$p<0.01$, ***$p<0.001$. Mean is derived from 3 independent experiments.

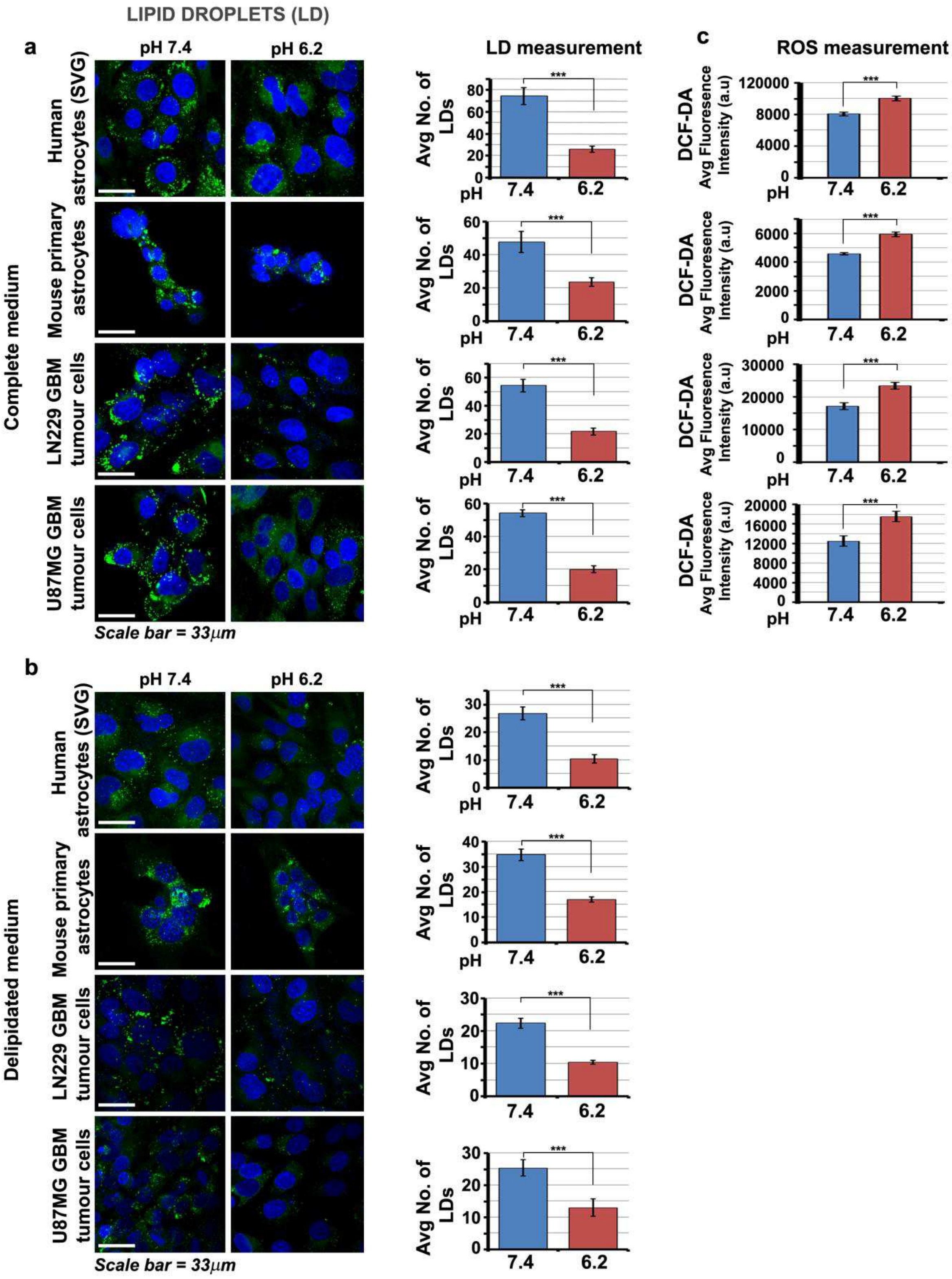

Fig. S36: Lesser lipid droplets (LD) accumulation was found in astrocytes/astrocytic tumours exposed to low pH conditions vs the physiological pH, although ROS levels were found to be higher in low pH conditions:

Astrocytes (human and mouse primary) and astrocytic tumour cells (LN229 and U87MG) were cultured in complete medium (with FBS) or delipidated medium (lipid depleted, without FBS) adjusted to normal or low pH. **(a-b)** Bodipy 493/503 was employed for live cell detection of lipid droplets in the above experimental conditions.

Results show that astrocytes and astrocytic tumours in low pH conditions have significantly less lipid droplet formation. Please note that in the complete medium, the lipid droplets observed are the sum total of lipids uptaken from media LDL particles and endogenous lipid generation. Lipid droplets in delipidated medium represent transient storage due to endogenous lipid generation.

(c) In an independent set, live cells DCF-DA assay (cat. No. C2938, Invitrogen) was employed according to the manufacturer's instructions to probe fluorescent reactive oxygen species (ROS) signal. Image acquisition parameters were kept the same across conditions.

The results clearly show that despite higher levels of ROS in low pH incubated cells, no significant deleterious lipid droplet accumulation (foam cell formation) occurs in astrocytes and astrocytic tumours in physiological or low pH treatments. Therefore, unlike in other cell types, such as in tumour-associated dendritic cells (DCs), astrocytes and astrocytic tumour cells do not allow accumulation of excessive LD's in the presence of ROS.

At least 200 cells from random fields were taken for single-cell measurements, in each pH condition, from 3 independent experiments. All datasets are reported as mean ± SD. Significance is shown as *$p<0.05$, **$p<0.01$, ***$p<0.001$. Mean is derived from 3 independent experiments.

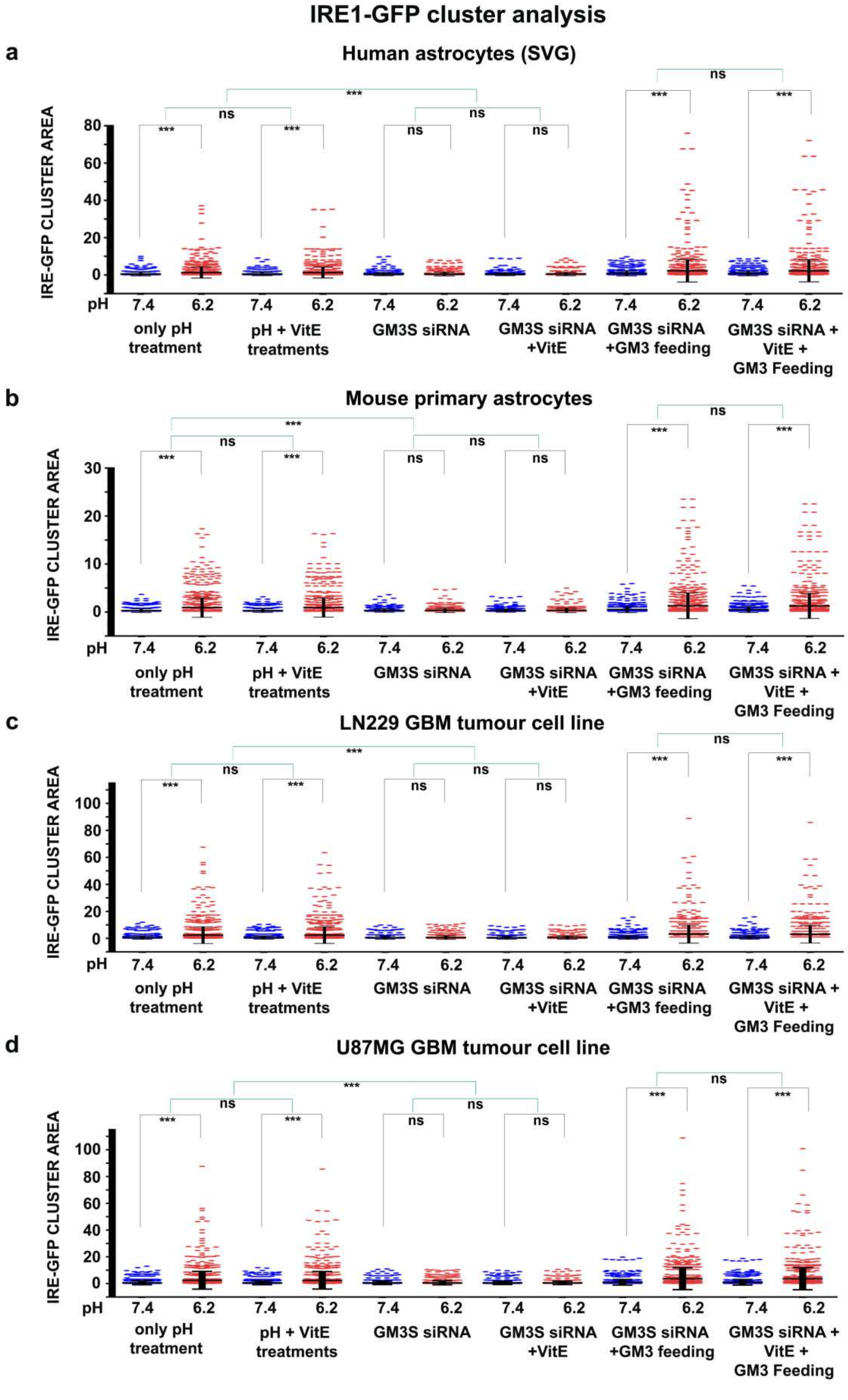

Fig. S37: GM3 and <u>not</u> ROS enhances IRE-1 clustering/activation in low pH treated astrocytes and astrocytic tumours.

(a-b) Astrocytes (human and primary) and **(c-d)** astrocytic tumour cells (LN229 and U87MG) were transfected with low dose doxycycline-inducible (10nM) GFP tagged IRE1

plasmid. The IRE1 was induced 16 hours before pH treatments. The pH treatments were given with or without VitE (100µM) to understand the impact of ROS in IRE1 activation. Vitamin E quenches reactive oxygen species (ROS). The pH treatments were also given with or without VitE in GM3 depleted, and GM3 repleted conditions, to understand the impact of GM3 vs ROS in IRE1 activation. IRE1-GFP cluster size measurements were performed in astrocytes and astrocytic tumours in the following experimental set-ups. Clustering of IRE1 indicates its activation.

Results show significant IRE1 clustering (oligomerization) at low pHe values, whereas this potency was lost in astrocytes depleted of GM3 (GM3S siRNA). However, the clustering was preserved in GM3 depleted astrocytes that were exogenously fed with GM3 lipid. VitE treatment (ROS depletion) did not show any significant effect on IRE1 activation. Therefore, results show that GM3 is required for IRE1 oligomerization in low pH conditions. Although ROS was higher in the acidic microenvironment **(Fig. S36)**, it could not promote IRE1 clustering in the absence of GM3.

At least 70 cells from random fields were taken for single-cell measurements, in each pH condition, from 3 independent experiments. All datasets are reported as mean± SD. Significance is shown as *$p<0.05$, **$p<0.01$, ***$p<0.001$. Mean is derived from 3 independent experiments.

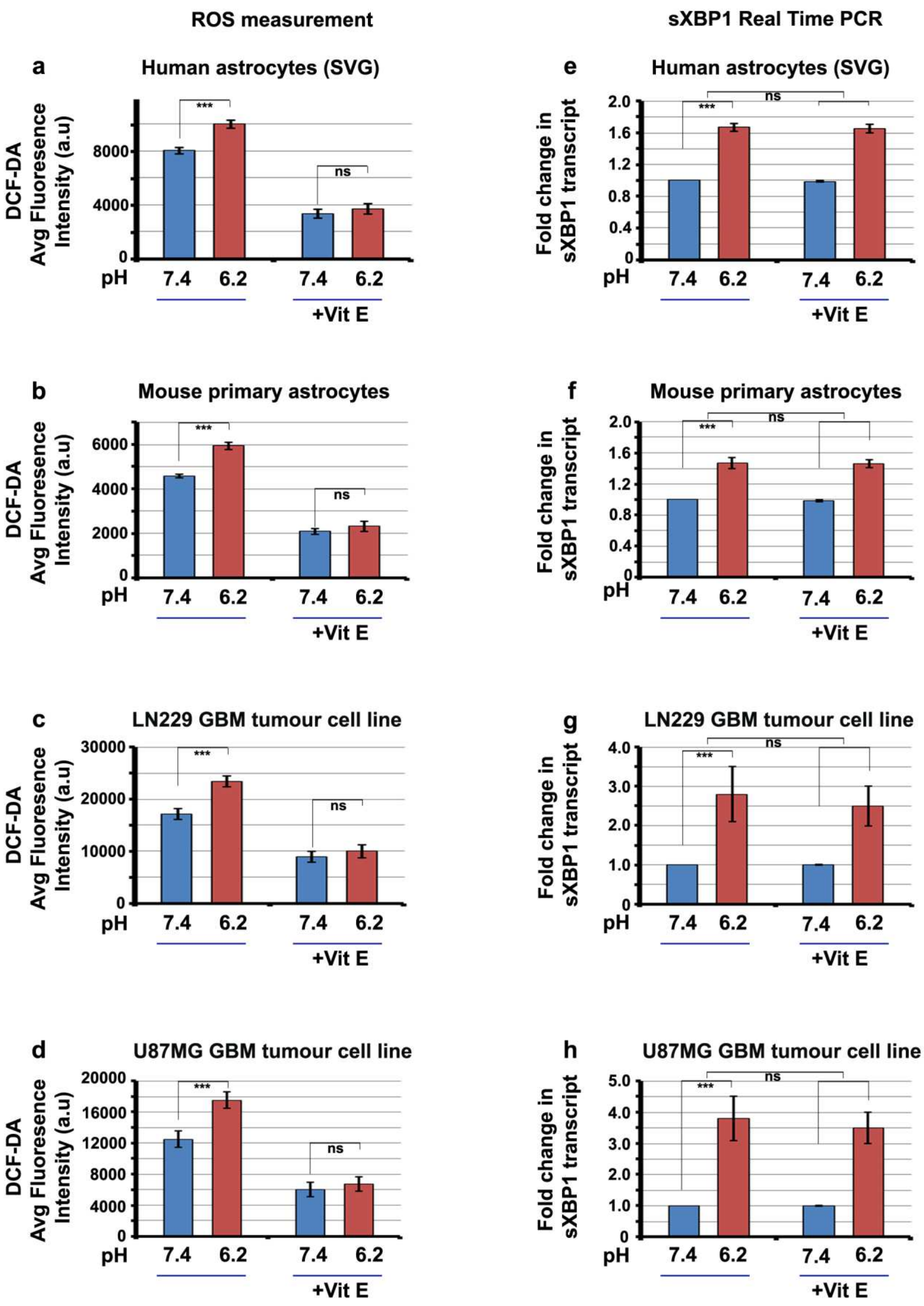

Fig. S38: ROS does <u>not</u> impact sXBP1 transcript levels in low pH treated astrocytes (human and mouse primary) and astrocytic tumours (LN229 and U87MG).

Astrocytes (human and primary) and astrocytic tumour cells were given pH treatments with or without VitE (100µM), a ROS inhibitor, to understand the impact of ROS in sXBP1 activation. **(a-d)** Live cell DCF-DA assay was employed to probe fluorescent reactive oxygen species (ROS) signal. Image acquisition parameters were kept the same across conditions.

The results clearly show that ROS was lowered with VitE treatment, more so in low pH incubated cells, **(e-h)** RNA was isolated in an independent set of experiments, and real-time PCR was performed to probe for sXBP1 transcript. Results clearly show that ROS inhibition did not affect sXBP1 generation. As demonstrated in **Fig.4a** and **Fig. 4f**, inhibition of GM3 synthesis showed very low levels of sXBP1 in low pH conditions. Hence, sXBP1 is an essential downstream effector of GM3 and not ROS.

All datasets are reported as mean± SD. Significance is shown as *p<0.05, **p<0.01, ***p<0.001. Mean is derived from 3 independent experiments.

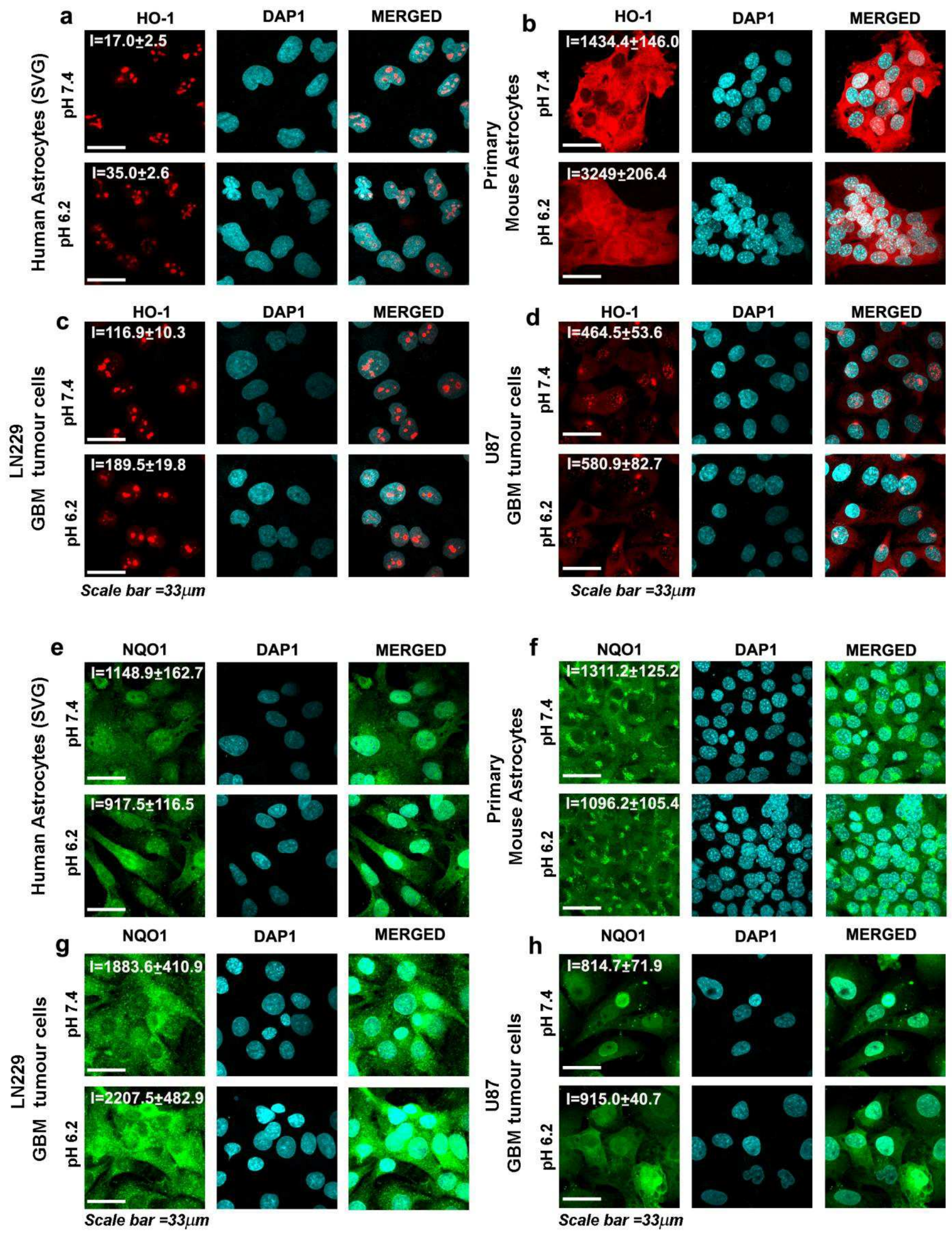

Fig. S39: HO-1 and NQO1, the downstream anti-oxidant targets of ROS are activated in acidified astrocytes and astrocytic tumour cells.

HO-1 and NQO1 crucially degrade ROS toxic intermediate HNE and thereby is involved in cytoprotection. Astrocytes (human and mouse primary) and astrocytic tumour cells (LN229

and U87MG) were treated with physiological and low pH media for 8 hrs. Post incubation, cells were fixed in 1.5% PFA, washed in 1X PBS, permeabilised with 0.25% saponin and incubated with anti-HO1 and anti-NQO1 antibodies for immunolabelling at 4°C for 16 hrs.

(a-d) HO-1 and **(e-h)** NQO1 were expressed in both normal and low pH conditions. Both proteins were found to be significantly upregulated in low pH conditions. Imaging in each condition was performed in a confocal microscope.

Image acquisition conditions in each channel were kept the same in each condition over independent experiments. At least 50-70 cells from random fields were taken for single-cell measurements, in each pH condition, from 3 independent experiments. The expression of each antigen was analysed in Fiji software. Fluorescence intensities are shown in respective panels for comparison. I= Mean Fluorescence Intensity in arbitrary units.

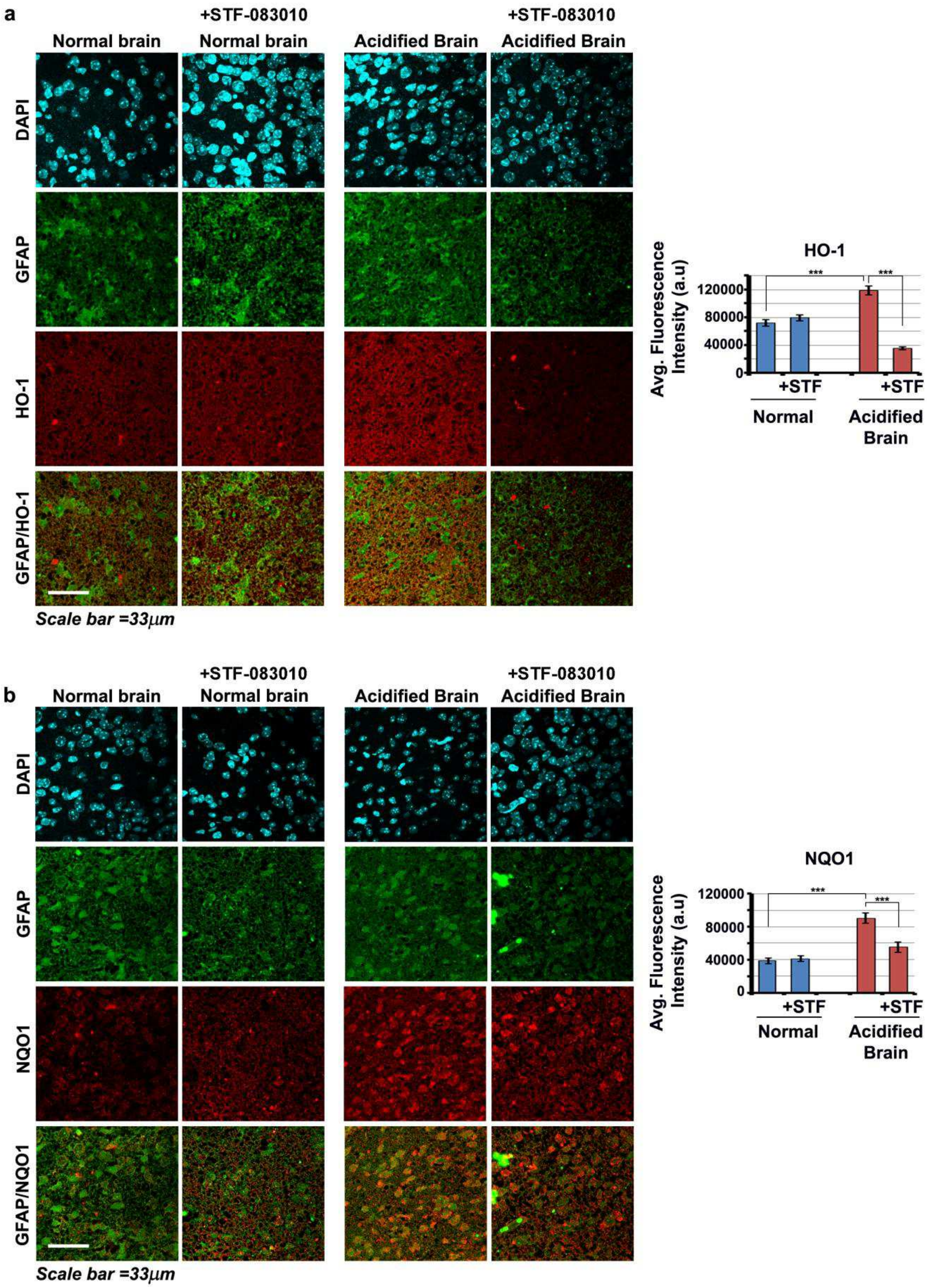

Fig. S40: HO-1 and NQO1, the downstream anti-oxidant targets of ROS, are inhibited by STF-083010 treatment in acidified brains.

Mouse brain acidification was generated by 7% CO2 inhalation for 2.5 hours. Mice were simultaneously either subcutaneously injected with STF-083010 or not. See the methods section for more details.

(a,b) HO-1 and NQO1 are upregulated by the cytoprotective arm of the ROS (reactive oxygen species) stress. These are activated to degraded ROS downstream intermediate, HNE. Acidified mouse brain showed significant levels of these cytoprotective proteins in the astrocytes from acidified brain vs normal brain, which was not so in STF-083010 injected acidified brains. GFAP is an astrocyte marker.

HO-1 and NQO1 expression were quantified by measuring fluorescence intensity in 5 random fields per section per 3 independent mouse brains, using Fiji software. The brain regions used for imaging and intensity quantification per section were kept the same across independent brains across antigen.

All datasets are reported as mean± SD. Significance is shown as *p<0.05, **p<0.01, ***p<0.001. Mean is derived from 3 independent experiments.

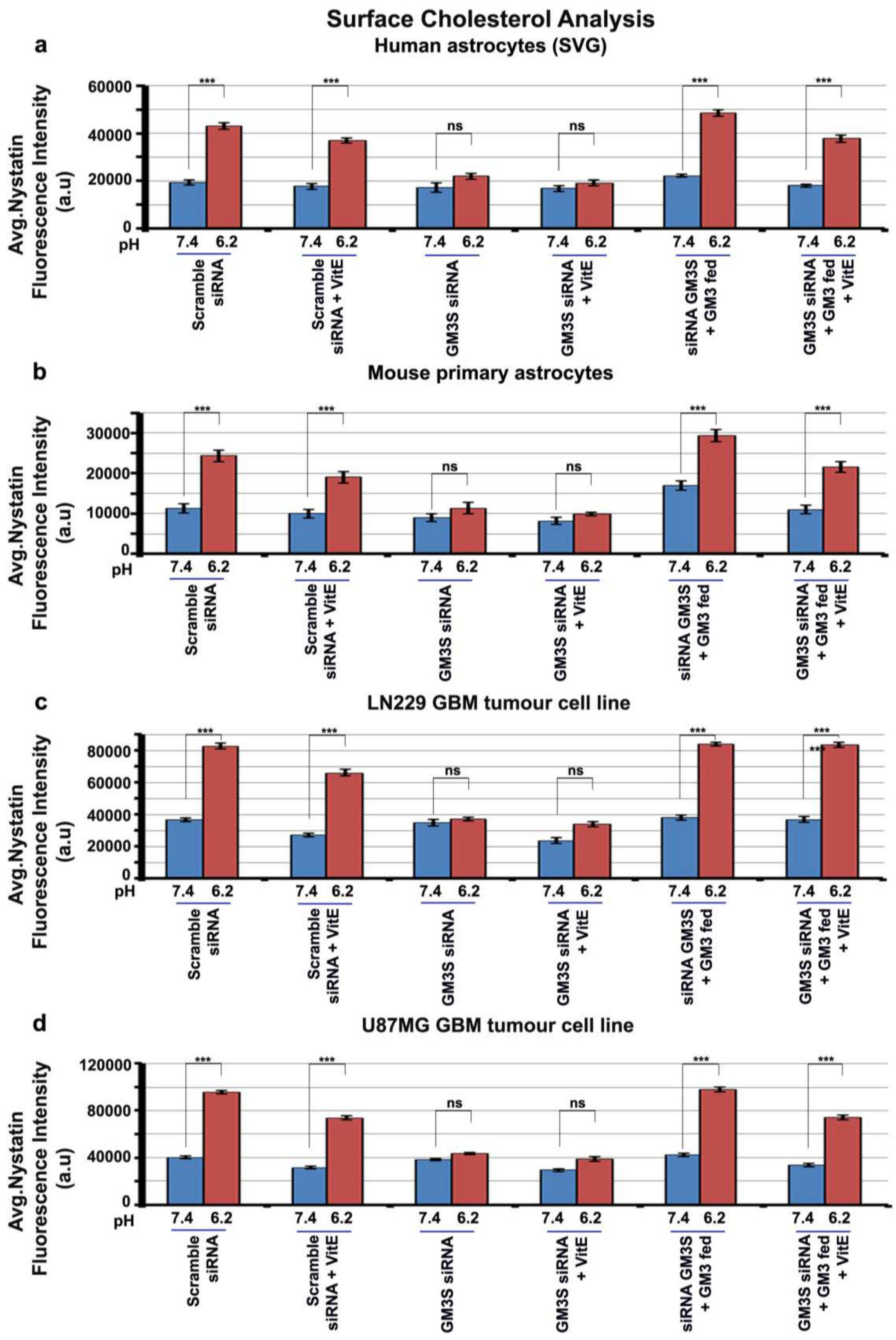

Fig. S41: GM3 and <u>not</u> ROS enhances surface cholesterol in low pH treated astrocytes and astrocytic tumours.

(a-b) Astrocytes and **(c-d)** astrocytic tumours were depleted of GM3 via siRNA of its synthetic enzyme GM3S. GM3 depleted cells were fed with an exogenous source of GM3

lipid in an independent set. The cells were incubated with normal or low pH medium with or without Vitamin E (ROS inhibitor, conc. 100µM)). Nystatin staining was employed to quantitate surface cholesterol.

Results show that ROS could not majorly affect surface cholesterol levels in low pH incubated cells. Although slightly lower levels of surface cholesterol were noticed in VitE treatment as VitE is shown to inhibit cholesterol synthesis enzymes. However, no major decrease in surface cholesterol was noticed. GM3 depletion had a major impact on the reduction of surface cholesterol. Hence, GM3 and not ROS is associated with enhancement of surface cholesterol levels in low pH stress.

Image acquisition parameters were kept the same across conditions. At least 200 cells from random fields were taken for single-cell measurements, in each pH condition, from 3 independent experiments. All datasets are reported as mean± SD. Significance is shown as *$p<0.05$, **$p<0.01$, ***$p<0.001$. Mean is derived from 3 independent experiments.

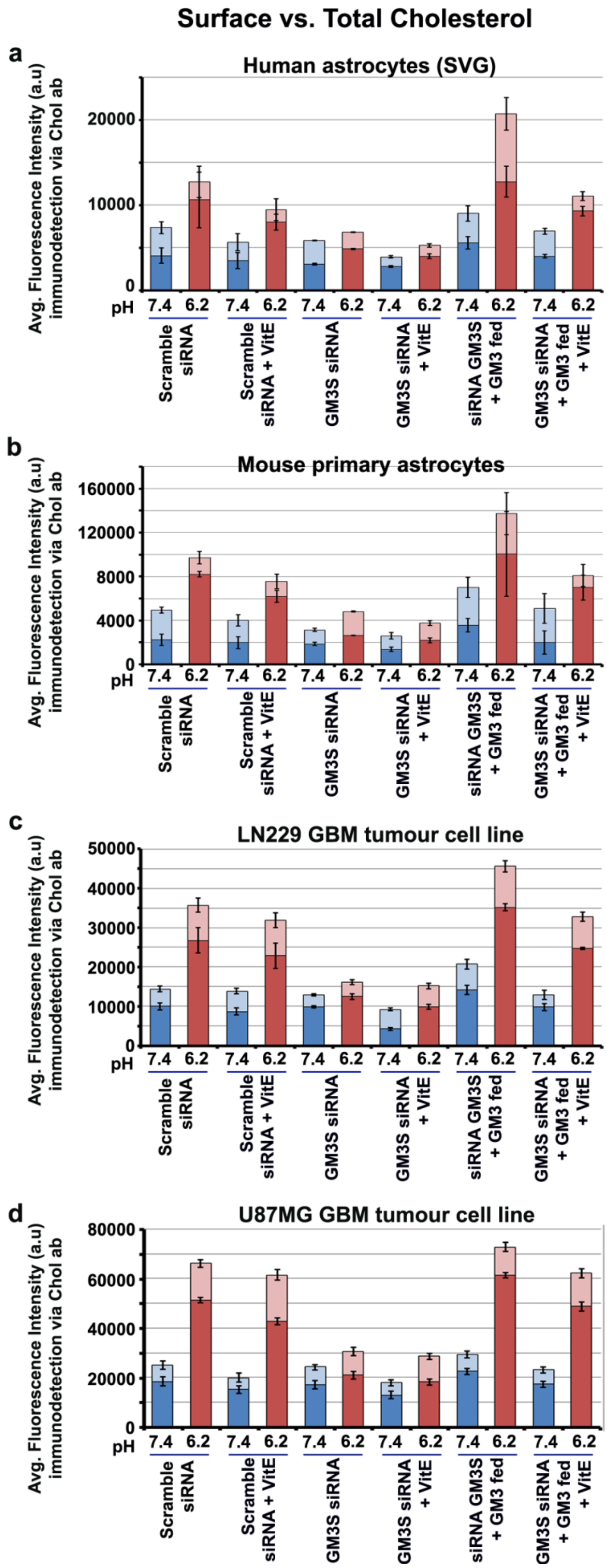

Fig. S42: GM3 and <u>not</u> ROS enhances surface cholesterol trafficking in low pH treated astrocytes and astrocytic tumours.

(a-b) Astrocytes (human and mouse primary) and **(c-d)** astrocytic tumours (LN229 and U87MG) were depleted of GM3 via siRNA of its synthetic enzyme GM3S. GM3 depleted cells were fed with an exogenous source of GM3 lipid in an independent set. The cells were incubated with normal or low pH medium with or without Vitamin E (ROS inhibitor, conc. 100µM). Cholesterol antibody was used to quantitate surface vs total cholesterol in each of the above described experimental set-ups (please see supplementary methods for details).

We find an overall increase in cholesterol biogenesis in those low pH conditions wherein GM3 was present. This observation corroborated with no significant endogenous accumulation of cholesterol intermediates as lipid droplets were, in fact, far less in acidic conditions **(Fig. S36)**. The increased surface cholesterol fraction in acidic pH suggests that raised total cholesterol was trafficked mainly to the surface.

Results show that ROS could not majorly affect surface cholesterol levels in low pH incubated cells. Although slightly less total and surface cholesterol levels were noticed in VitE treatment as VitE is shown to inhibit cholesterol synthesis enzymes. However, no major decrease in surface cholesterol was noticed. GM3 depletion had a major impact on the reduction of surface cholesterol. Hence, GM3 and not ROS is associated with enhancement of surface cholesterol levels in low pH stress.

Image acquisition parameters were kept the same across conditions. At least 200 cells from random fields were taken for single-cell measurements, in each pH condition, from 3 independent experiments. All datasets are reported as mean± SD. Significance is shown as *$p<0.05$, **$p<0.01$, ***$p<0.001$. Mean is derived from 3 independent experiments.

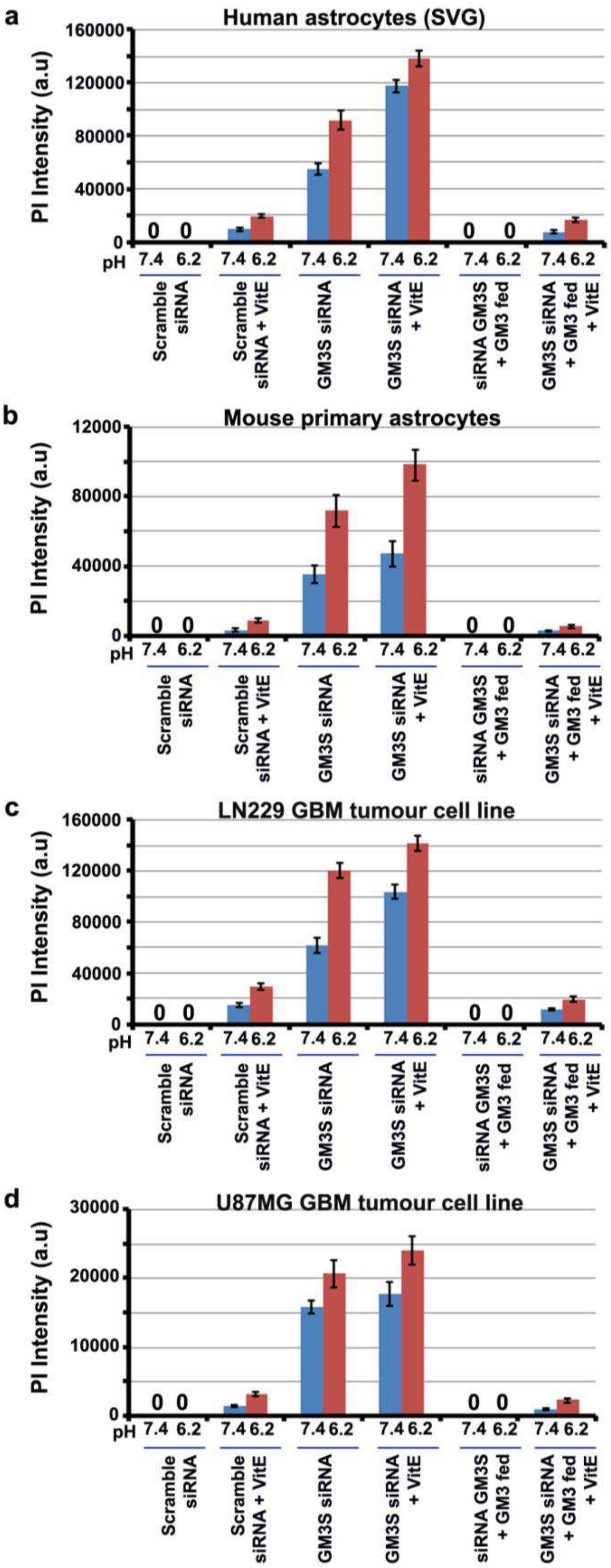

Fig. S43: Depleting GM3 and not ROS causes membrane leakage in pH treated astrocytes and astrocytic tumours.

(a-b) Astrocytes and **(c-d)** astrocytic tumours were depleted of GM3 via siRNA of its synthetic enzyme GM3S. GM3 depleted cells were fed with an exogenous source of GM3 lipid in an independent set. The cells were incubated with normal or low pH medium with or without Vitamin E (ROS inhibitor, conc. 100μM)). Propidium Iodide (PI) uptake assay was performed in the above experimental set-ups.

The data clearly shows that GM3 was predominantly required for the prevention of low pH mediated acid hydrolysis/membrane leakage and not ROS. Even though ROS levels were found to be higher in astrocytes/astrocytic tumours exposed to low pH conditions **(Fig. S36)**, inhibition of ROS did not dramatically impact membrane leakage, although mild leakage ensued even in GM3 enriched cells. This could be majorily because ROS plays a crucial role in fluidizing the membrane and does not allow the membrane to become alarmingly rigid in pathological conditions such as acidosis. Hence, loss of ROS may make the membrane mildly leaky, but ROS by itself could not prevent loss of membrane integrity under acidosis. However, GM3 was found to be imperative for the prevention of acid-mediated membrane leakage.

Image acquisition parameters were kept the same across conditions. At least 200 cells from random fields were taken for single-cell measurements, in each pH condition, from 3 independent experiments. All datasets are reported as mean± SD. Significance is shown as *p<0.05, **p<0.01, ***p<0.001. Mean is derived from 3 independent experiments.

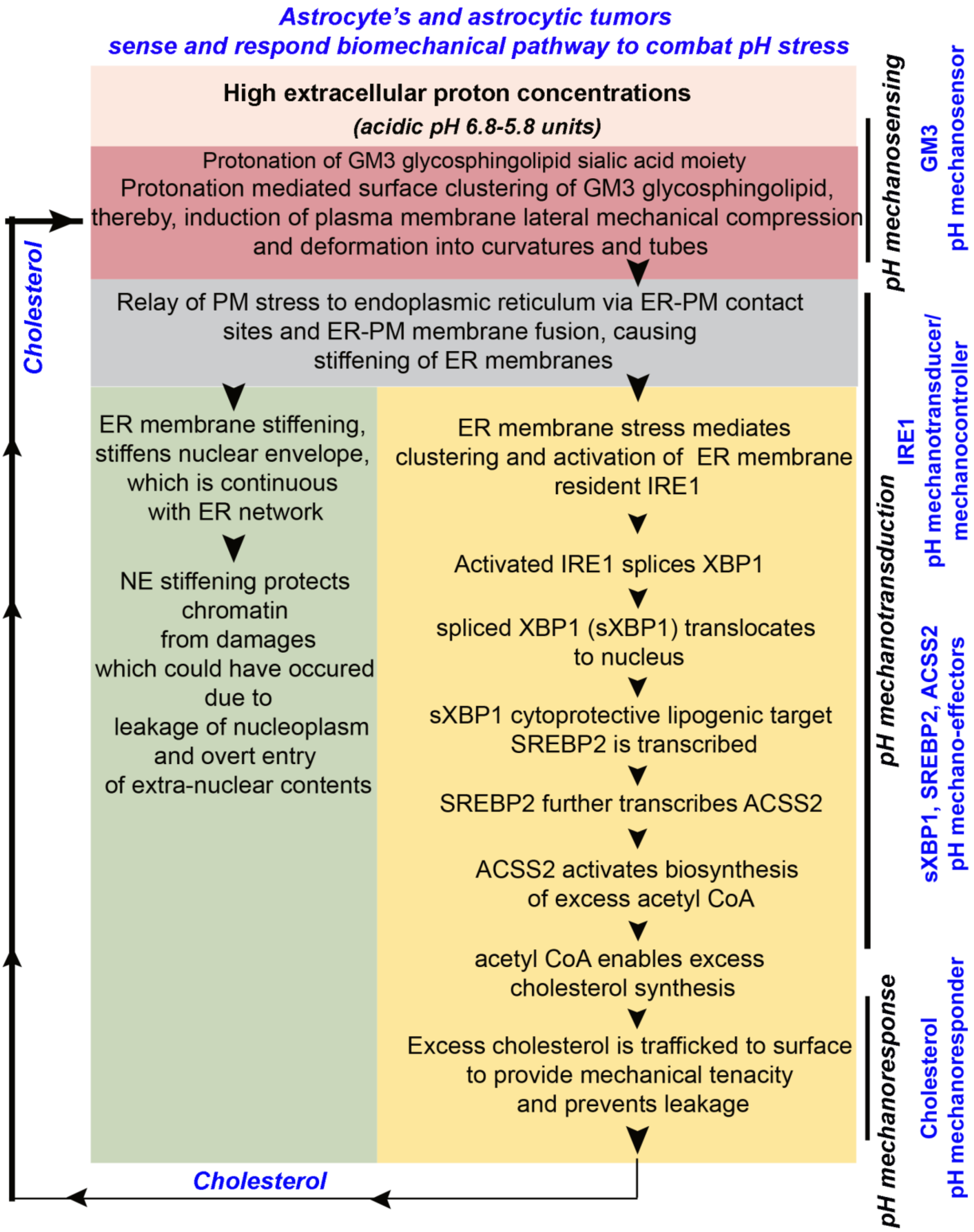

Fig. S44: Self-explanatory flowchart representation of the sense and respond strategies generated by astrocytes and brain tumours of astrocytic origins to combat extracellular pH stress.
